# Behavior Regulation of *CO over Self‐Evolution Tandem Catalysts Under Tuned Interfacial Electric Field Boosts CO_2_ Electroreduction

**DOI:** 10.1002/anie.202511704

**Published:** 2025-08-19

**Authors:** Zining Zhang, Xinyan Ma, Yang Song, Xue Yang, Qi Fang, Yusuke Yamauchi, Jing Tang

**Affiliations:** ^1^ State Key Laboratory of Petroleum Molecular & Process Engineering Shanghai Key Laboratory of Green Chemistry and Chemical Processes School of Chemistry and Molecular Engineering East China Normal University Shanghai 200062 China; ^2^ State Key Laboratory of Petroleum Molecular & Process Engineering Sinopec Research Institute of Petroleum Processing Co., LTD. Beijing 100083 China; ^3^ Australian Institute for Bioengineering and Nanotechnology (AIBN) The University of Queensland Brisbane QLD 4072 Australia; ^4^ Department of Materials Process Engineering, Graduate School of Engineering Nagoya University Nagoya Aichi 464–8603 Japan; ^5^ Institute of Eco‐Chongming Shanghai 202162 China

**Keywords:** Carbon dioxide reduction, Electrocatalysis, Interfacial electric field, Multicarbon chemicals, Tandem catalysts

## Abstract

Tandem or self‐evolution Cu‐based catalysts effectively regulate *CO to promote the conversion of CO_2_ electroreduction to multicarbon (C_2+_) products. DFT calculations reveal that the adsorption capacity of *CO varies under different interfacial electric field intensities for the Cu, Ag/Cu, Pd/Cu, and Au/Cu models. Accordingly, we design three kinds of self‐evolution tandem catalysts and investigate the adsorption and migration behaviors of *CO under interfacial electric fields. Electrochemical CO_2_ reduction test results indicate that the higher CO selectivity of Au/Cu is attributed to its weak *CO adsorption capacity, confirmed by in situ attenuated total reflection‐infrared and in situ Raman. The low C_2+_ selectivity of Pd/Cu is owing to its high reaction energy barrier and low catalytic activity. In contrast, Ag/Cu achieves a high FE_C2+_ of 89.2% and a partial current density (j_c2+_) of 553.9 mA cm^−2^ thanks to the low reaction energy barrier and moderate *CO adsorption capacity. COMSOL multiphysics simulations reveal that the effect of the interfacial electric field on *CO external migration could be neglected in the nanometer range. Although a strong interfacial electric field increases the energy barrier for internal migration of *CO, the enhanced adsorption capacity of *CO still dominates C–C coupling in the *CO‐rich microenvironment over tandem catalysts.

## Graphical abstract

In this work, three kinds of self‐evolution tandem catalysts (Ag/Cu, Pd/Cu, and Au/Cu) are designed and the adsorption and migration behaviors of *CO are evaluated by DFT calculations, COMSOL multiphysics simulations and in situ characterizations. The research figures out that although strong interfacial electric fields weaken the internal migration of *CO, the enhanced adsorption capacity for *CO is a key factor for C–C coupling in tandem catalysts.

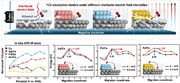

## Introduction

Electrochemical CO_2_ reduction (ECR) technology utilizes green electricity as a driving force to convert CO_2_ into high‐value chemicals under mild conditions, which is of great significance for the green transformation of the energy structure.^[^ ^1^, ^2^ ^]^ Multicarbon (C_2+_) products such as ethylene (C_2_H_4_),^[^ ^3^, ^4^, ^5^ ^]^ ethanol (EtOH),^[^ ^6^, ^7^, ^8^ ^]^ and propanol (PrOH)^[^ ^9^, ^10^ ^]^ are gradually gaining attention due to their high added value and industrial potential. However, how to achieve high selectivity and large current density for C_2+_ products still faces many difficulties.^[^ ^11^, ^12^, ^13^ ^]^ One of the more important factors is the low coverage of *CO intermediate for Cu‐based catalysts, which leads to unfavorable C–C coupling and impedes the generation of C_2+_ products.^[^ ^14^, ^15^ ^]^ Hence, enhancing the adsorption capacity of *CO by optimizing the catalyst design and regulating the reaction microenvironment are important approaches to increasing the coverage of *CO.

Constructing a bimetallic tandem system is one effective strategy to enhance the coverage of *CO over Cu‐based catalysts.^[^ ^16^, ^17^, ^18^, ^19^ ^]^ The tandem elements (such as Ag,^[^ ^20^, ^21^ ^]^ Pd,^[^ ^22^, ^23^ ^]^ Au,^[^ ^24^, ^25^ ^]^ Zn,^[^ ^26^, ^27^ ^]^ and Ni, etc.^[^ ^28^, ^29^ ^]^) are in favor of converting CO_2_ to CO, which overflows to the adjacent Cu active sites and further couples to C_2+_ products. For instance, Chen et al. constructed a Ag–Cu tandem catalyst on a gas diffusion electrode, where the rich *CO microenvironment generated by Ag can promote the generation of C_2+_ products on Cu.^[^ ^30^ ^]^ Synthesizing Cu‐based catalyst with adjusted defects,^[^ ^31^ ^]^ lattice tensile stress,^[^ ^32^ ^]^ and coordination numbers^[^ ^33^, ^34^ ^]^ from self‐evolution Cu oxides is another important strategy to enhance the adsorption capacity of *CO over Cu active sites. Jiao et al. synthesized Cu catalysts with different coordination numbers through electrochemical self‐evolution methods (cyclic voltammetry, potentiostatic electrolysis, and pulsed electrolysis) from high‐valent Cu‐oxides. Among them, the Cu catalyst with a high coordination number is conducive to producing C_2+_ products with a Faradaic efficiency (FE) of 82.5%.^[^ ^35^ ^]^

Moreover, the interfacial electric field can also regulate the adsorption behavior of intermediates by altering the thermodynamic reaction energy barrier. Current researches mainly focus on the influence of interfacial electric field on CO_2_ intermediates, which are generally induced by morphology, cations, or additives.^[^ ^36^, ^37^, ^38^ ^]^ Liu and co‐workers coated Cu nanoneedles with polytetrafluoroethylene, leading to a strong tip electric field and promoting *CO accumulation. The FE_C2+_ reached 86% with a partial current density of more than 250 mA cm^−2^.^[^ ^39^ ^]^ Nevertheless, there is hardly research about the function of interfacial electric field on the adsorption and migration behaviors of *CO over Cu‐based catalysts. Therefore, it is still challenging to conjunct the advantages of self‐evolution, tandem catalysts, and interfacial electric field, then finally regulating the *CO intermediate and achieving excellent C_2+_ selectivity.

In this work, density functional theory (DFT) calculations were conducted to evaluate the adsorption of *CO on Cu and tandem catalysts under different interfacial electric fields. The variations of interfacial electric field intensities we referred to are controlled by the applied potentials.^[^ ^40^ ^]^ Then, three kinds of self‐evolution tandem catalysts (Ag/Cu, Pd/Cu, and Au/Cu) were synthesized and conducted constant potential ECR tests. COMSOL multiphysics simulations eliminate the influence of the electric field on *CO external migration. Our research figures out that although strong interfacial electric fields weaken the internal migration of *CO, the enhanced adsorption capacity for *CO is a key factor for C‐C coupling in tandem catalysts. Therefore, Au/Cu exhibits a higher CO selectivity, which is attributed to the weak adsorption capacity of *CO on its surface. In contrast, Ag/Cu exhibits a high C_2+_ selectivity (89.2%) with a partial current density (j_c2+_) of 553.9 mA cm^−2^ at –1.8 V versus RHE because of its moderate *CO adsorption capacity and lower reaction energy barrier. In situ attenuated total reflection‐infrared (ATR‐IR) and in situ Raman spectra further confirms the mutual relationship between interfacial electric field and *CO adsorption, which is consistent with our discussions.

## Results and Discussion

### DFT Calculations for Bindings Between *CO and Tandem Catalysts

**Figure 1:**
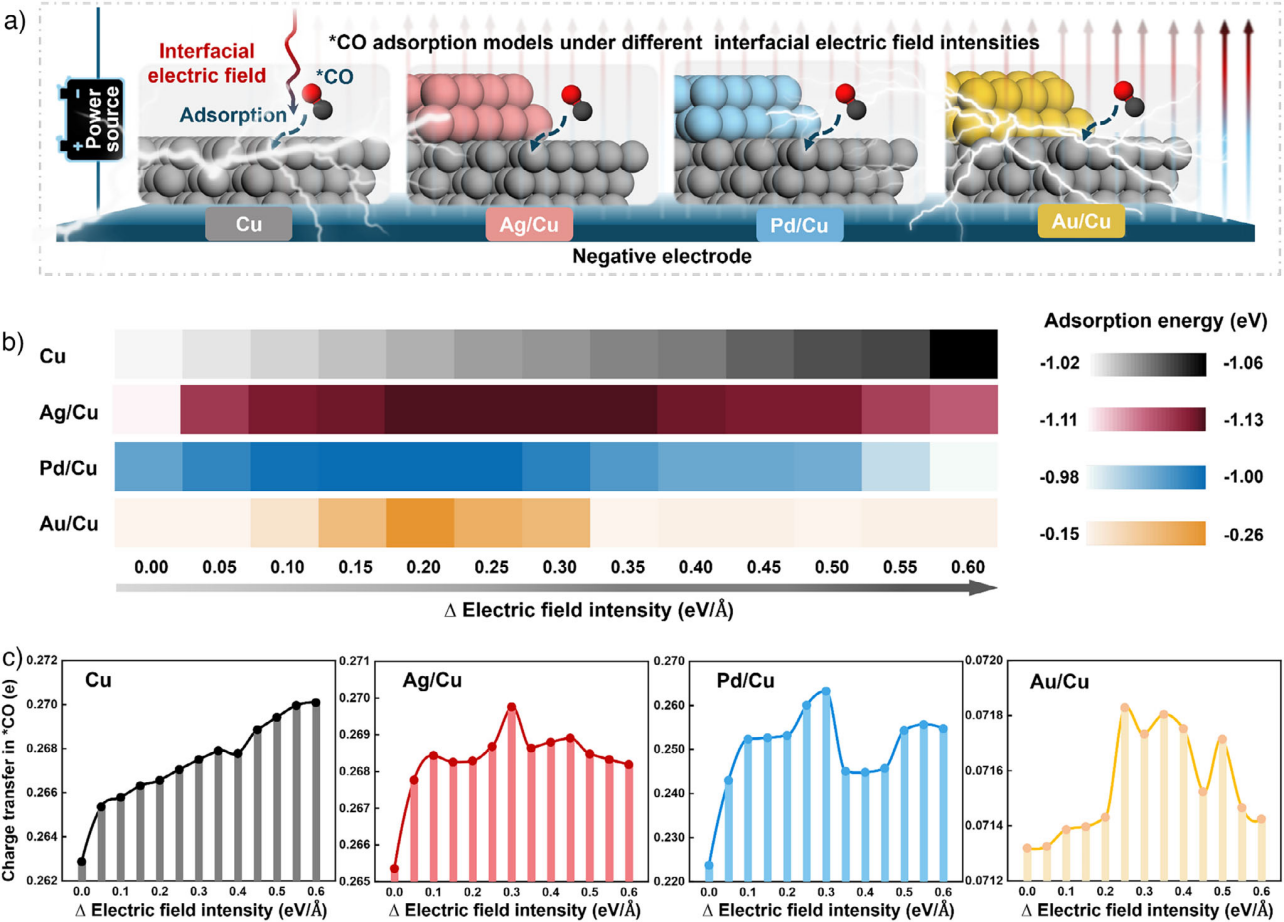
Calculations of *CO adsorption capacity on Cu, Ag/Cu, Pd/Cu, and Au/Cu under different electric field intensities. a) Schematic diagram for constructing *CO adsorption calculation models, b) *CO adsorption energy, c) *CO Bader charge transfer calculations for Cu, Ag/Cu, Pd/Cu, and Au/Cu under different electric field intensities (*CO represents adsorbed species).

Inspired by previous research on tandem catalysts, an interesting phenomenon attracts us that the selectivity of CO varies under different applied potentials.^[^ ^20^, ^21^, ^22^, ^24^ ^]^ To explore the relationship between the adsorption capacity of *CO in tandem catalysts and the interfacial electric field intensities, we selected three classic elements, Ag, Pd, and Au, which were commonly used in the study of tandem catalysts.^[^ ^41^, ^42^ ^]^ Figure 1a exhibits four calculation models for *CO adsorption under different electric field intensities, denoted as Cu, Ag/Cu, Pd/Cu, and Au/Cu. The specific calculation models and results are shown in Figures S1–S4 and Table S1. We overlooked the electrostatic field generated by cations and the solvation effect of water.^[^ ^43^ ^]^ As illustrated in Figure 1b, the adsorption energy of *CO for each catalyst varies along with the applied electric field. It is noteworthy that the adsorption energy of *CO on Au/Cu varies from –0.15 to –0.26 eV when the electric field intensities change from 0 to 0.6 eV Å^−1^. However, the adsorption energy of *CO on Cu and M/Cu (M = Ag, Pd) only varies within the range from –0.98 to –1.13 eV, which is quite lower than Au/Cu (–0.15 to –0.26 eV). The results imply that it is easier for *CO desorption over Au/Cu than Cu and M/Cu (M = Ag, Pd).

Bader charge analysis further reflects the strength of the binding ability between *CO and the catalysts. As shown in Figure 1c and Table S2, the electric field intensity affects the binding between *CO and the catalyst. It is worth noting that no matter how the electric field intensity changes, *CO always has fewer transferred electrons on Au/Cu, reflecting its weak binding on Au/Cu compared to Cu, Ag/Cu, and Pd/Cu. This weak binding force between *CO and Au/Cu may cause *CO to escape, which is not conducive to subsequent C–C coupling. According to the guidance provided by DFT calculations, choosing appropriate tandem elements and interfacial electric fields to construct tandem catalysts may result in excellent ECR performance.

### Synthesis and Characterization of Pre‐Catalysts (M/CuO Nanosheets)

**Figure 2:**
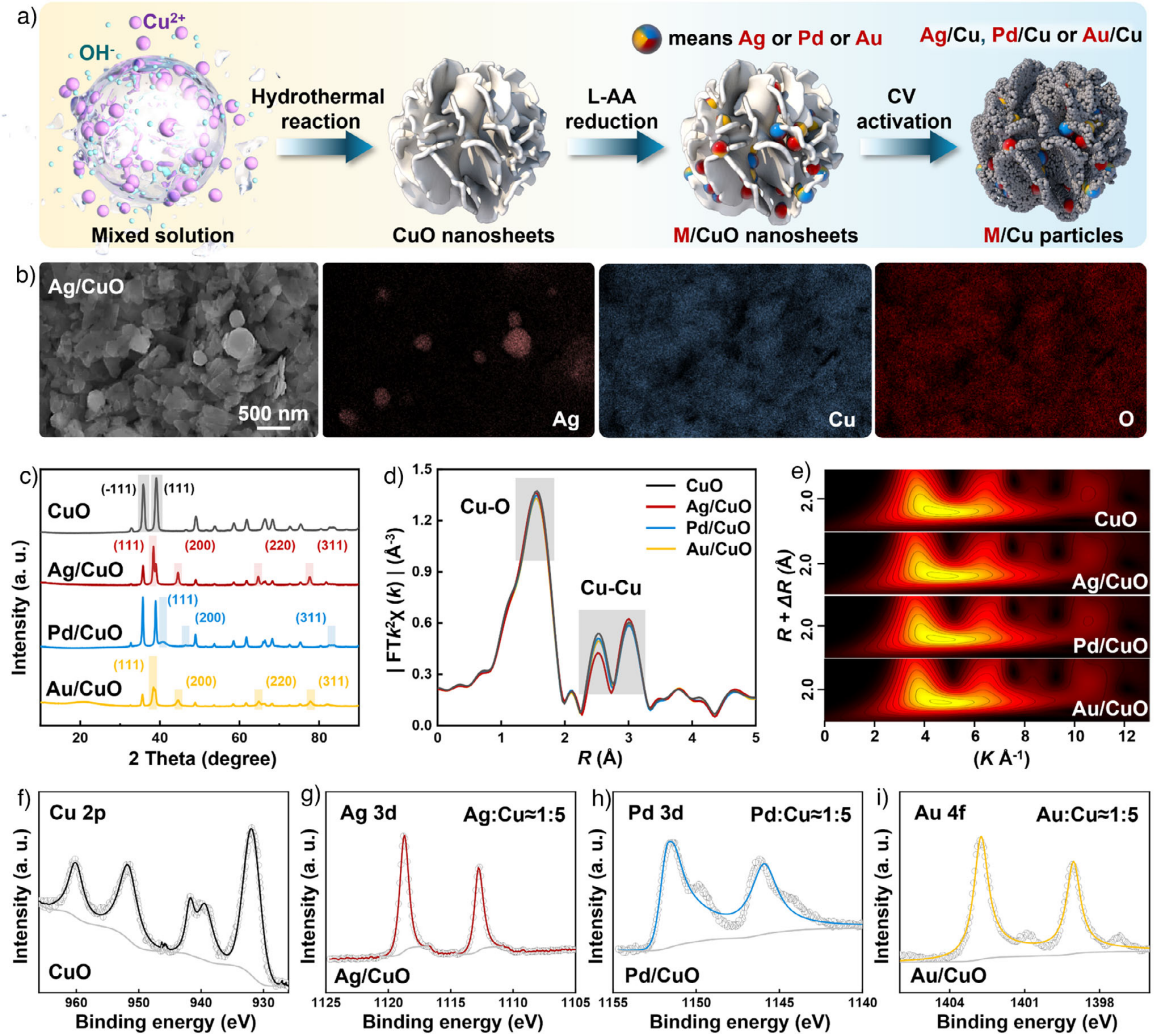
Preparation and characterization of CuO, Ag/CuO, Pd/CuO, and Au/CuO pre‐catalysts. a) Schematic illustration of the fabrication strategy for tandem catalysts. b) SEM image and elemental mappings of Ag/CuO. c) XRD patterns, d) FT‐EXAFS spectra, e) EXAFS wavelet transform plots, and f)–i) XPS spectra of CuO, Ag/CuO, Pd/CuO, and Au/CuO.

To prepare a series of different tandem catalysts via one‐step electrochemical self‐evolution method, we first synthesized M/CuO nanosheets as pre‐catalysts via a conventional hydrothermal approach and chemical reduction deposition method (Figure 2a). The pre‐catalysts are denoted as M/CuO nanosheets, where M represents Ag, Pd, or Au. Scanning electron microscopy (SEM) and energy dispersive X‐ray spectroscopy (EDS) reveal that Ag, Pd, or Au nanoparticles grew dispersedly over CuO nanosheets (Figure 2b and S5–S7). As illustrated in Figure 2c, peaks of X‐ray diffraction (XRD) at 38.7°, 38.1°, 40.4°, and 38.3° could be indexed to the crystalline planes (111) of CuO (PDF#89–5897), Ag (PDF#87–0597), Pd (PDF#01–1201), and Au (PDF#65–8601), respectively. High‐resolution transmission electron microscopy (HRTEM) images of CuO and M/CuO are shown in Figure S8. Extended X‐ray absorption fine structure spectroscopy (EXAFS) was utilized to explore the coordination environment of Cu in CuO and M/CuO catalysts. As shown in Figures 2d,e and S9–S13, Table S3, the coordination numbers of Cu─O and Cu─Cu bonds have not undergone changes in M/CuO compared to CuO catalysts. We further investigated the surface composition and contents of Cu and M elements in the catalysts through X‐ray photoelectron spectroscopy (XPS). As depicted in Figures 2f–i, we have detected distinct characteristic peaks related to Cu 2p, Ag 3d, Pd 3d, and Au 4f orbits in CuO and M/CuO catalysts, respectively. Furthermore, the atomic ratio of M to Cu was calculated and summarized in Figures S14–S16. Inductively coupled plasma optical emission spectroscopy (ICP‐OES) results were illustrated in Table S4. The above results show that the atomic ratio of M to Cu on the surface is similar in M/CuO catalysts, approximately being M_1_Cu_5_.

### Self‐Evolution of Tandem Catalysts and ECR Electrochemical Tests

**Figure 3:**
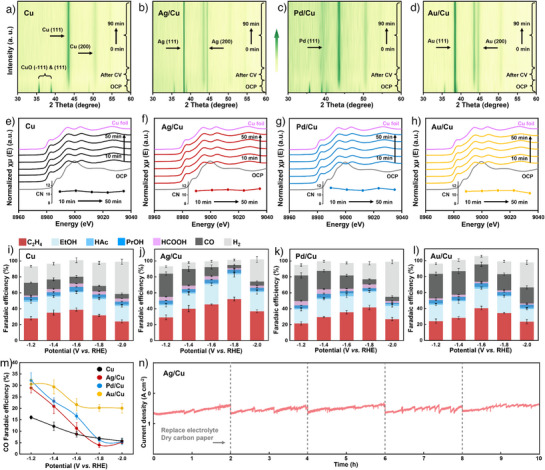
Self‐evolution process and performance of Cu, Ag/Cu, Pd/Cu, and Au/Cu catalysts at different potentials. a)–d) Contour maps of in situ XRD patterns, e)–h) Cu K‐edge XANES spectra over time during the ECR process by potentiostatic electrolysis (CN is the coordination number), i)–l) product distributions, and m) CO Faradaic efficiency of Cu, Ag/Cu, Pd/Cu, and Au/Cu catalysts. n) Long‐term stability of Ag/Cu catalyst.

Self‐evolution tandem catalysts (Cu and M/Cu nanoparticles) were prepared by electrochemical treatment of CuO and M/CuO pre‐catalysts under cyclic voltammetry (CV) activation. In situ XRD patterns were utilized to investigate the phase transition of CuO and M/CuO during CV and ECR processes. As shown in Figures 3a–d, S17, S18, Cu and M/CuO catalysts transform into Cu and M/Cu after undergoing CV activation, and their phase structure remains stable in subsequent ECR tests at a constant potential. The morphology and composition of Cu and M/Cu after CV activation were characterized and shown in Figures S19–S26. Linear scanning voltammetry (LSV) was further carried out to check the oxidation state of Cu in Cu and M/Cu. However, there is no obvious reduction peak at ≈ –0.4 V, which is generally regarded as the reduction position for Cu^δ+^,^[^ ^44^ ^]^ indicating the valence state of Cu in self‐evolution Cu and M/Cu catalysts is close to 0 (Figure S27). We constructed in situ XAFS analyses of the Cu K‐edge to study the changes in coordination number of Cu during the real ECR process. As illustrated in Figures 3e‐h, S28‐S32, Tables S5–S8, the average coordination numbers of Cu fluctuate between 10.6 and 11.3 for Cu and M/Cu catalysts, indicating that the loading of Ag, Pd, and Au tandem elements have little influence on the coordination numbers of self‐evolved Cu, Ag/Cu, Pd/Cu, and Au/Cu catalysts.

To investigate the effect of different interfacial electric fields on catalysts’ activity, the ECR tests were carried out in a flow cell at constant potentials in 1 M KOH electrolyte. Figures 3i–l show the product distributions of Cu and M/Cu catalysts at different potentials. The Faraday efficiency of the CO product was extracted and plotted in Figure 3m. The results show M/Cu shows a similar and high FE_CO_ of ∼30% at a low potential of –1.2 V (versus RHE), while Cu only displays a FE_CO_ of 16.1%. The results imply the positive effect of tandem elements on producing CO. When the potential decreases from –1.2 to –2.0 V (versus RHE), Cu and M/Cu (M = Ag, Pd) have a sharp decline in selectivity for CO (FE_CO_ = ∼5%), whereas Au/Cu still has a high FE_CO_ of 20.1%. The CO selectivity of Au/Cu is significantly higher than that of Ag/Cu and Pd/Cu, mainly at the potentials of –1.6 V and –1.8 V (versus RHE). To investigate the influence of morphology on ECR, we conducted the double‐layer capacitance (C_dl_) tests for estimating the electrochemical active surface areas (ECSA) of M/Cu catalysts (Figures S33–S35). As shown in Figure S36, the partial current density of CO (j_CO_) before and after normalized by ECSA have similar trends of change, indicating the variations of CO selectivity over M/Cu tandem catalysts are independent of the morphology.^[^ ^45^ ^]^

To explore the formation ability of *CO on M/Cu tandem catalysts, we selected CO product at a potential of –1.2 V (versus RHE) under which M/Cu show high selectivity of CO. Figure S37 illustrates that Ag/Cu, Pd/Cu, and Au/Cu catalysts have similar j_CO_ and partial C_dl_ of CO at a potential of –1.2 V. Then, we constructed six calculation models to study the reaction energy barriers for *CO_2_ to *CO under 0.3 eV Å^−1^ electric field intensity (Figure S38. Tables S9–S12). As shown in Figure S39, Ag, Pd, and Au models have relatively low reaction energy barriers, and Au (0.70 eV) > Ag (0.42 eV) > Pd (0.34 eV). However, after compositing Cu, M/Cu have increased reaction energy barriers and the gaps among M/Cu tandem catalysts have been largely narrowed. The above results indicate that *CO formation ability over tandem element sites might be approximately similar for Ag/Cu, Pd/Cu, and Au/Cu catalysts. The highest CO selectivity for Au/Cu may be related to the weak adsorption capacity of *CO over Au/Cu heterostructures, as we predicted by DFT calculations (Figures 1b,c), resulting in the desorption and production of CO. As summarized in Figure S40, Ag/Cu exhibits the highest FE_C2+_ of 89.2% at –1.8 V (versus RHE) with a partial current density (j_c2+_) of 553.9 mA cm^−2^, which not only exceeds Cu and M/Cu (M = Au, Pd) in this work but also is comparable to the recently reported advanced catalysts (Table S13). The reason for the different catalytic performance of Cu and M/Cu will be further discussed in later sections.

At last, the stability of the optimal Ag/Cu catalyst was evaluated at a constant potential of –1.8 V (versus RHE) in a flow cell (Figure 3n). After 10 h, the FE_C2+_ of Ag/Cu remains 68.4%, and the current density decays from 615.3 mA cm^−2^ in the beginning 20 min to 407.5 mA cm^−2^ (Figure S41). The structure and composition of Ag/Cu catalyst were detected by SEM, TEM, elemental mappings, and XPS spectra after stability test (Figures S42–S45), indicating its relatively structural and compositional stability.

### Intrinsic Property Analysis and Catalytic Mechanism Study

**Figure 4:**
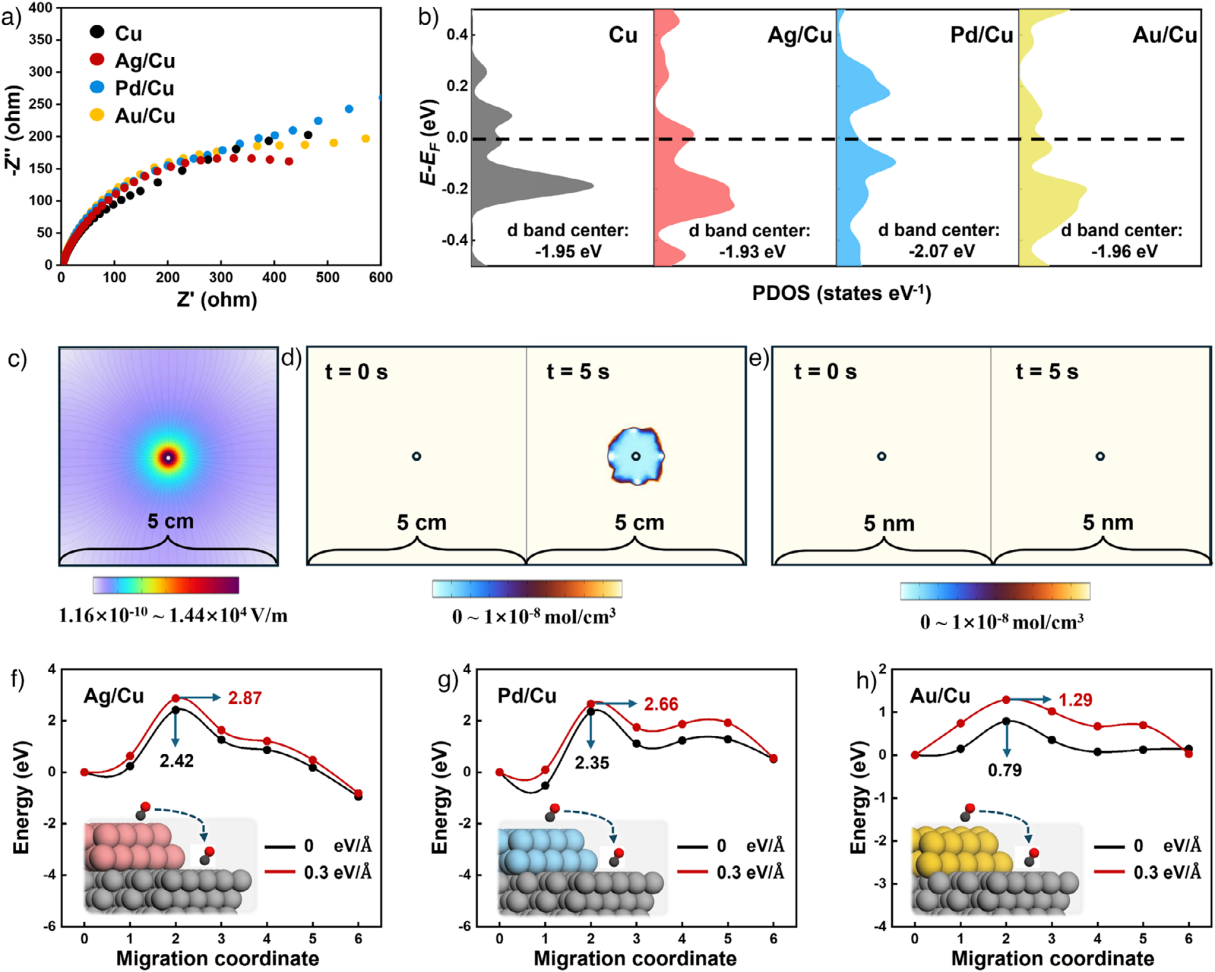
Electrochemical tests and investigations on the mechanism of Cu, Ag/Cu, Pd/Cu, and Au/Cu catalysts. a) Nyquist plots at open circuit potential, b) PDOS of the Cu 3d orbital of Cu, Ag/Cu, Pd/Cu, and Au/Cu catalysts. c) Electric field distribution for the 5 cm model. d) 5 cm model and e) 5 nm model of *CO concentration distribution at the 0‐s and 5‐s moment. The internal migration energy barrier of *CO under 0 and 0.3 eV Å^−1^ electric field intensities of f) Ag/Cu, g) Pd/Cu, and h) Au/Cu calculation models performance of Cu, Ag/Cu, Pd/Cu, and Au/Cu catalysts at different potentials.

Encouraged by the various electrochemical performance after combining the secondary tandem elements in Cu catalyst, we attempted to figure out the key properties of M/Cu that influence the electroreduction of CO_2_. Electrochemical impedance spectroscopy (EIS) tests and projected density of states (PDOS) of the Cu 3d orbital were conducted to understand the overall electron transfer ability of Cu and M/Cu catalysts. The results show that Cu and M/Cu have analogous resistance (Figure 4a) and similar electronic occupancy states near the Fermi level (Figure 4b). The above results indicate that the introduction of tandem elements hardly enhances or weakens the electron transfer ability of the Cu catalyst.^[^ ^46^ ^]^ We also calculated the d‐band center of Cu as the active site for Cu and M/Cu. It can be observed from Figure 4b that the energy of Ag/Cu is closer to the Fermi level, indicating that an enhanced adsorption of intermediates occurs over the Ag/Cu catalyst.^[^ ^47^ ^]^ On the contrary, the d‐band center of Pd/Cu is farthest from the Fermi level compared to the four calculation models.

In addition to the *CO adsorption over the tandem catalyst will be influenced by the electric field, as indicated by DFT calculations (Figures 1b,c), the migration pathway of the *CO over the tandem catalyst also plays a role in modulating the C–C coupling and target chemicals. According to the previous reports,^[^ ^48^ ^]^ *CO migration over tandem catalysts might first go through desorption into the electrolyte and then re‐adsorption onto Cu sites (external migration) or directly migrate from tandem elements to Cu sites (internal migration). Therefore, COMSOL multiphysics simulations were performed to explore the influence of electric field on the “external migration” of *CO. We constructed two types of geometric models with similar CO concentration, as depicted in Figure 4c (5 × 5 cm) and Figure S46 (5 × 5 nm). Then, similar electric fields are applied in two models with gradually decreased intensity from the center to the border.

**Figure 5:**
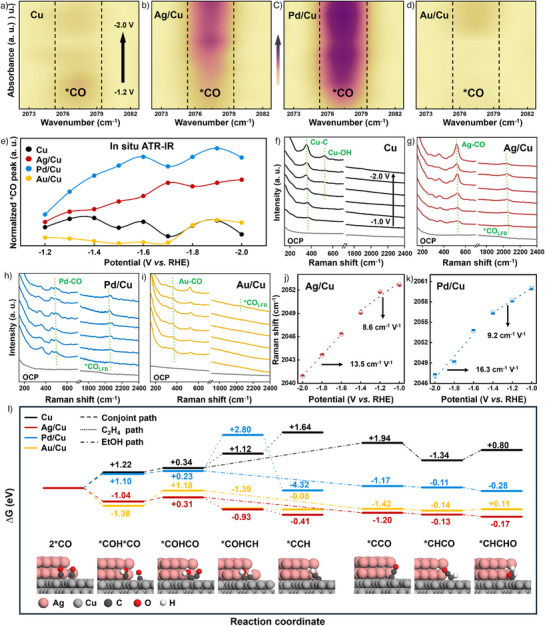
In situ characterizations and the Gibbs free energy of Cu, Ag/Cu, Pd/Cu, and Au/Cu catalysts. a)—d) In situ ATR‐IR spectra (from –1.2 to –2.0 V), e) normalized *CO peak area, and f)–i) in situ Raman spectra (from –1.0 to –2.0 V) of Cu, Ag/Cu, Pd/Cu, and Au/Cu catalysts. j) and k) Plot of Raman peak shift of *CO_LFB_ stretching versus potential of Ag/Cu and Pd/Cu catalysts. l) The Gibbs free energy diagrams for C_2_H_4_ and EtOH production over Cu, Ag/Cu, Pd/Cu, and Au/Cu models (2*CO, *COH*CO, *COHCO, *COHCH, *CCH, *CCO, *CHCO, and *CHCHO represent adsorbed species).

For the centimeter‐scale model (Figure 4d), CO is distributed uniformly when *t* = 0 s. After 5 s, the CO concentration is lower in the center because of the faster migration of CO under a stronger electric field. However, for the nanometer‐scale model (Figure 4e), the CO remained uniformly distributed after applying an electric field within 5 s, indicating that the stronger electric field has a relatively minor impact on the external migration of CO at the nanoscale. We further use a high‐precision display range to detect changes in CO concentration after 5 s for “5 × 5 nm” model (Figure S47), and the results were consistent with Figure 4e. Therefore, the influence of the electric field on the “external migration” behavior of *CO could be disregarded. Subsequently, we investigated the effect of the electric field on the “internal migration” of *CO by calculating the *CO migration energy barrier. As illustrated in Figures 4f–h, when the electric field increases to 0.3 eV Å^−1^, the migration energy barriers of *CO increase to different extents in all three M/Cu models. The result indicates that enhancing the electric field intensity can hinder the internal migration of *CO in tandem catalysts. Based on our experimental data (Figure S40), the enhanced interfacial electric field increases the adsorption capacity of *CO while also hindering its internal migration, but the efficiency of C_2+_ of the three catalysts is also increased. This implied that the adsorption capacity of *CO at Cu sites dominates C–C coupling compared to internal migration in tandem catalysts. We infer that tandem catalysts have provided a sufficient microenvironment for *CO enrichment, and compared to the migration ability of *CO, the adsorption capacity of *CO is the key factor dominant in C–C coupling. Examples of Ag/Cu and Au/Cu catalysts can also better prove the above viewpoint. Although Au/Cu has a lower migration energy barrier compared to Ag/Cu, its *CO adsorption capacity is poor, causing *CO escape and hindering C–C coupling. The overall results further verify that *CO adsorption has a more important impact than *CO migration on the selectivity of multicarbon products in tandem catalysts.

Furthermore, in situ ATR‐IR was operated to explore the relationship between the interfacial electric field intensity and *CO adsorption for Cu and M/Cu catalysts. As illustrated in Figures 5a–d and S48–S52, both Ag/Cu and Pd/Cu were detected to have strong *CO intermediate signals at 2078 cm^−1^.^[^ ^49^ ^]^ On the contrary, the *CO intermediate signals of Cu and Au/Cu were weak. Although the adsorption energy of *CO on Cu, Ag/Cu, and Pd/Cu is similar, Ag/Cu and Pd/Cu show more obvious *CO signals than Cu due to the spillover of CO brought about by the Ag and Pd elements, which resulting in an increased *CO coverage over tandem catalysts. Notably, the low *CO intermediate coverage of Au/Cu may be due to the weak adsorption capacity of *CO as discussed in DFT calculations, which leads to the *CO escape from Cu sites. Higher FE_CO_ was also detected in the Au/Cu catalyst product distribution at different potentials (Figure 3m). We normalized the peak area of the *CO intermediate (Figure 5e) and found that the intensity of the *CO intermediate signal gradually increases as the interfacial electric field increases for Ag/Cu and Pd/Cu. Similarly, Cu and Au/Cu have a lower intensity of *CO intermediate compared to Ag/Cu and Pd/Cu. We conducted in situ Raman spectroscopy to quantitatively analyze the relationship between *CO adsorption and interfacial electric field intensities (Figure S53). As illustrated in Figures 5f–i, peaks at 350–360, 520–530, 490–500, and 360–370 cm^−1^ are attributed to Cu─C stretching, Ag─CO stretching, Pd─CO stretching, and Au─CO stretching, respectively.^[^ ^35^, ^46^ ^]^ Peaks at 2040–2060 and 2080–2100 cm^−1^ are corresponded to *CO stretching at the low frequency band (LFB) and *CO stretching at the high frequency band (HFB), respectively.^[^ ^50^ ^]^ In Ag/Cu and Pd/Cu catalysts, the signals of *CO stretching are more obvious, which are consistent with in situ ATR‐IR results (Figures 5a–d). As depicted in Figures 5j,k, the Stark tuning slope of *CO_LFB_ stretching at low potentials is smaller than that at high potentials for both Ag/Cu and Pd/Cu catalysts,^[^ ^51^ ^]^ indicating that as the interfacial electric field intensity increases, the adsorption capacity of *CO gradually enhances to a certain extent. The above experimental results are consistent with our calculations, indicating that the enhanced interfacial electric field intensities increase the adsorption capacity and coverage of *CO, thus improving the selectivity of C_2+_ products.

Gibbs free energy was performed to investigate the reaction energy barriers for the transformation of various intermediates during the ECR process (Figures S54 and S55, Tables S14–S18). As illustrated in Figure 5l, catalysts composed of heterogeneous tandem elements have lower reaction energy barriers compared to the pure Cu catalyst. Among heterogeneous tandem elements, Au/Cu has a reaction energy barrier similar to Ag/Cu, but its weak adsorption of *CO may be unfavorable for subsequent C–C coupling. The ECR energy barrier of Pd/Cu is much higher than that of Ag/Cu and Au/Cu. This might be an important factor for the low ECR performance of the Pd/Cu catalyst. Although Pd/Cu generally has a relatively high reaction energy barrier, it is easy for promoting *COHCH→*CCH step. This result inspires us that we may be able to construct a multicomponent tandem catalyst to regulate each step of the reaction. Based on the above experimental results and DFT theoretical calculations, we have noticed that a strong interfacial electric field could enhance the adsorption of *CO on the tandem catalysts. In future research, it may provide guidance on constructing efficient catalysts through the regulation of interfacial electric fields and the selection of multicomponent tandem elements.

## Conclusion

In summary, we constructed four *CO adsorption models (Cu, Ag/Cu, Pd/Cu, and Au/Cu) under different interfacial electric field intensities by DFT calculations. The results indicate that with the increase of interfacial electric field intensities, the *CO adsorption capacity is enhanced to a certain extent. Thereunder, we designed three kinds of self‐evolution tandem catalysts (Ag/Cu, Pd/Cu, and Au/Cu catalysts) and performed ECR tests under constant potentials. The low C_2+_ selectivity of Pd/Cu is attributed to its high reaction energy barrier and low catalytic activity. The high CO selectivity of Au/Cu is owing to the weak adsorption capacity for *CO, which leads to the escape of the generated *CO. Ag/Cu catalyst exhibited a high FE_C2+_ of 89.2% and a partial current density (j_c2+_) of 553.9 mA cm^−2^ at –1.8 V (versus RHE) as a result of a low reaction energy barrier and moderate *CO adsorption capacity. COMSOL multiphysics simulations indicate that the effect of electric field on the external migration of *CO could be almost negligible at the nanoscale. DFT and experimental results indicate that compared to the internal migration of *CO, the adsorption capacity of *CO dominates the generation of multicarbon products through C–C coupling in tandem catalysts. In situ ATR‐IR and in situ Raman experimental results on the relationship between *CO and interfacial electric field intensities are similar to our calculation results. This work provides new ideas for the study of *CO adsorption capacity and migration in interfacial electric fields for tandem catalysts and also offers insights for the design of multicomponent tandem catalysts.

## Conflict of Interests

The authors declare no conflict of interest.

## Supporting information



Supporting Information

## Data Availability

The data that support the findings of this study are available on request from the corresponding author.
