# Supplementary material for: Behavior Regulation of *CO over Self‐Evolution Tandem Catalysts Under Tuned Interfacial Electric Field Boosts CO2 Electroreduction
Source: Angew Chem Int Ed Engl. 2025 Aug 19;64(40):e202511704. doi: 10.1002/anie.202511704 (PMC12462755; doi:10.1002/anie.202511704)
Supplement: Supplementary file 1 — Supporting Information [file ANIE-64-e202511704-s001.docx]

**Supporting Information**

**Behavior Regulation of *CO over Self-Evolution Tandem Catalysts Under Tuned Interfacial Electric Field Boosts CO_2_ Electroreduction**

*Zining Zhang^1^, Xinyan Ma^1^, Yang Song^1, 2^, Xue Yang^1,^ ^2^, Qi Fang^1^, Yusuke Yamauchi*^3, 4^, and Jing Tang*^1, 5^*

1. State Key Laboratory of Petroleum Molecular & Process Engineering, Shanghai Key Laboratory of Green Chemistry and Chemical Processes, School of Chemistry and Molecular Engineering, East China Normal University, Shanghai 200062, China.

2. State Key Laboratory of Petroleum Molecular & Process Engineering, Sinopec Research Institute of Petroleum Processing Co., LTD., Beijing 100083, China

3. Australian Institute for Bioengineering and Nanotechnology (AIBN), The University of Queensland, Brisbane QLD 4072, Australia

4. Department of Materials Process Engineering, Graduate School of Engineering, Nagoya University, Nagoya University, Nagoya, Aichi 464-8603, Japan

5. Institute of Eco-Chongming, Shanghai 202162, China

Emails: [y.yamauchi@uq.edu.au](mailto:y.yamauchi@uq.edu.au); jingtang@chem.ecnu.edu.cn

**Experimental Section**

**Chemicals.**

Copper chloride dihydrate (CuCl_2_∙2H_2_O), sodium hydroxide (NaOH), L-ascorbic acid, silver nitrate (AgNO_3_), palladium (Ⅱ) nitrate dihydrate (Pd (NO_3_)_2_·2H_2_O), tetrachloroauric (Ⅲ) acid tetrahydrate (HAuCl_4_·4H_2_O), potassium bicarbonate (KHCO_3_), potassium hydroxide (KOH), isopropanol, deuterium oxide (D_2_O) and dimethyl sulfoxide (DMSO) were purchased from Aladdin Industrial Inc. (Shanghai, China). CO_2_ with a purity of 99.999% was provided by Shanghai Chemistry Industrial Zone Pujiang Special Type Gas Co., Ltd.

**Synthesis of CuO.**

0.51 g CuCl_2_∙2H_2_O and 4.80 g NaOH were dissolved in 60 mL deionized water. The mixed solution was heated at 100 ℃ for 12 h in a Teflon reactor. After centrifugation of the solution, CuO powder was obtained by vacuum drying at 60 ℃ for 10 h.

**Synthesis of Ag/CuO, Pd/CuO, and Au/CuO pre-catalysts.**

Dissolve 0.05 g CuO powder in 45 mL deionized water, and add 0.18 g of L-ascorbic acid during stirring to prepare solution A. Dissolve 0.03 g AgNO_3_, 0.04 g Pd (NO_3_)_2_·2H_2_O, and 0.07 g HAuCl_4_·4H_2_O in 15 mL deionized water to prepare solution B, C, and D, respectively. Drop solution B, C, and D into solution A under continuous stirring, respectively, then perform centrifugation. After vacuum drying at 60℃ for 12 h, Ag/CuO, Pd/CuO, and Au/CuO powders can be obtained.

**Synthesis of Cu, Ag/Cu, Pd/Cu, and Au/Cu.**

10 mg of CuO, Ag/CuO, Pd/CuO, and Au/CuO powders were dispersed into 600 μL of isopropanol with 30 μL of Nafion and 10 μL of PTFE solution, sonicated for 30 minutes to obtain a homogenous catalyst ink, subsequently, the ink was loaded onto gas diffusion layer (GDL, 1.5 cm × 3 cm) and dried under an infrared lamp to obtain the gas diffusion electrodes (GDEs). Cu, Ag/Cu, Pd/Cu, and Au/Cu were obtained through the electrochemical treatment of GDEs by cyclic voltammetry (0.6 to −2.0 V vs RHE) for 30 circles in 1 M KHCO_3_ aqueous solution in a flow cell.

**Characterization.**

The phase structures were measured by X-ray diffraction (XRD) patterns on a Rigaku Ultima IV X-ray diffractometer using Cu Kα radiation at 35 kV and 25 mA (λ = 1.5405 Å). The surface components were analyzed by X-ray photoelectron spectroscopy (XPS, ESCA-LAB250Xi) on an Al Kα radiation. The micro morphologies and elements were tested by scanning electron microscopy (SEM, Zeiss Gemini 450), TEM (JEOL JEM-F200). The metal content in the catalysts was determined by inductively coupled plasma optical emission spectroscopy (ICP-OES, Optima 8300, Perkin-Elmer). The X-ray absorption spectroscopy (XAS) tests were carried out by Super Photon ring-8 at Harima Science Garden City, Hyogo, Japan. In situ X-ray diffraction (XRD) were recorded on a DEEP-XRD-R1 diffractometer (Beijing SciStar Technology Co., Ltd.) using Cu Kα radiation (λ = 1.5418 Å) operated at 40 kV and 30 mA. In situ X-ray absorption fine structure (XAFS) analyses of the Cu K-edge were conducted utilizing a commercial Laboratory-Based XAFS spectrometer (RapidXAFS 2M, Anhui Absorption Spectroscopy Analysis Instrument Co., Ltd.). In situ attenuated total reflection-infrared (ATR-IR) spectroscopy was carried out on a Thermo Scientific Nicolet 6700 ATR-IR spectrometer equipped with a liquid N_2_-cooled system MCT detector. In situ Raman spectra were collected on a confocal Raman microscope (Thermo Scientific DXR2) with a 785 nm solid laser as an excitation source.

**Electrochemical In Situ XRD Analysis.**

In situ X-ray diffraction (XRD) were recorded on a DEEP-XRD-R1 diffractometer (Beijing SciStar Technology Co., Ltd.) using Cu Kα radiation (λ = 1.5418 Å) operated at 40 kV and 30 mA. The in-situ data were collected by an electrochemical in situ X-ray diffraction reaction cell (EC-XRD) over a 2θ range of 5° to 90° with a step size of 0.17° and a counting time of 1 s per step. We utilized Ag/AgCl as the reference and 1 M KHCO_3_ as the electrolyte. The electrolyte was bubbled with CO_2_ for 20 minutes before the experiment and continuously introduce CO_2_ during the testing process. The test potential is -1.8 V (vs. RHE throughout the work).

**Electrochemical In Situ EXAFS Analysis.**

Both static and in situ X-ray absorption fine structure (XAFS) analyses of the Cu K-edge were conducted utilizing a commercial Laboratory-Based XAFS spectrometer (RapidXAFS 2M, Anhui Absorption Spectroscopy Analysis Instrument Co., Ltd.). X-rays were generated via a Mo target X-ray source operated at 20 kV and 20 mA. A Si (553) spherically bent crystal analyzer (SBCA) with a radius of curvature of 500 mm served as the monochromator, thereby ensuring a diffraction geometry approaching a 90-degree backscatter angle at the absorption edge. After monochromatization, the X-rays pass through the sample and were collected using a high-energy-resolution silicon drift detector (SDD) to obtain the X-ray intensity. The XAFS data were acquired in transmission mode. During the XAFS measurements, the position of the absorption edge (E0) was calibrated using a standard Cu foil sample, and all data collection occurred within a single time period. Ag/AgCl was used as the reference. The test potential is -1.8 V and the workstation used for electrocatalytic reactions is CHI 630 instrument (Shanghai, Chenhua, China). Continuously introduce CO_2_ during the testing process.

**Electrochemical In Situ ATR-IR Analysis.**

In situ attenuated total reflection-infrared (ATR-IR) spectroscopy was carried out on a Thermo Scientific Nicolet 6700 ATR-IR spectrometer equipped with a liquid N_2_-cooled system MCT detector. In a germanium crystal in-situ FT-IR cell, we pressed Cu, Ag/Cu, Pd/Cu, and Au/Cu electrodes on the germanium crystal by a glassy carbon electrode vertically. We utilized Hg/HgO as the reference and platinum mesh as the counter electrode. 50 mL 1 M KOH solution was used as the electrolyte which was bubbled with CO_2_ for 30 minutes before the experiment and continuously introduce CO_2_ during the testing process. The workstation used for electrocatalytic reactions is CHI 630 instrument (Shanghai, Chenhua, China).

**Electrochemical In Situ Raman Analysis.**

In situ Raman spectra were collected on a confocal Raman microscope (Thermo Scientific DXR2) with a 785 nm solid laser as an excitation source. The measurements were performed in a custom-built flow cell with a similar configuration as the flow cell reactor. We utilized Ag/AgCl as the reference and carbon rod as the counter electrode. 35 mL 1M KHCO_3_ solution was used as the electrolyte in a flow cell. The test potential is from -1.0 to -1.8 V. Continuously introduce CO_2_ gas during the testing process. The workstation used for electrocatalytic reactions is CHI 630 instrument (Shanghai, Chenhua, China).

**Electrochemical properties of different electrodes.**

The EIS measurements were carried out at open circuit potential with an amplitude of 5 mV and a frequency from 10^−2^ to 10^5^ Hz in flow cells with 1 M KOH electrolyte. The electrochemical active surface area (ECSA) of various electrodes was calculated through the electric double-layer capacitance (C_dl_) in a flow cell with 1 M KOH electrolyte. CV scanning was performed in the non-Faraday zone with different scan rates (10−100 mV s^-1^). The current density difference (Δj) between oxidation and reduction at different scan rates was calculated. The slope for the plot of Δj vs. scan rate was the C_dl_ value.

**Electrocatalysis Experiments.**

For a flow cell test, CO_2_ electroreduction was conducted in a flow-cell (Gaossunion Co., Ltd.) using electrochemical workstation (CHI630E) equipped with a high-current amplifier (CHI680C). The flow-cell configuration consisted of a three-electrode system, and the as prepared GDEs, nickel foam and Ag/AgCl electrode were used as the working electrode, counter electrode and reference electrode, respectively. 1 M KOH for catholyte and anolyte were separated by an anion exchange membrane FUMA-FAA-3-PK-130. The electrolyte was circulated through the cathode and anode chambers at a rate of 30 mL min^−1^ using a peristaltic pump.

We used the following relation and compensated with the solution resistance.to convert all tested potentials to (reversible hydrogen electrode) RHE.

$$E_{RHE}=E_{Hg/HgO}+0.241+0.0592\times pH/E_{Ag/AgCl}+0.197+0.0592\times pH$$

where E_Hg/HgO_ and E_Ag/AgCl_ stand for the applied potential.

**Product Analysis.**

The controlled potential electrolysis was performed at each potential for 20 minutes, and the gaseous products were collected and detected by gas chromatography instrument (GC-2014, Shimadzu) with TCD and FID detectors. The liquid product of electrochemical tests was analyzed by nuclear magnetic resonance (NMR, Bruker Avance III HD500 spectrometer). DMSO was used as the internal standard with a chemical shift of 2.6 ppm. The ^1^H spectrum was measured with water suppression method. The Faradaic efficiency (FE) of each product was calculated as follows:

$$FE(\%)=\frac{n\times Z\times F}{Q}\times100\%$$

where n represents the total amount of specified products (in moles), Z represents the number of electrons involved in the electrode reaction, F is the Faraday constant (96485 C mol^−1^) and Q presents the amount of charge passed through the working electrode.

**Computational Methods.**

DFT calculations were applied by utilizing the Vienna Ab initio Simulation Package (VASP) with the projected augmented wave (PAW) method.^[1]^ We employed the generalized gradient approximation (GGA) of the Revised Perdew-Burke-Ernzerhof (r-PBE) exchange-correction functional and a cutoff energy of 400 eV.^[2]^ The van der Waals (vdW) interactions were taken into account by the empirical correlation scheme of DFT-D3.^[3]^ Geometry optimizations were performed with the force convergency smaller than 0.05 eV/Å. Monkhorst-Pack k-points of 2×1×1 was adopted for all the calculations.. The free energy changes (ΔG) of each reaction step during CO_2_ electroreduction were calculated by the computational hydrogen electrode (CHE) model. The Gibbs free energy was calculated by the following equation:

$$\Delta G=\Delta E+E_{ZPE}-T\Delta S$$

where ΔE is the enthalpy difference from DFT computations, ΔE_ZPE_ is the change in zero-point energy and ΔS is the temperature (298.15 K) and the entropy changes, which can be calculated from the vibrational frequencies.^[4]^

COMSOL multiphysics simulations were performed by constructing a two-dimensional finite element model to describe the external migration of *CO under different electric field intensities. We took two-dimensional cross-section of “5 cm × 5 cm” and “5 nm × 5 nm” as the computational domain. We applied the rare substance transfer module in COMSOL Multiphysics software for the finite element simulation to analyze the diffusion behavior of CO under different electric field intensities. The model of electric field is shown in Figure 4f and S27. The diffusion constant of CO was taken to be 1.00×10^-9^ m^2^/s and the initial concentration of CO was 1.4 mol/m^3^.

**Supporting Figures**


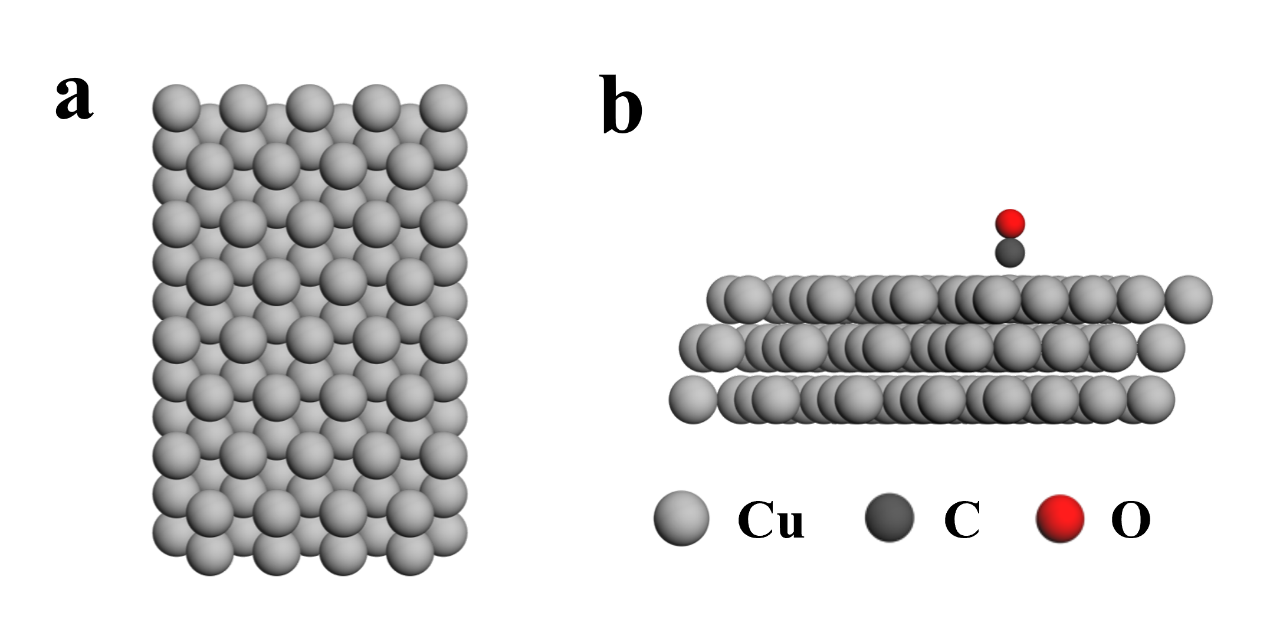


**Figure S1. (a)** Top view of the geometry of Cu (111) slab for DFT calculations. **(b)** The adsorption configuration of *CO for the Cu computation model (*CO represents adsorbed species).


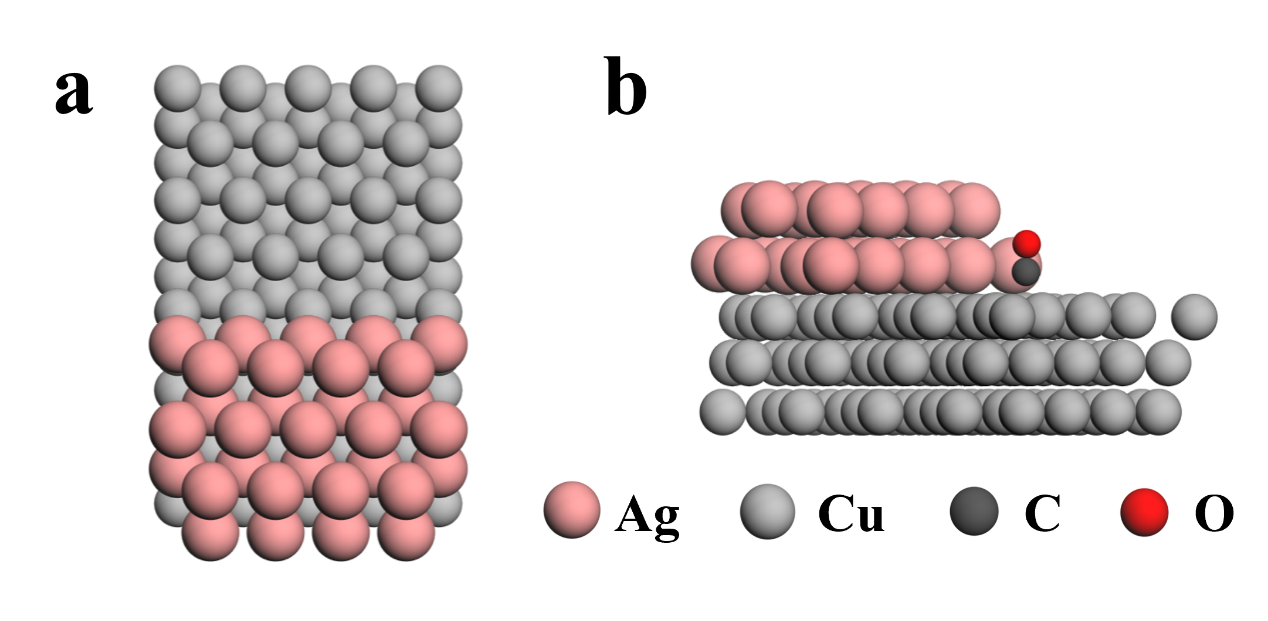


**Figure S2. (a)** Top view of geometry of Ag/Cu heterostructure slab for DFT calculations. **(b)** The adsorption configuration of *CO for the Ag/Cu computation model (*CO represents adsorbed species).


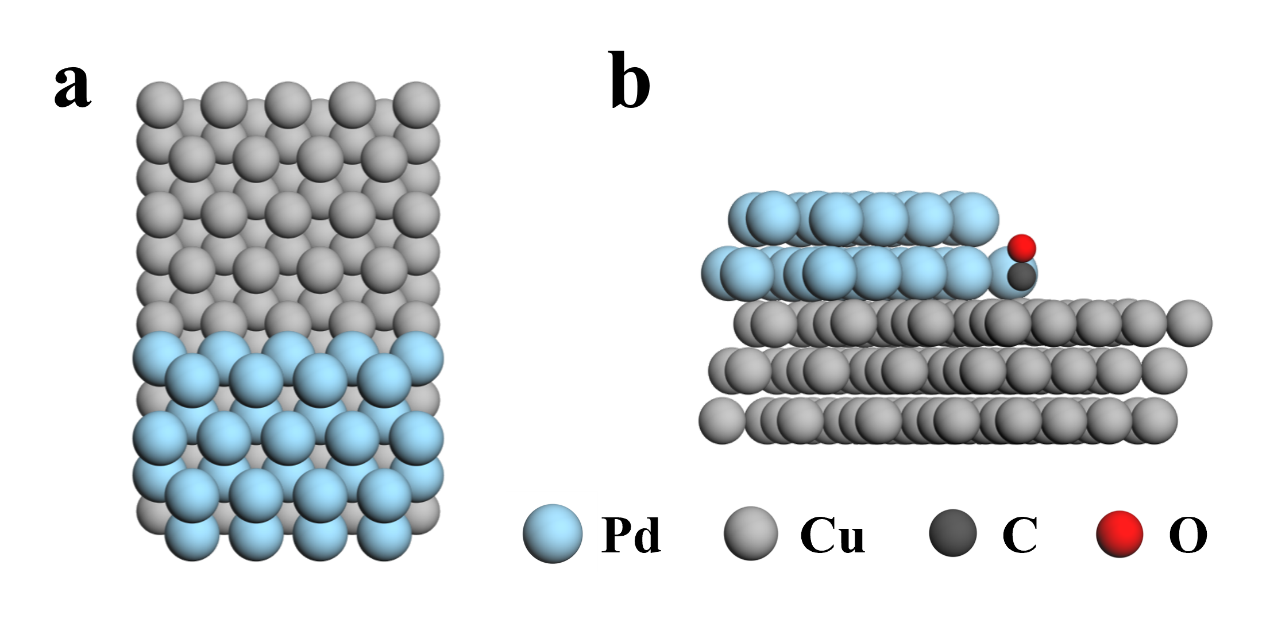


**Figure S3**. **(a)** Top view of geometry of Pd/Cu heterostructure slab for DFT calculations. **(b)** The adsorption configuration of *CO for the Pd/Cu computation model (*CO represents adsorbed species).


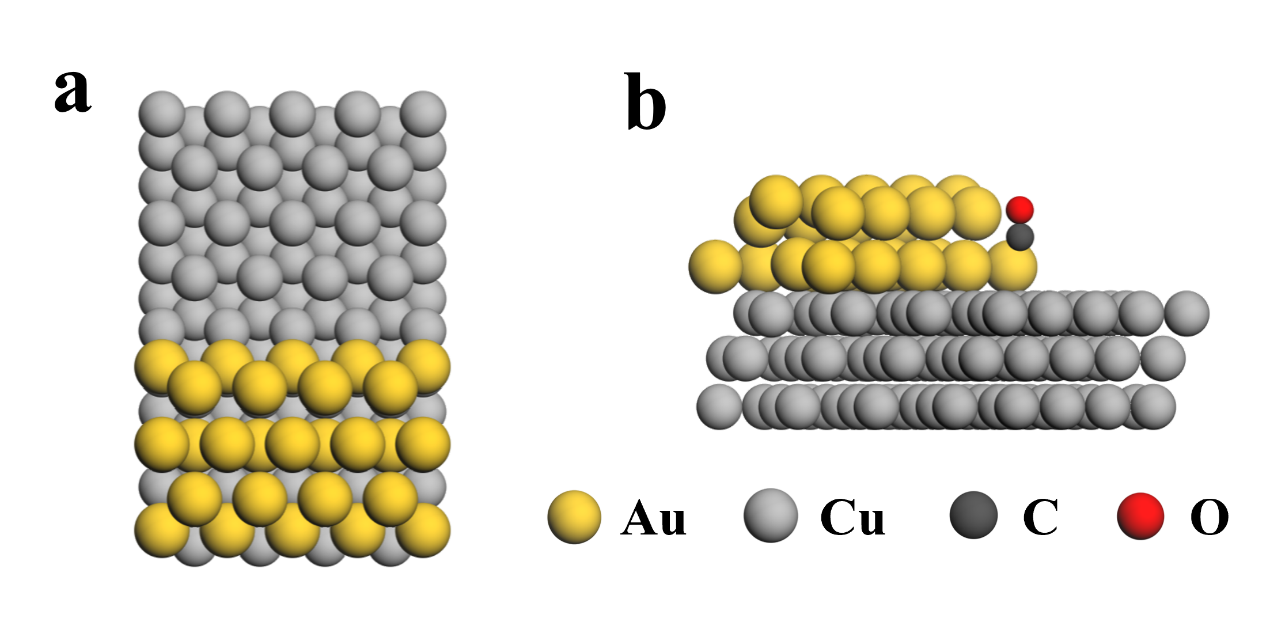


**Figure S4. (a)** Top view of the geometry of Au/Cu heterostructure slab for DFT calculations. **(b)** The adsorption configuration of *CO for the Au/Cu computation model (*CO represents adsorbed species).


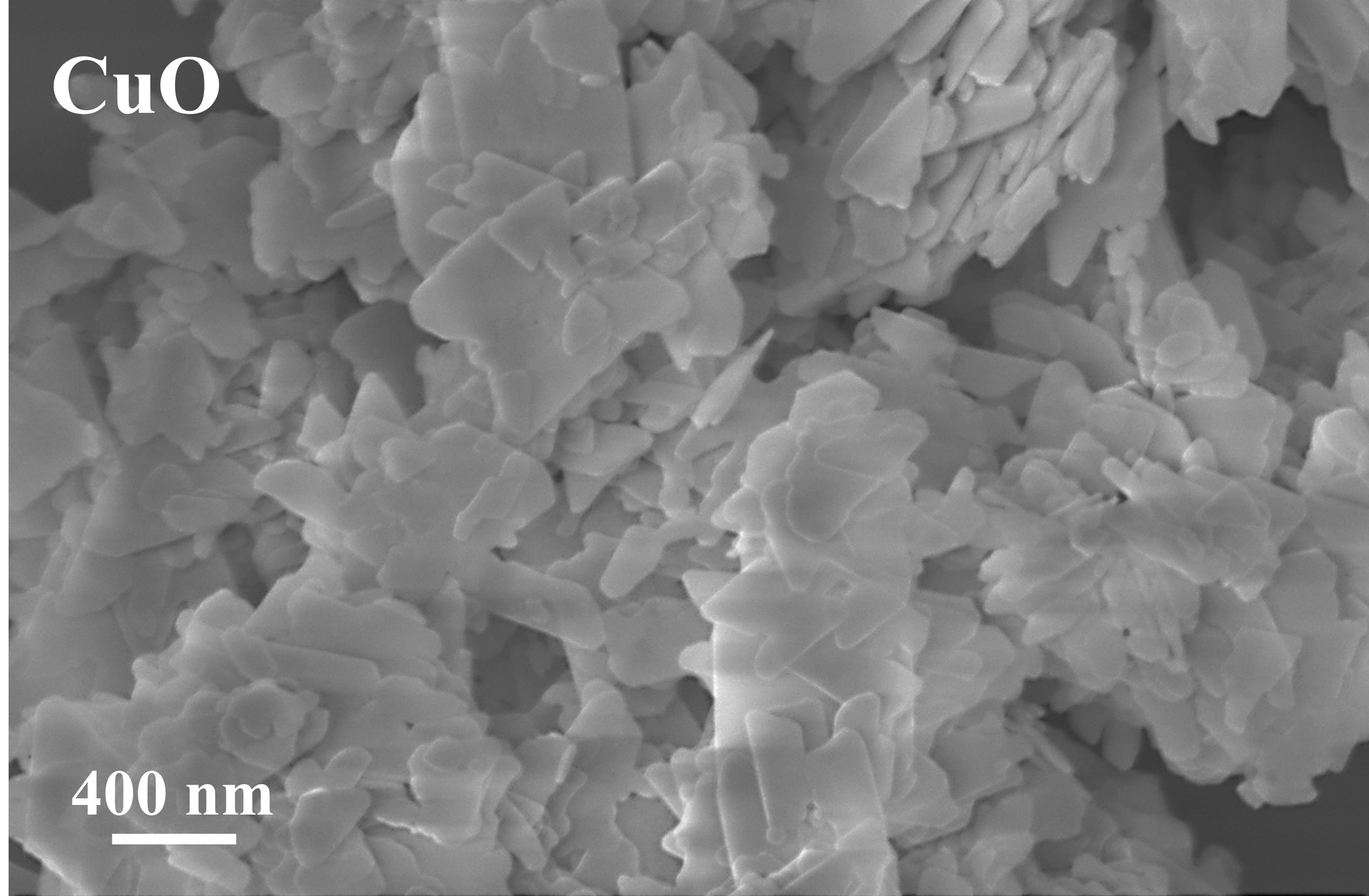


**Figure S5.** SEM image of CuO nanosheets.


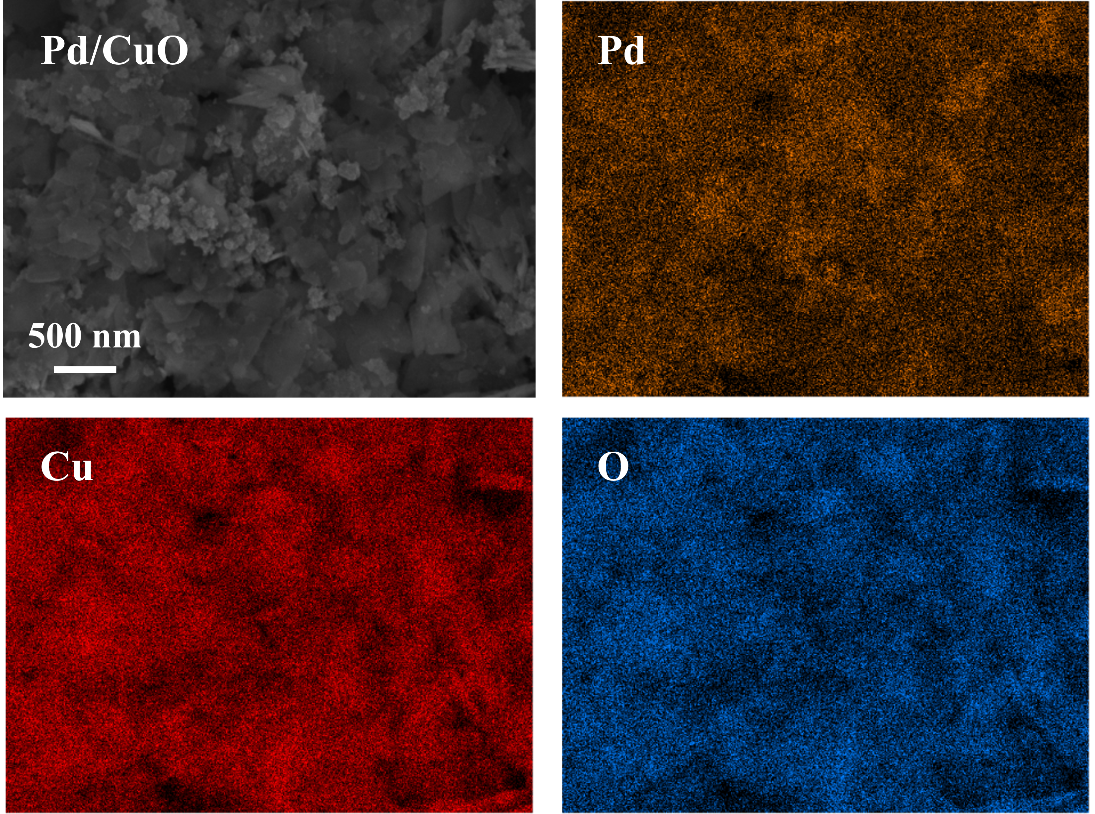


**Figure S6.** SEM image and elemental mappings of Pd/CuO.


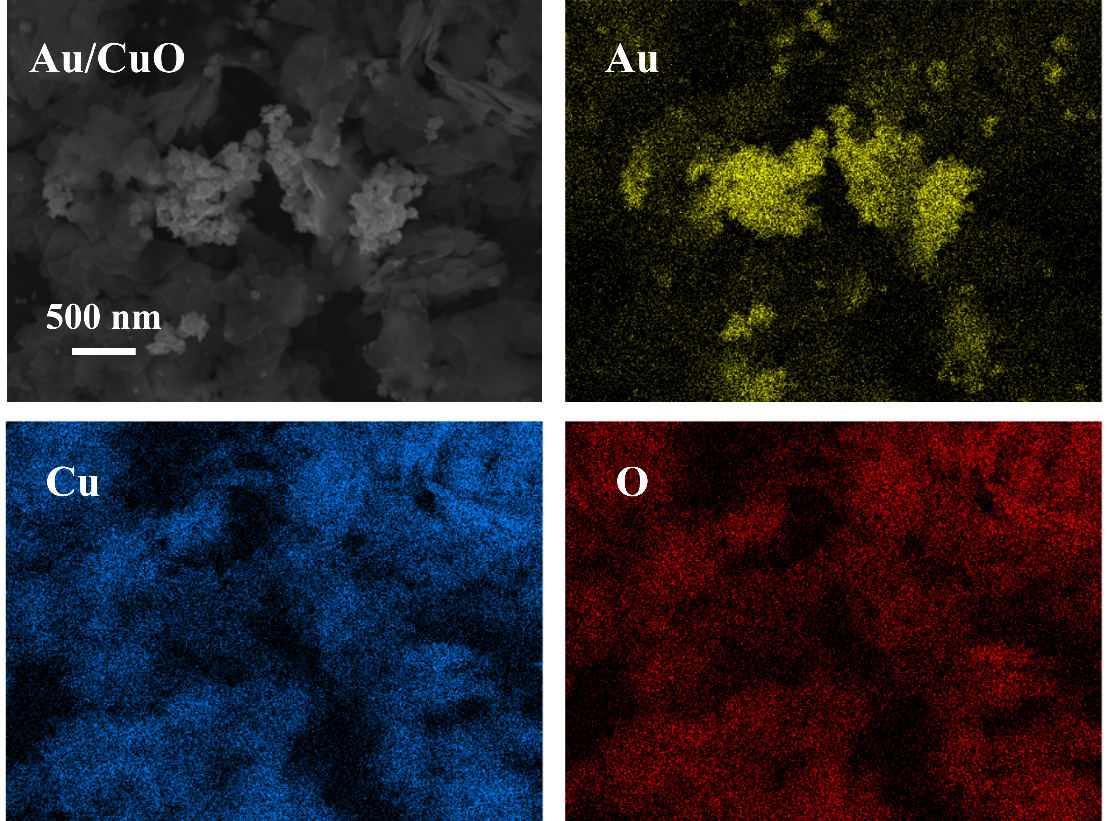


**Figure S7.** SEM image and elemental mappings of Au/CuO.


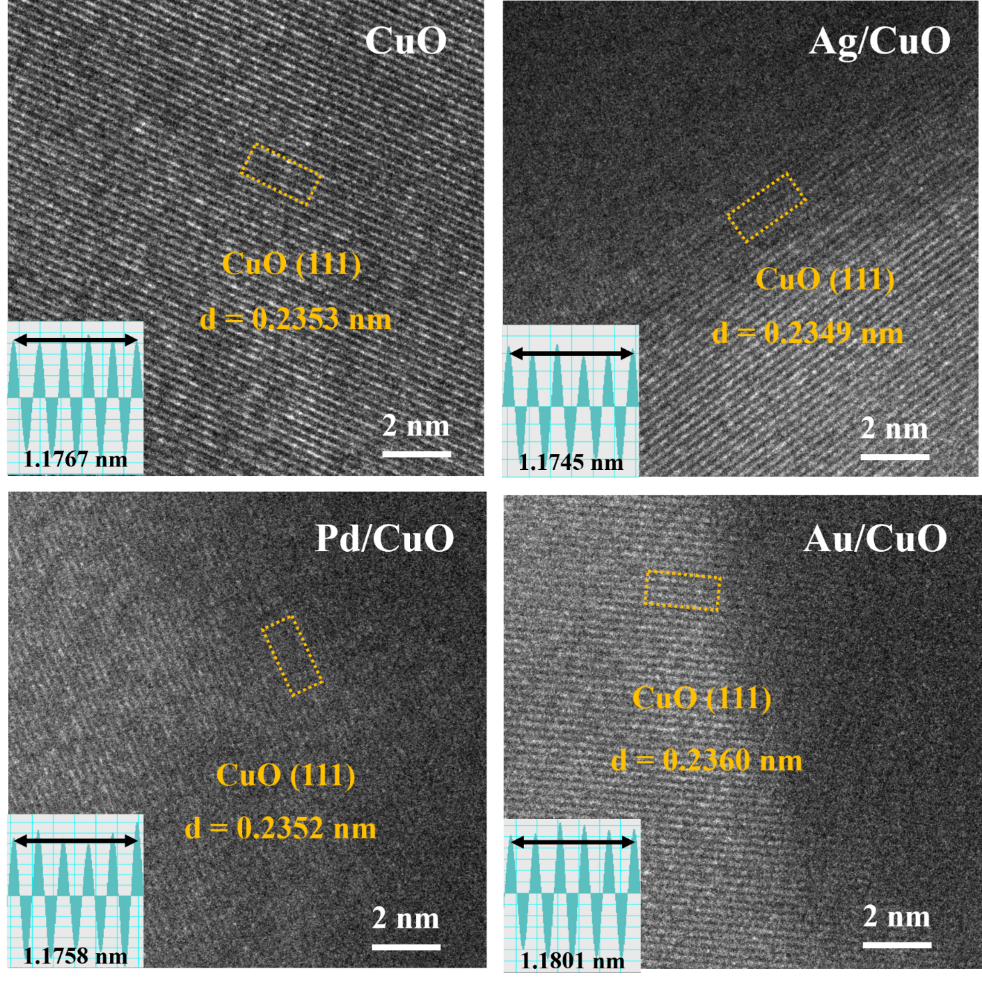


**Figure S8.** HRTEM images of CuO, Ag/CuO, Pd/CuO, and Au/CuO pre-catalysts.


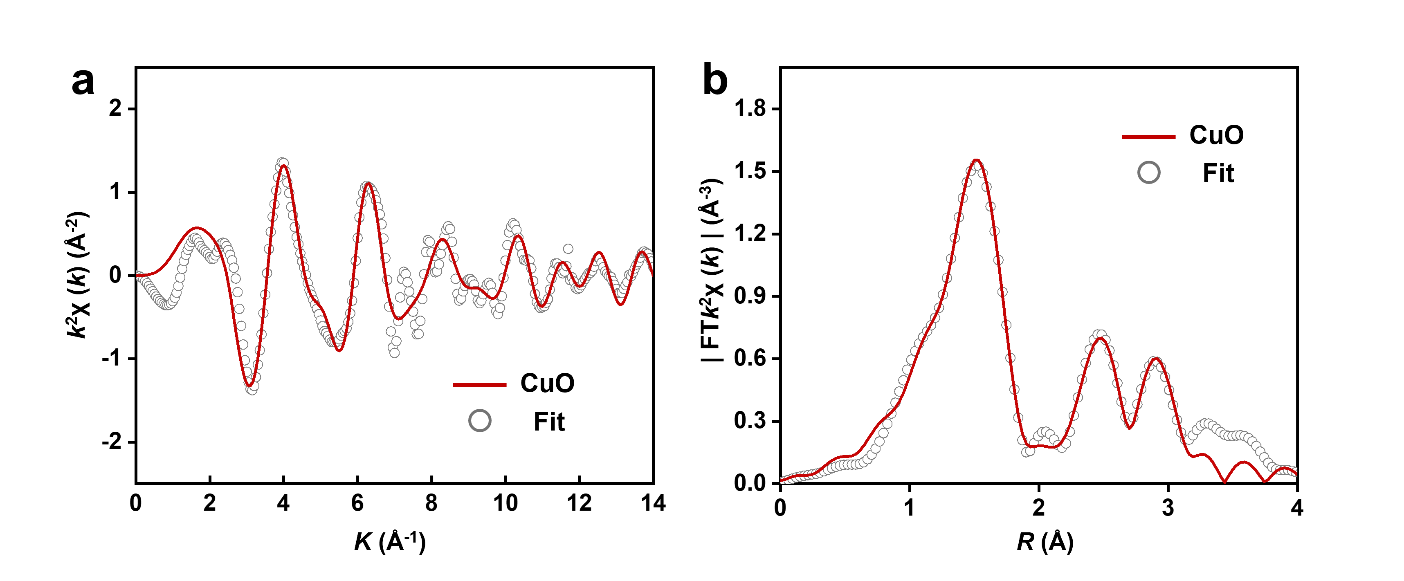


**Figure S9.** EXAFS fitting curves of CuO in the **(a)** k-space and **(b)** R-space.


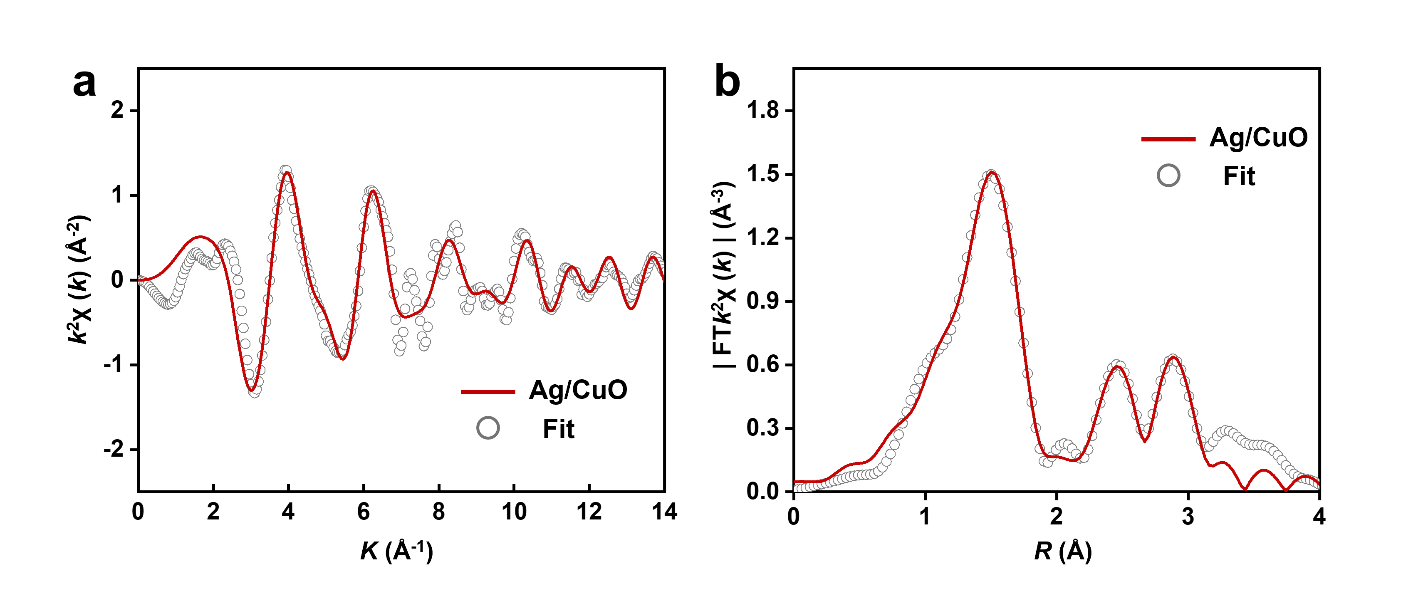


**Figure S10.** EXAFS fitting curves of Ag/CuO in the **(a)** k-space and **(b)** R-space.


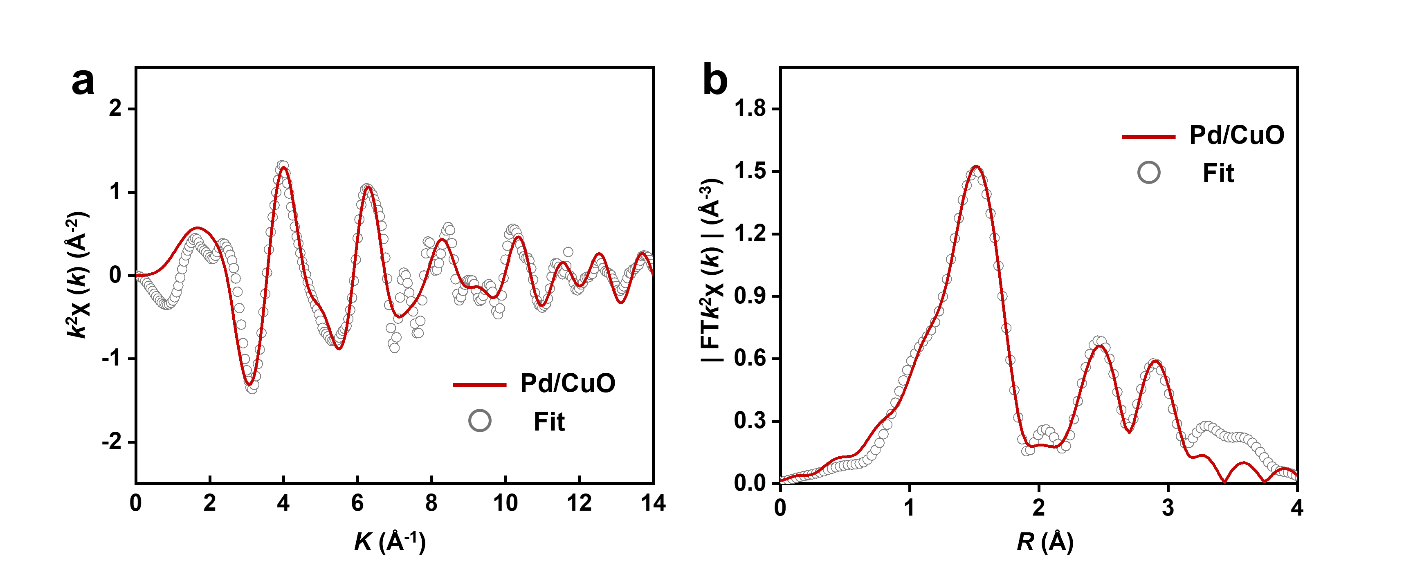


**Figure S11.** EXAFS fitting curves of Pd/CuO in the **(a)** k-space and **(b)** R-space.


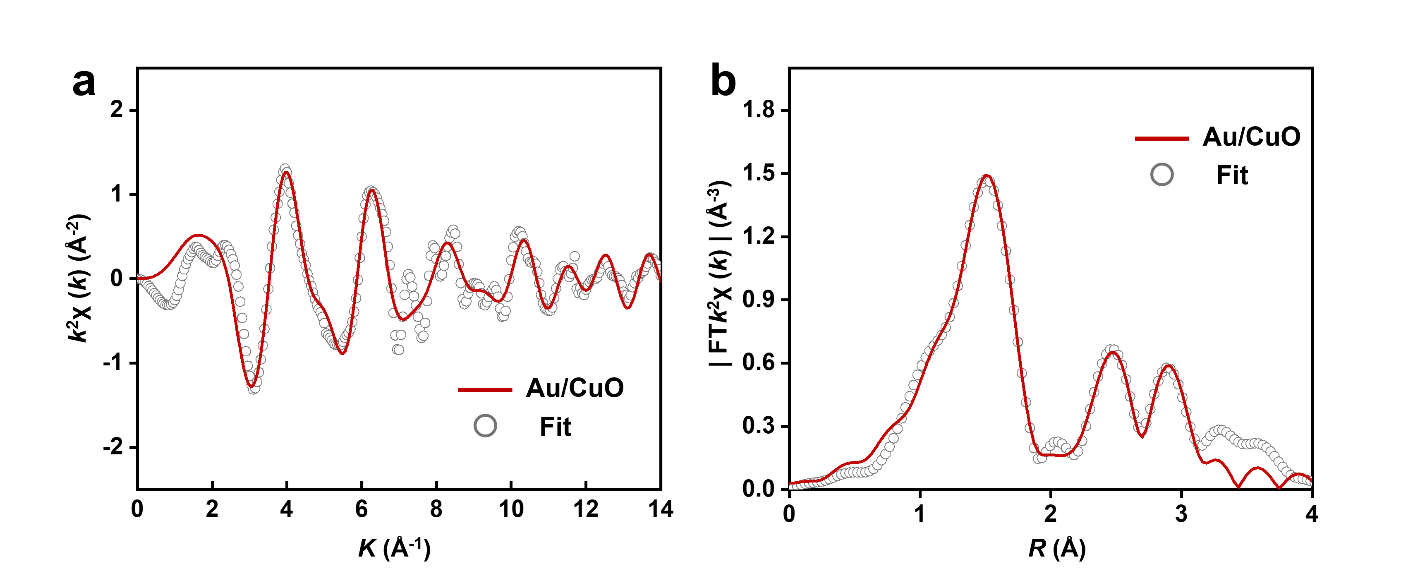


**Figure S12.** EXAFS fitting curves of Au/CuO in the **(a)** k-space and **(b)** R-space.


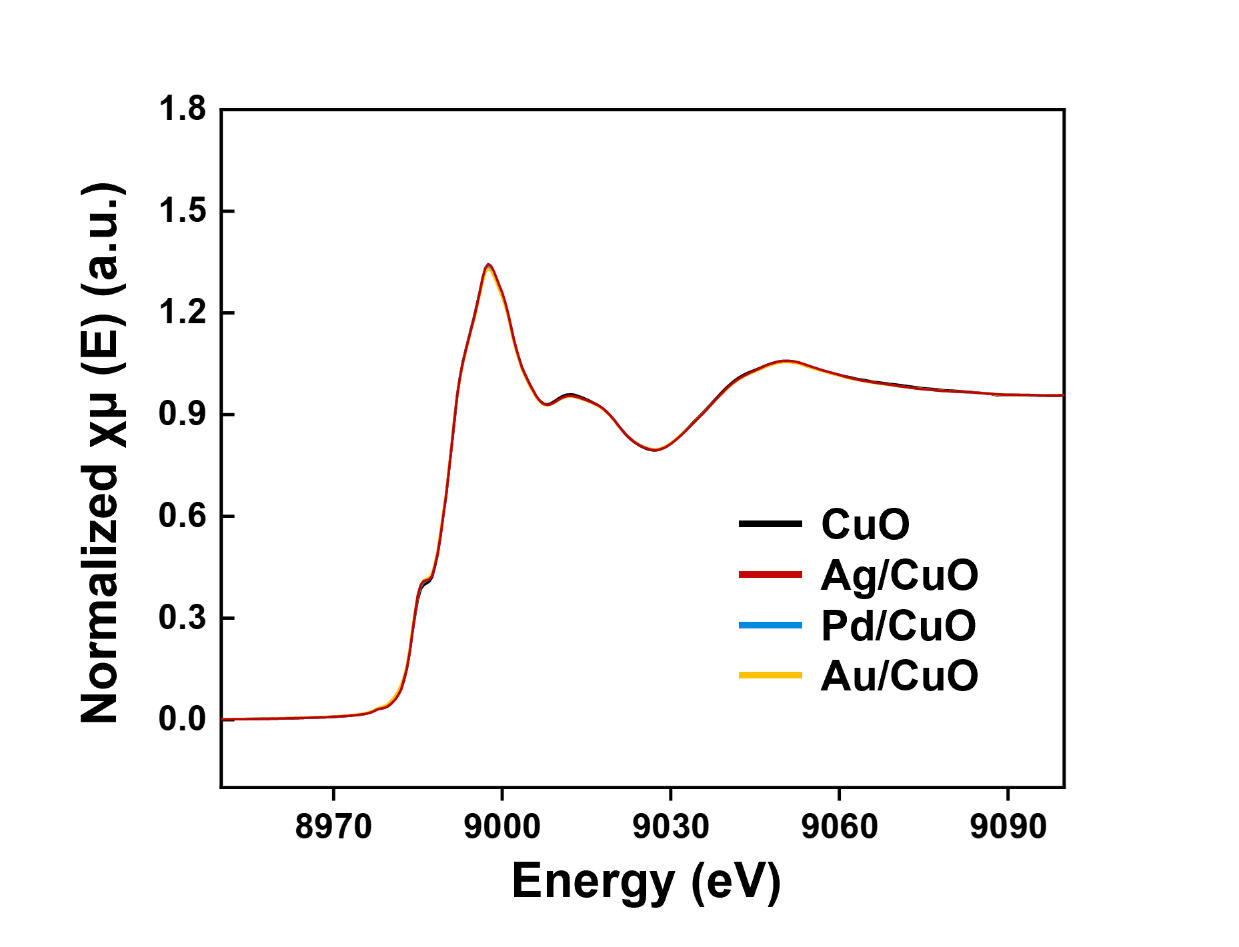


**Figure S13.** XANES spectra of CuO, Ag/CuO, Pd/CuO, and Au/CuO pre-catalysts.


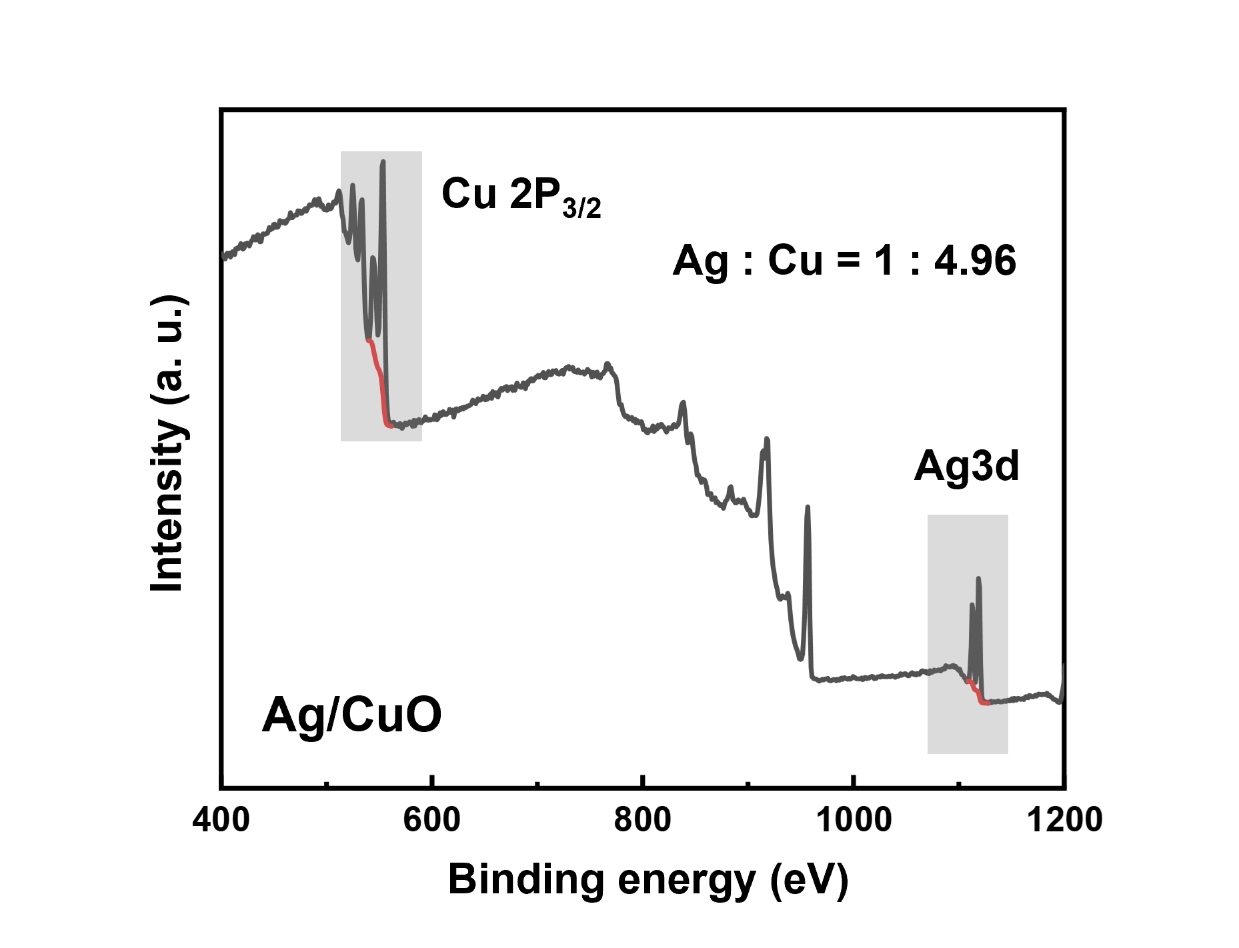


**Figure S14.** Survey XPS spectrum of Ag/CuO.


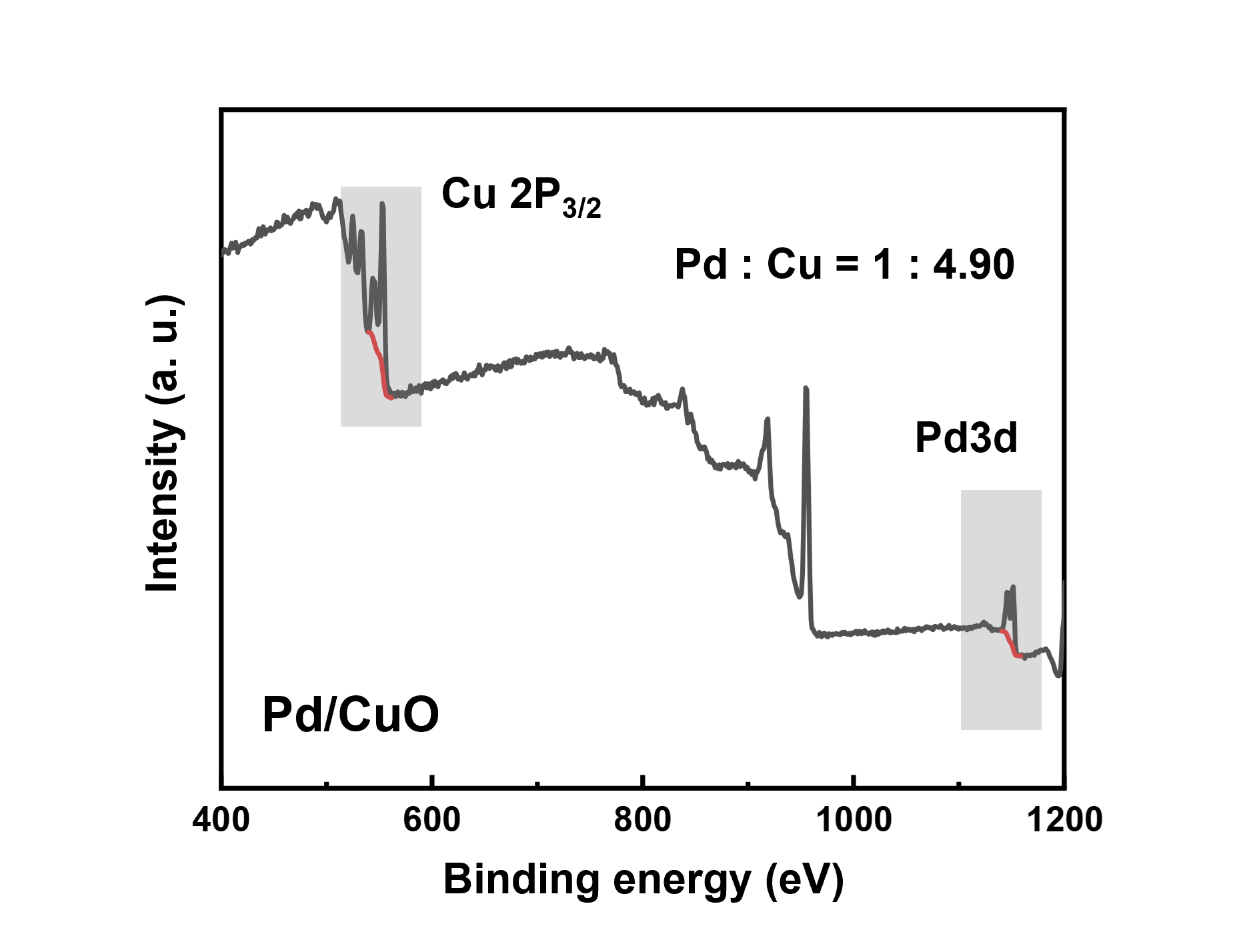


**Figure S15.** Survey XPS spectrum of Pd/CuO.


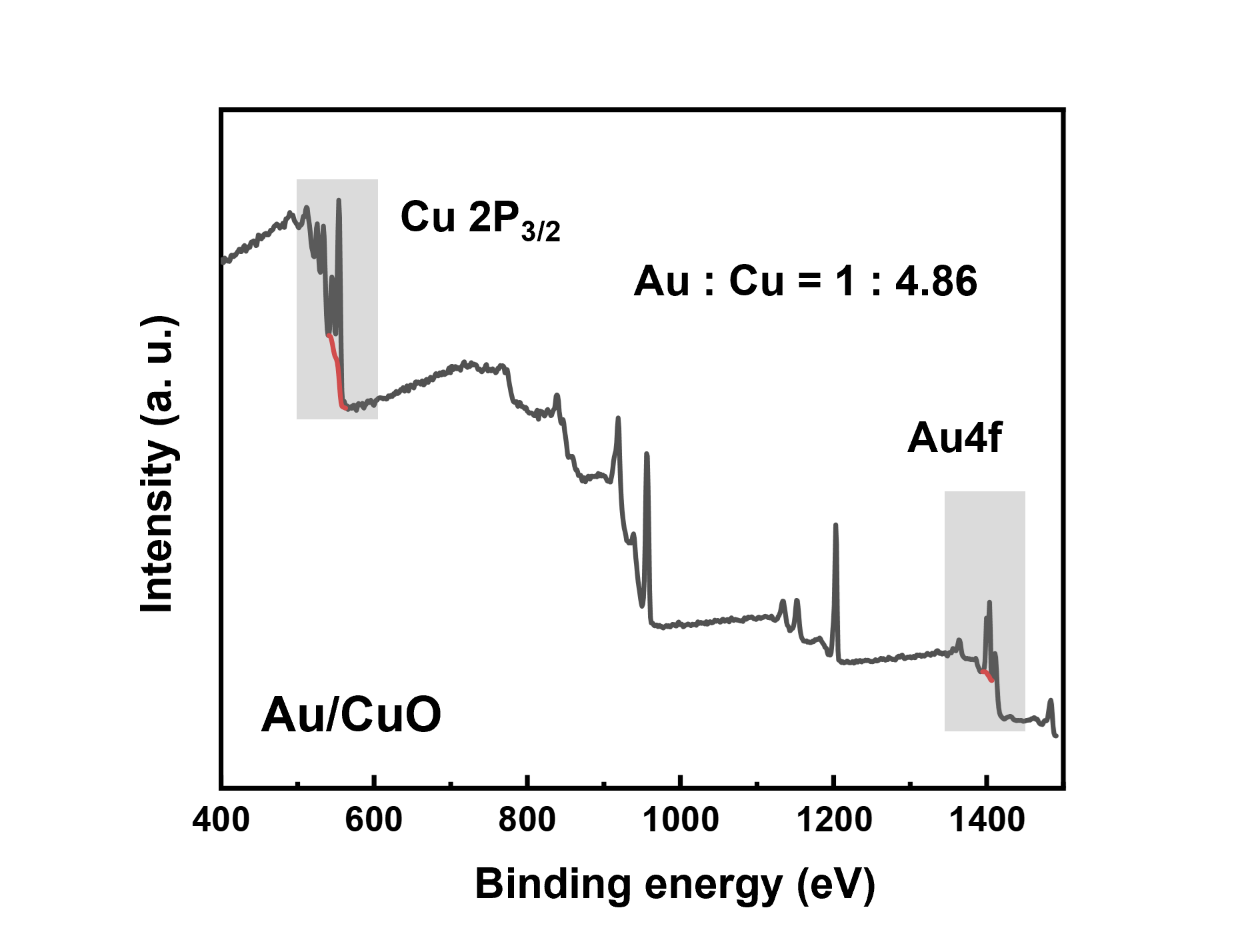


**Figure S16.** Survey XPS spectrum of Au/CuO.


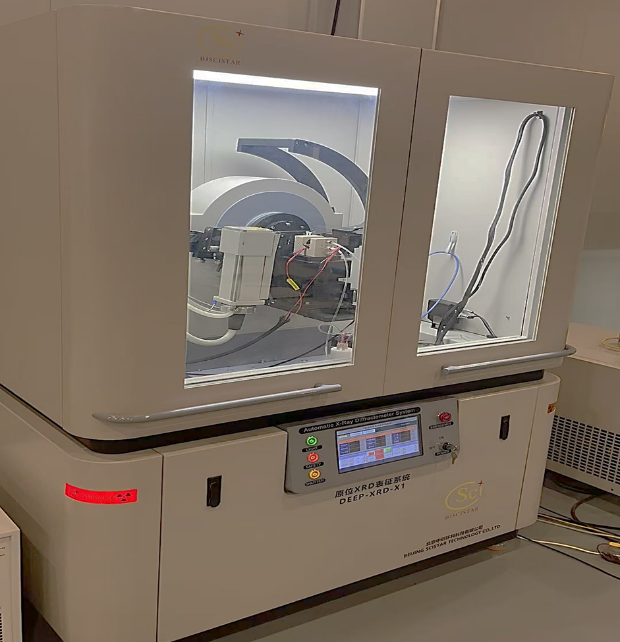


**Figure S17.** Photo in situ XRD instruments for ECR.


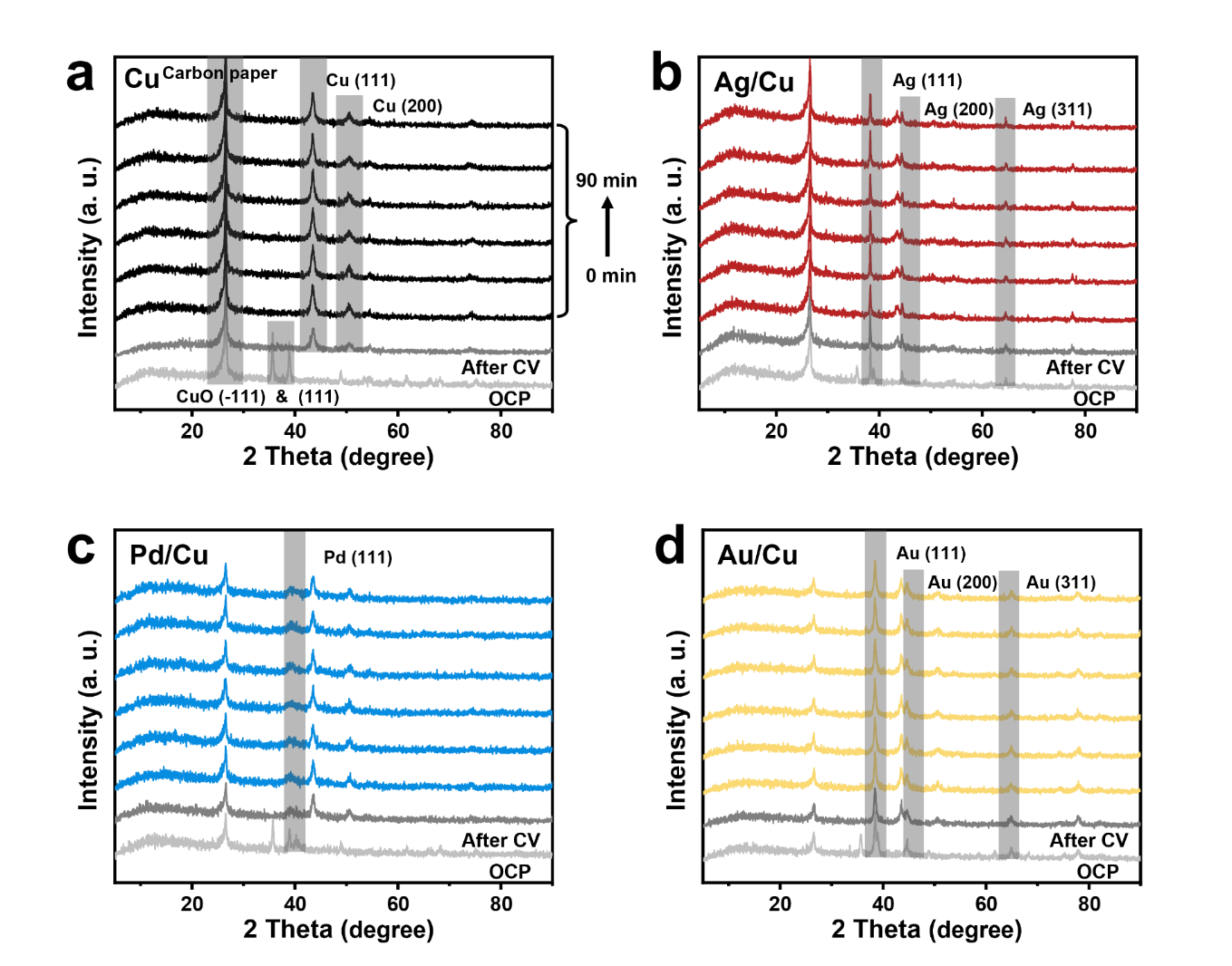


**Figure S18.** **(a-d)** In situ XRD patterns of Cu, Ag/Cu, Pd/Cu, and Au/Cu catalysts.


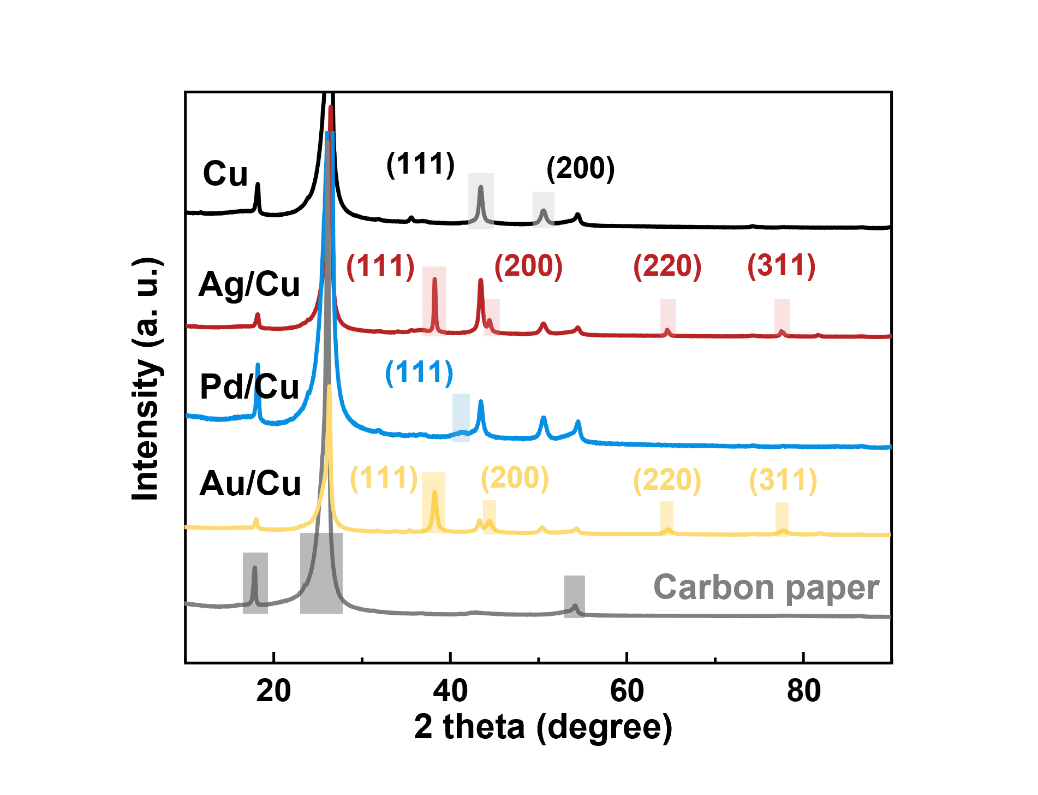


**Figure S19.** Ex situ XRD patterns of Cu, Ag/Cu, Pd/Cu, and Au/Cu catalyst.


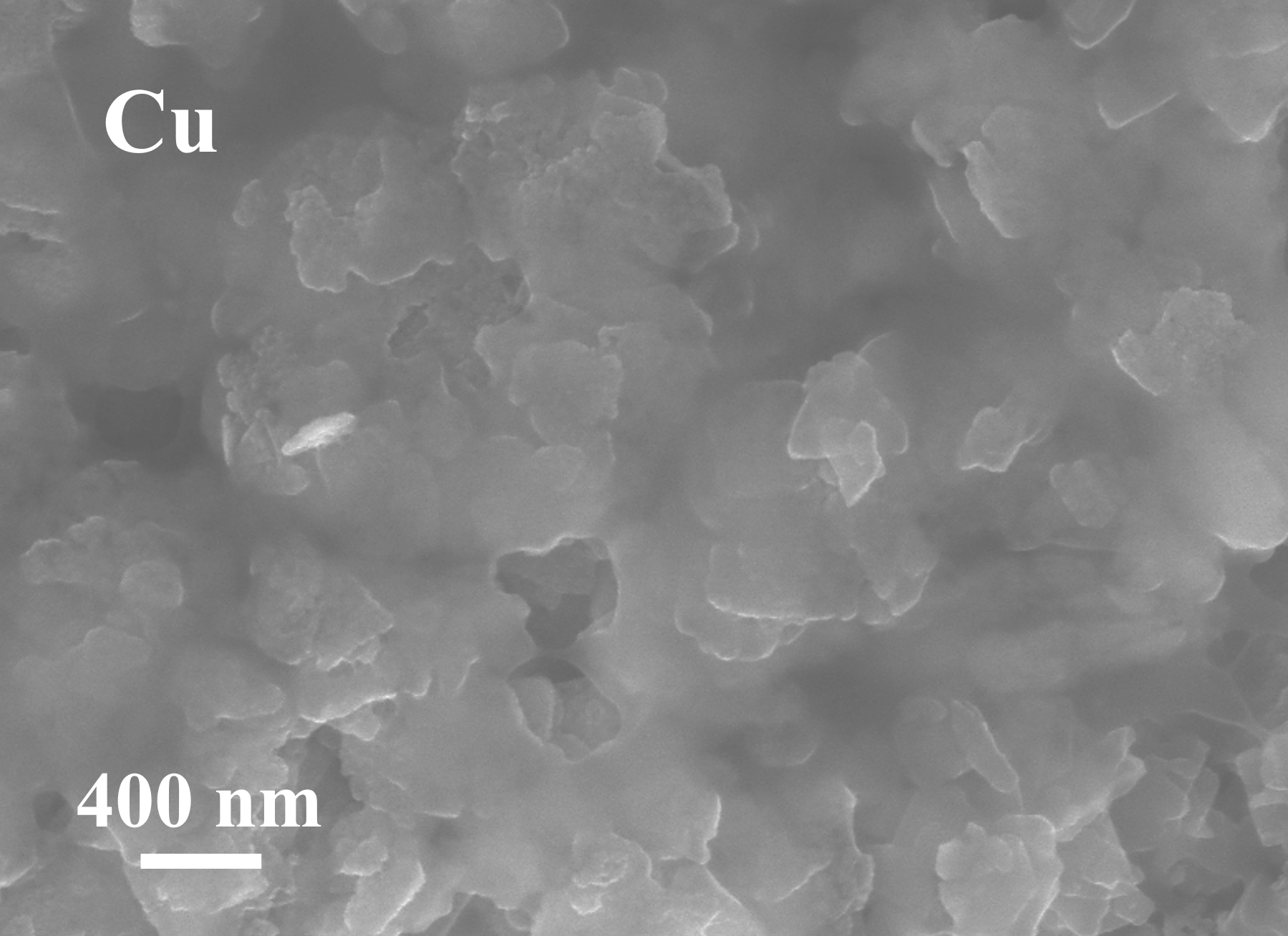


**Figure S20.** SEM image of Cu after CV activation.


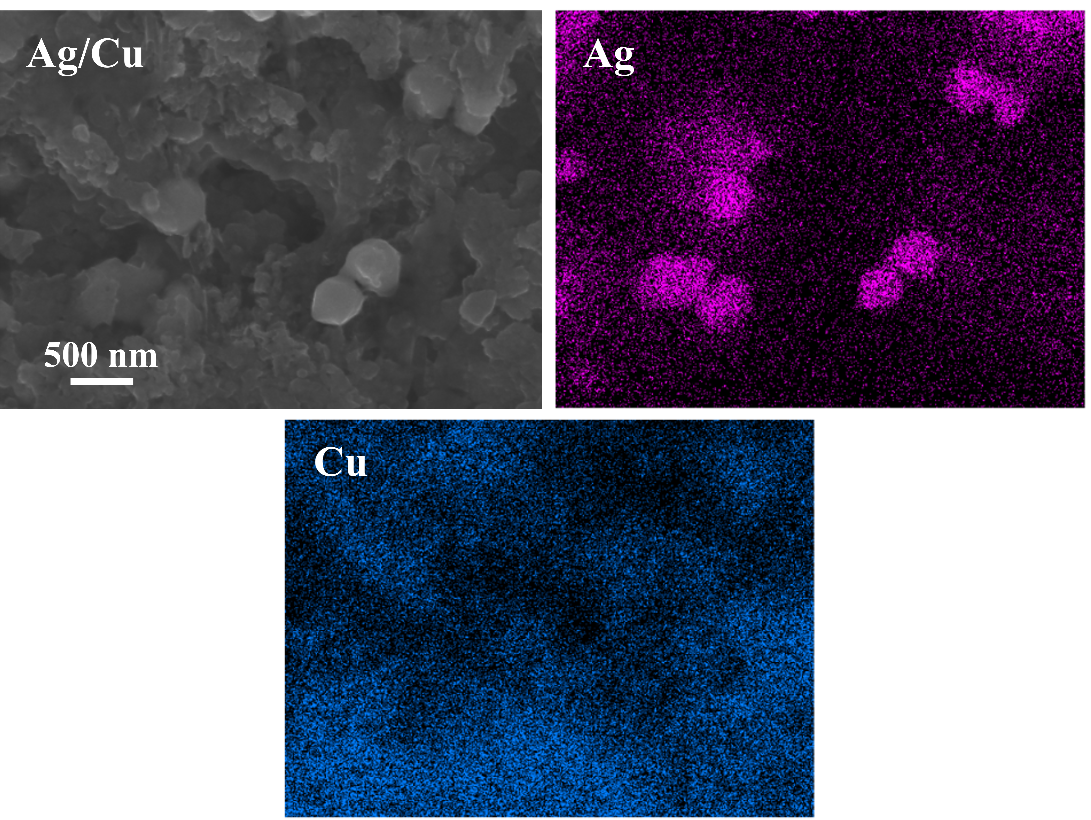


**Figure S21.** SEM image and elemental mappings of Ag/Cu after CV activation.


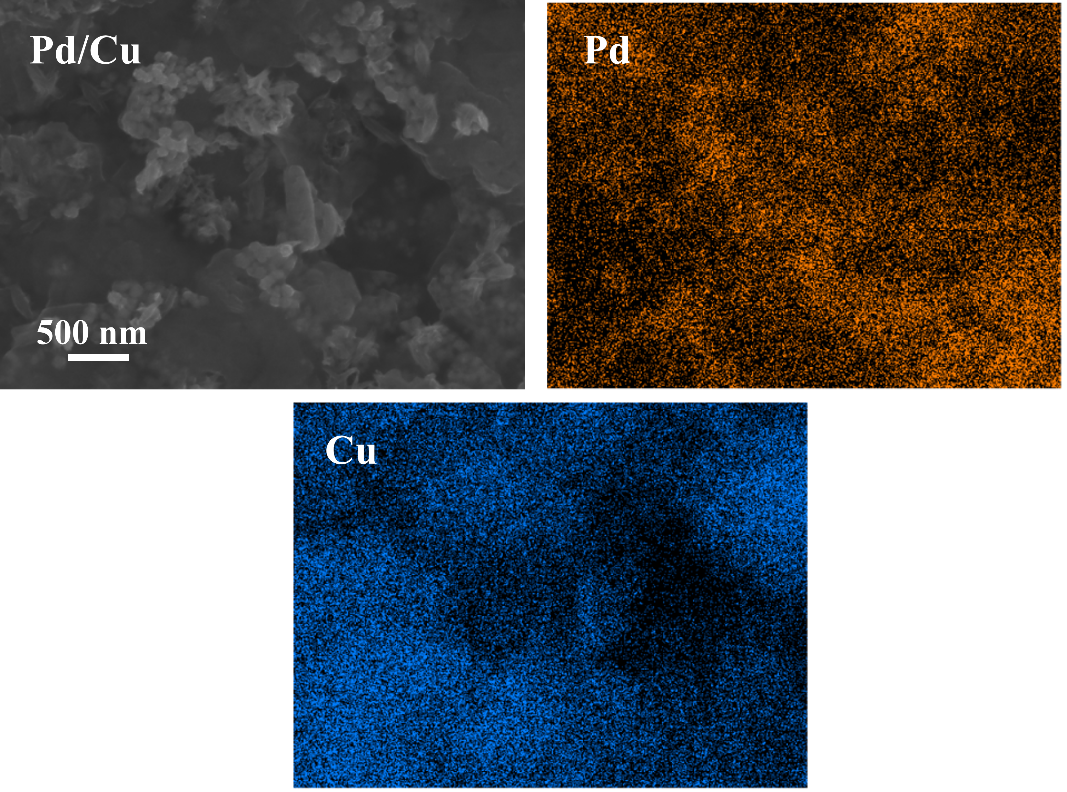


**Figure S22.** SEM image and elemental mappings of Pd/Cu after CV activation.


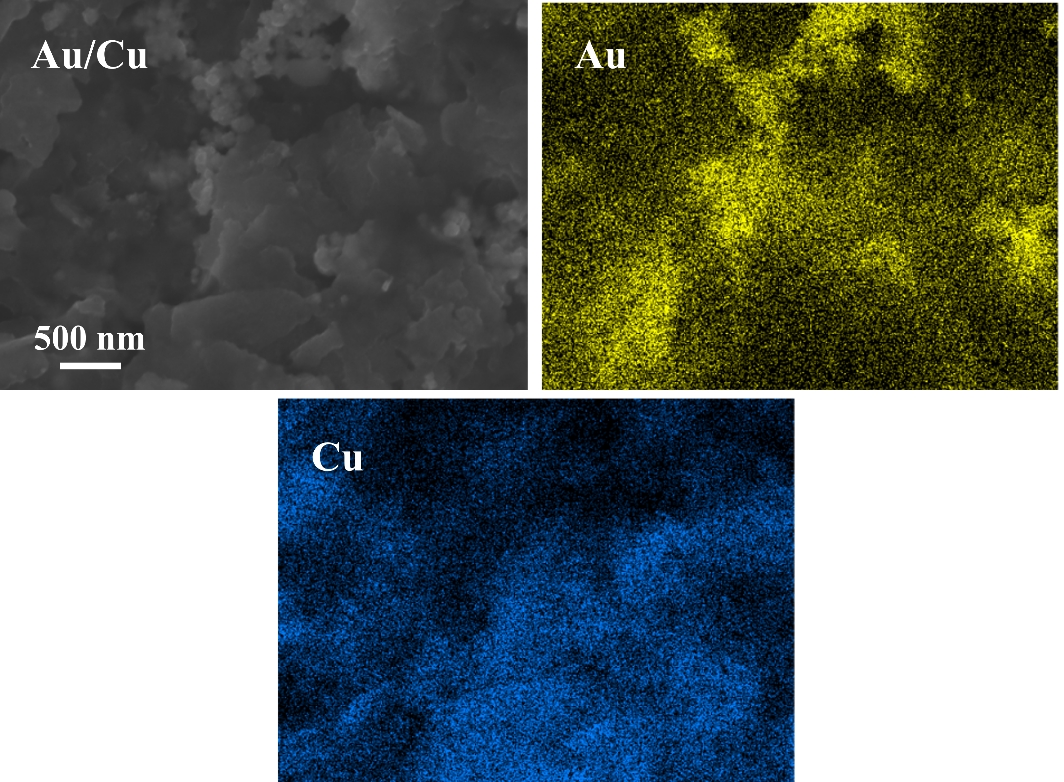


**Figure S23.** SEM image and elemental mappings of Au/Cu after CV activation.


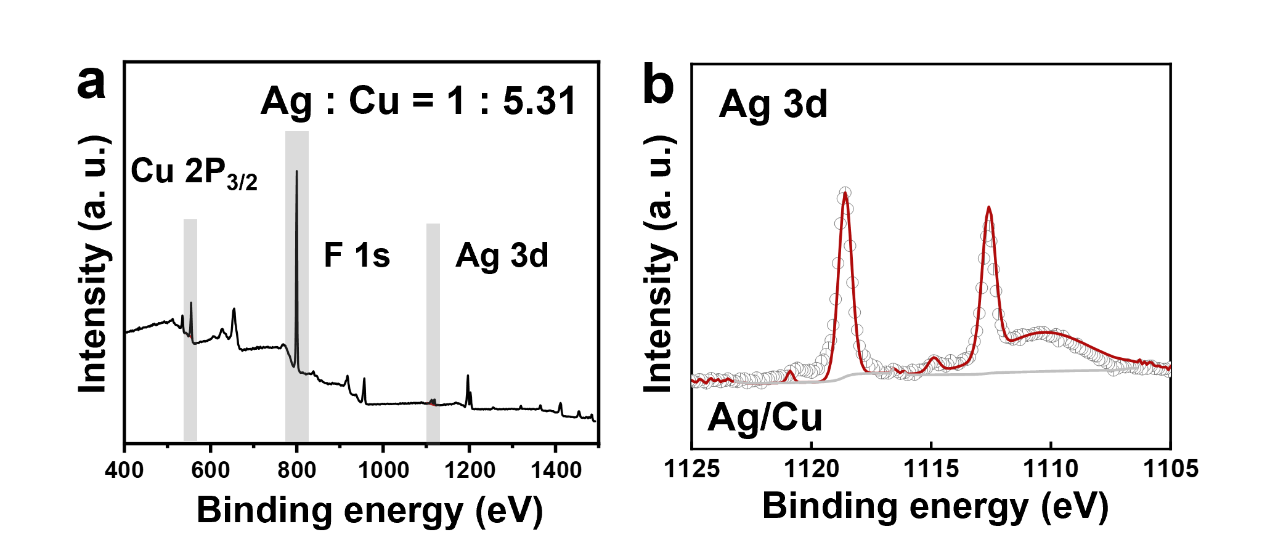


**Figure S24.** **(a)** Survey XPS and **(b)** Ad 3d spectra of Ag/Cu after CV activation.


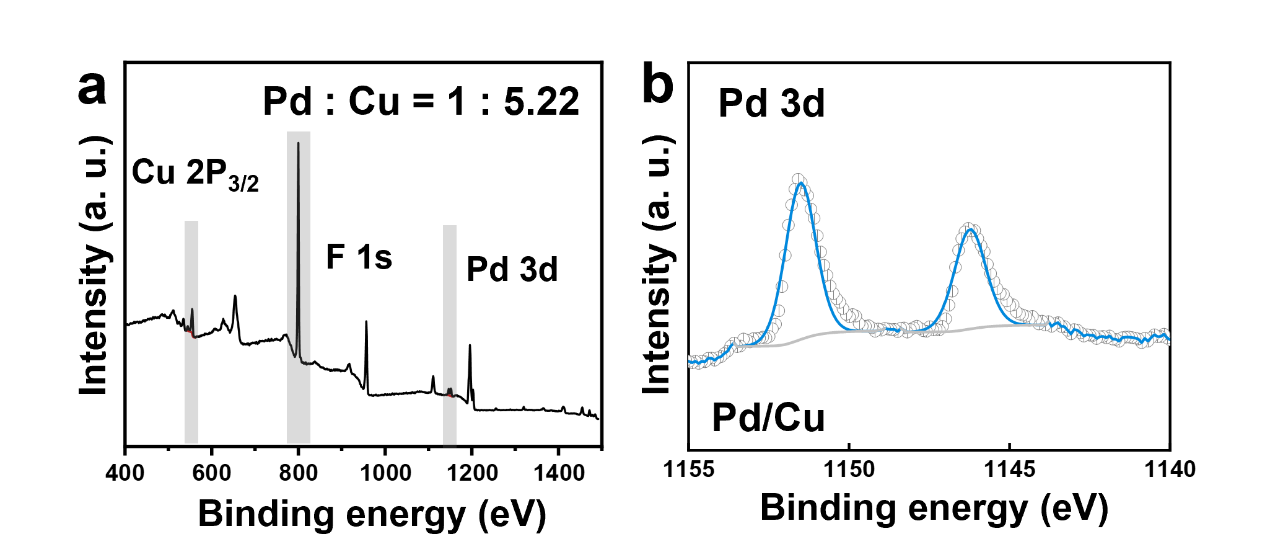


**Figure S25.** **(a)** Survey XPS and **(b)** Pd 3d spectra of Pd/Cu after CV activation.


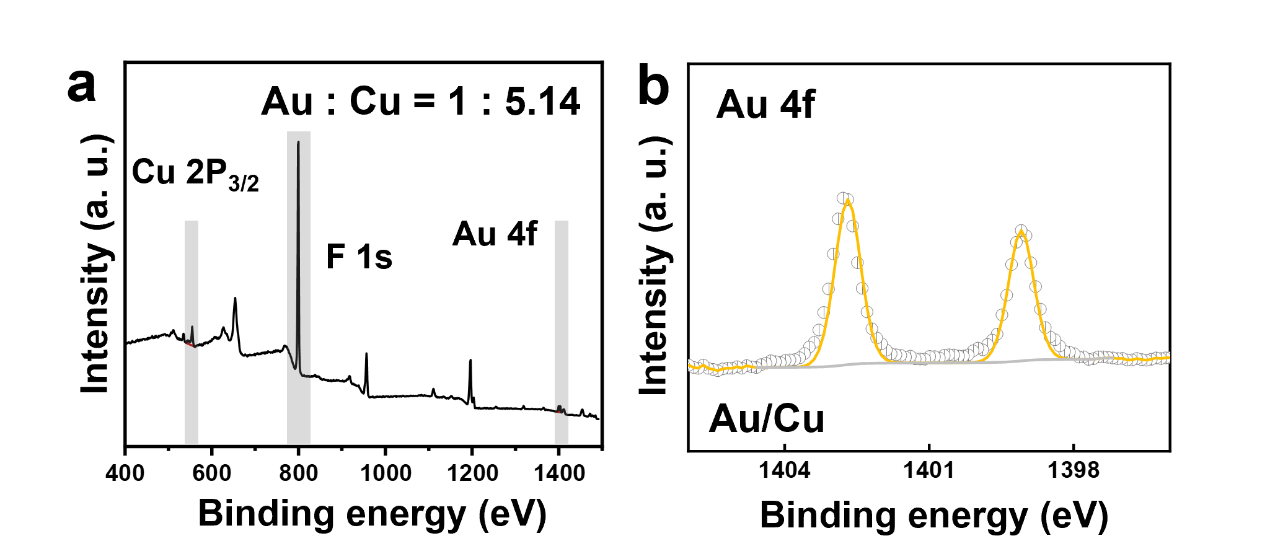


**Figure S26.** **(a)** Survey XPS and **(b)** Au 4f spectra of Au/Cu after CV activation.


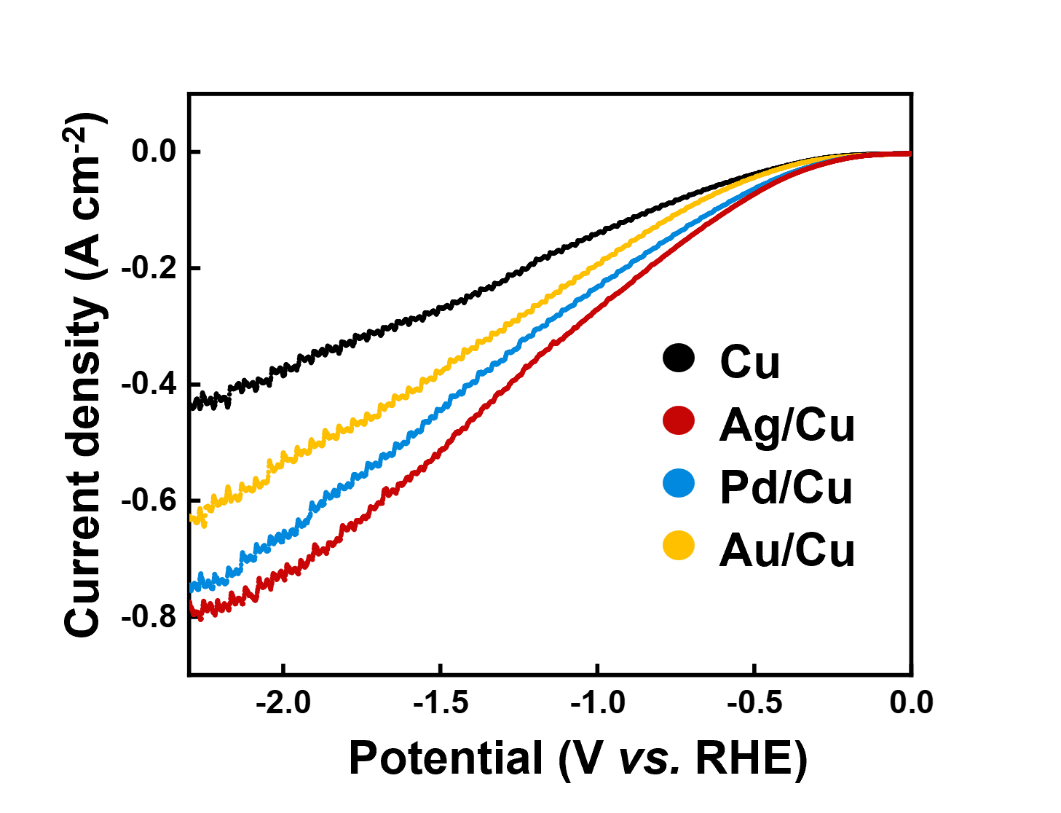


**Figure S27.** LSV curves of Cu, Ag/Cu, Pd/Cu and Au/Cu catalysts


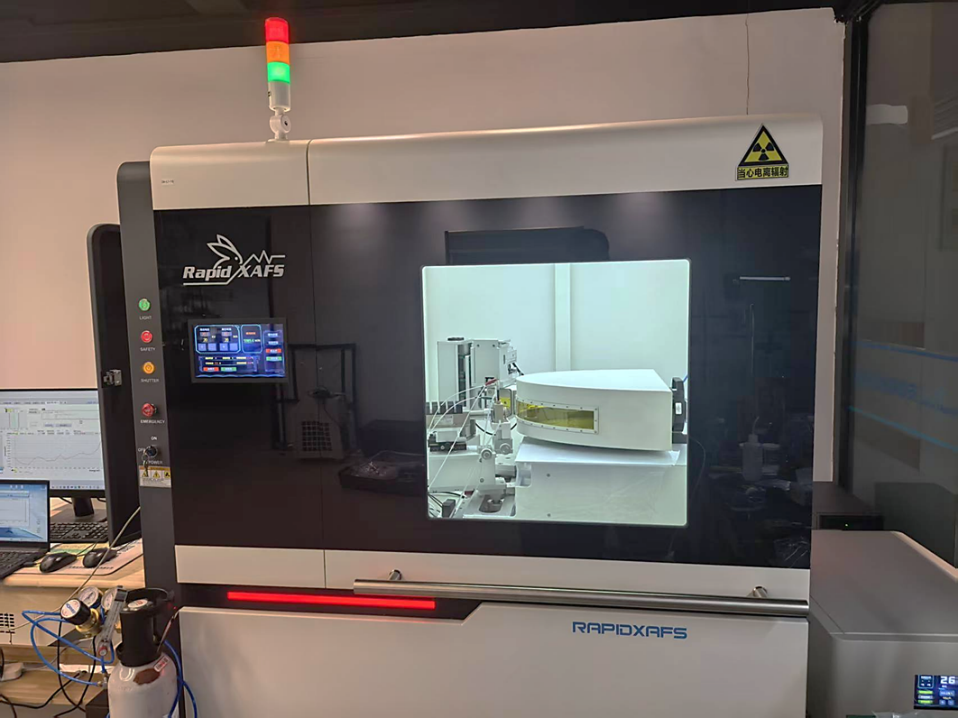


**Figure S28.** Photo of in situ XAFS spectrometer.


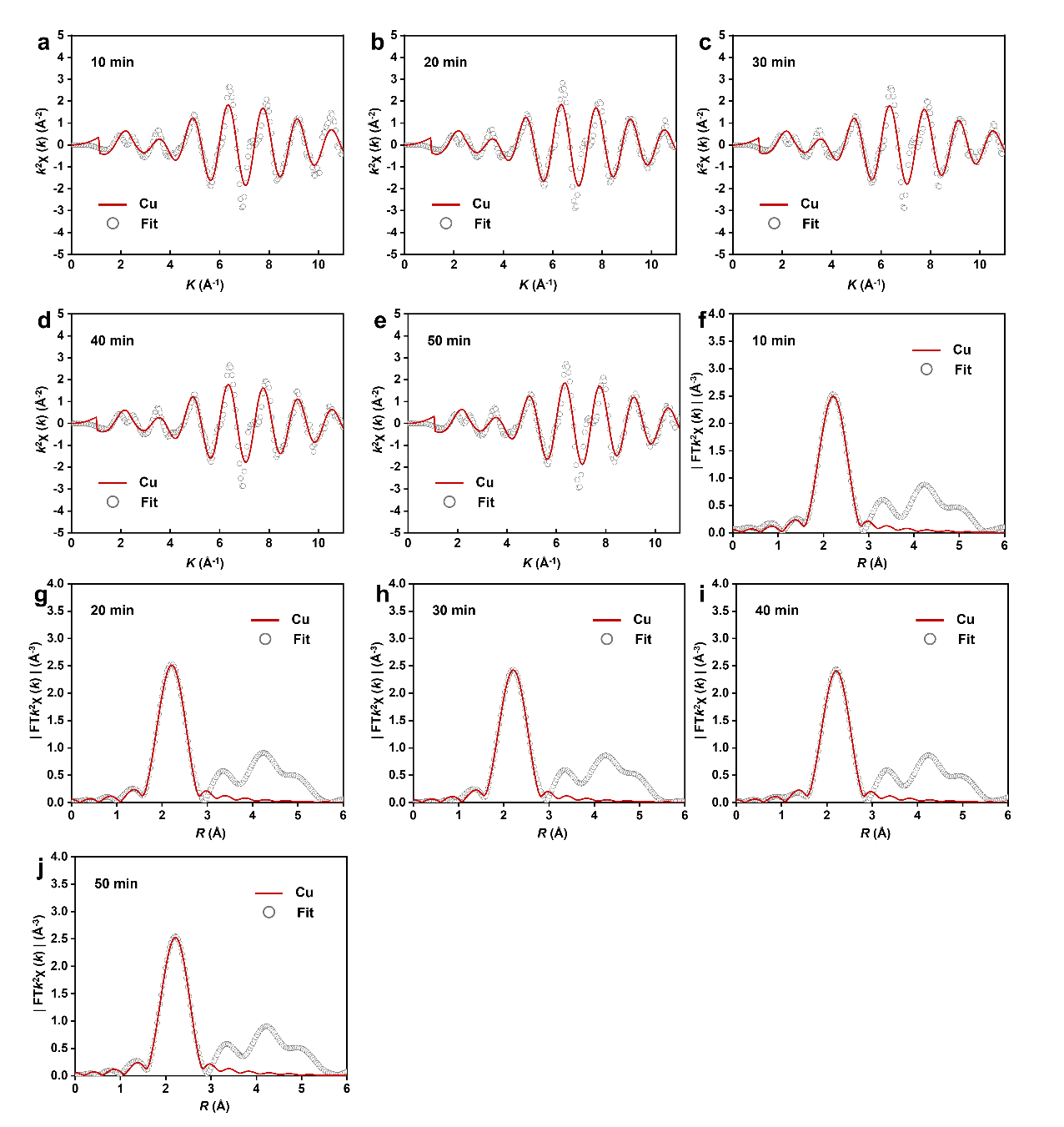


**Figure S29.** **(a-e)** K-space and **(f-j)** R-space of Cu K-edge EXAFS fitting curves with time evolution (10-50 min) during ECR process for Cu catalyst.


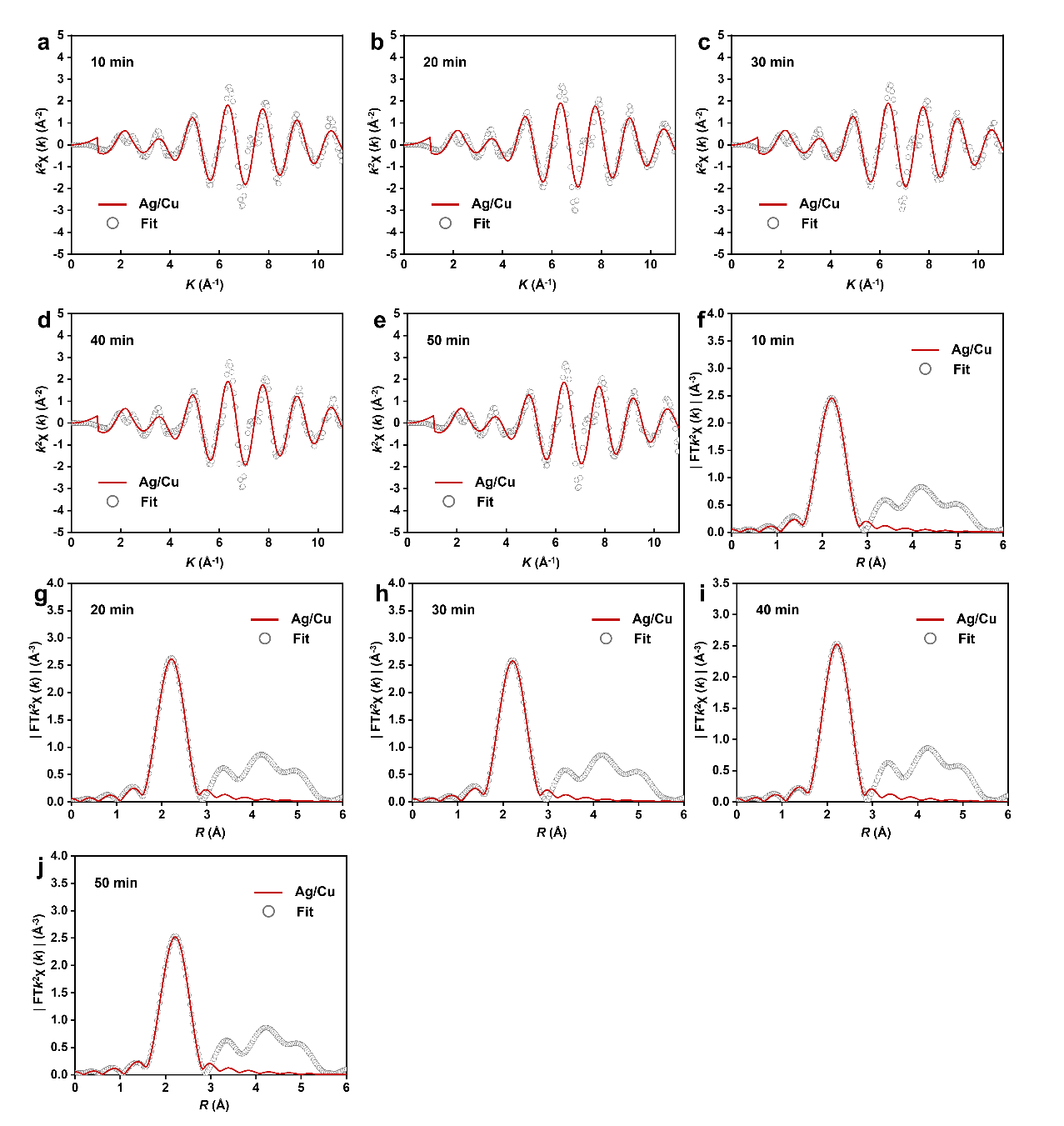


**Figure S30.** **(a-e)** K-space and **(f-j)** R-space of Cu K-edge EXAFS fitting curves with time evolution (10-50 min) during ECR process for Ag/Cu catalyst.


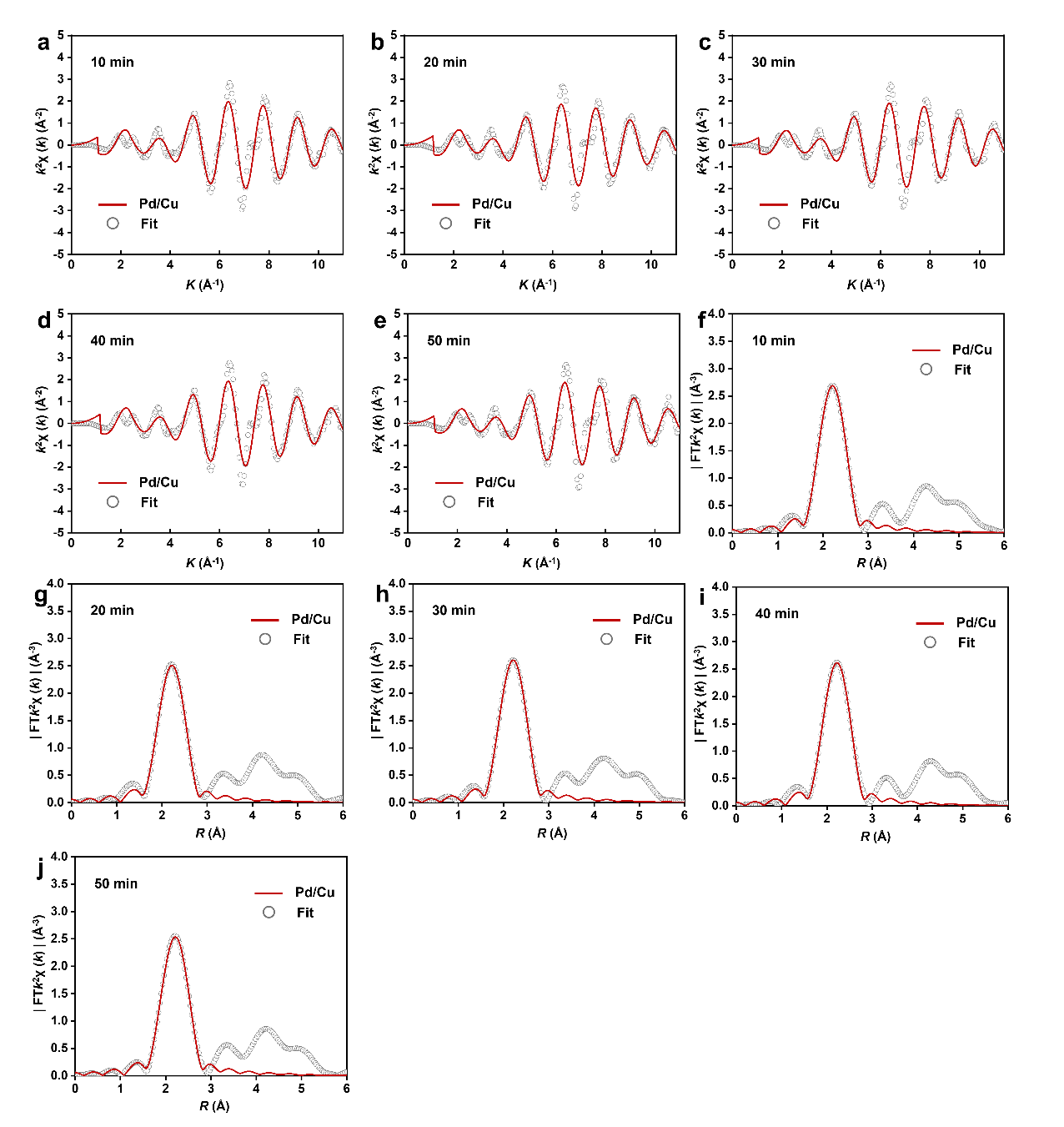


**Figure S31.** **(a-e)** K-space and **(f-j)** R-space of Cu K-edge EXAFS fitting curves with time evolution (10-50 min) during ECR process for Pd/Cu catalyst.


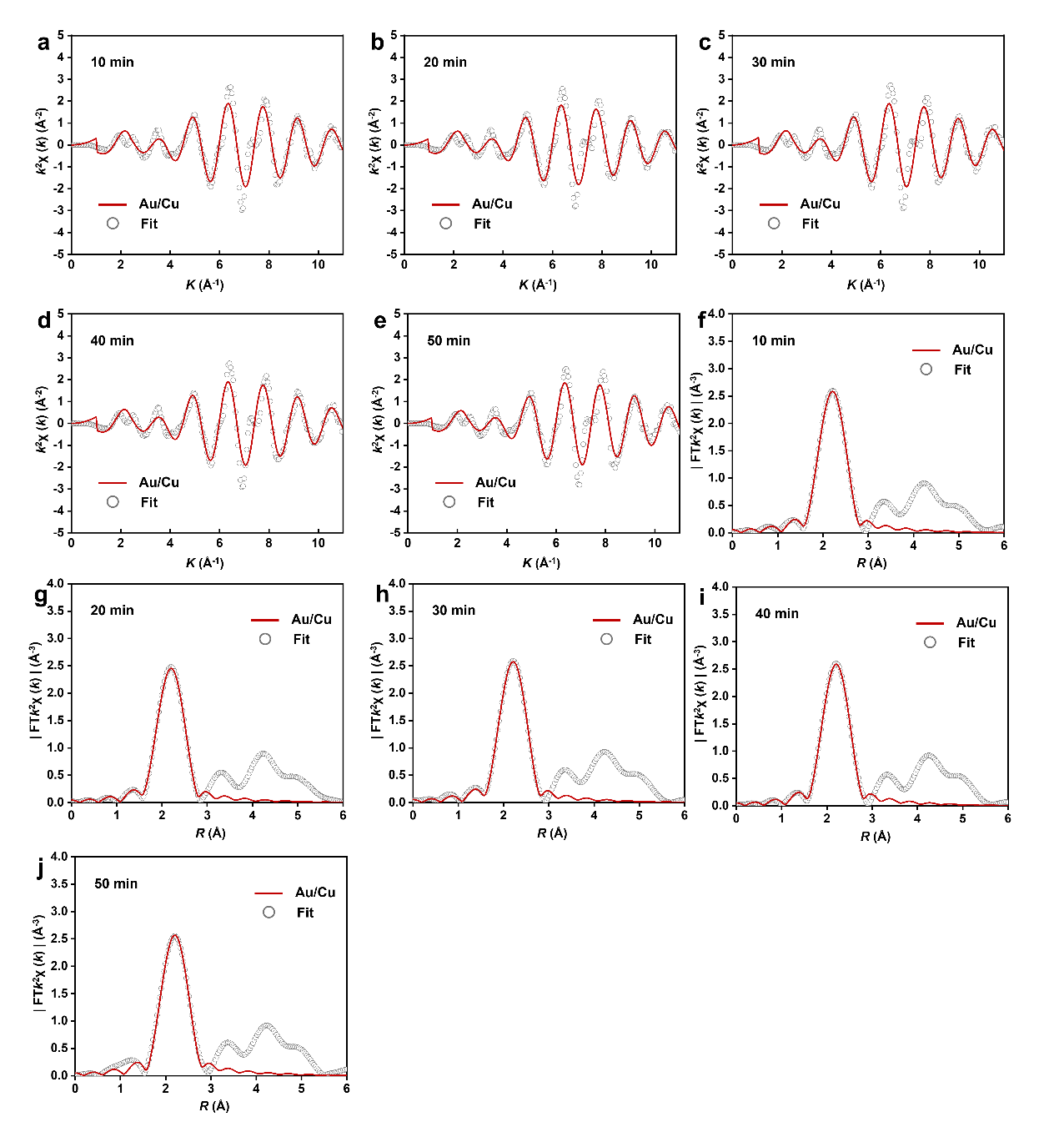


**Figure S32.** **(a-e)** K-space and **(f-j)** R-space of Cu K-edge EXAFS fitting curves with time evolution (10-50 min) during ECR process for Au/Cu catalyst.


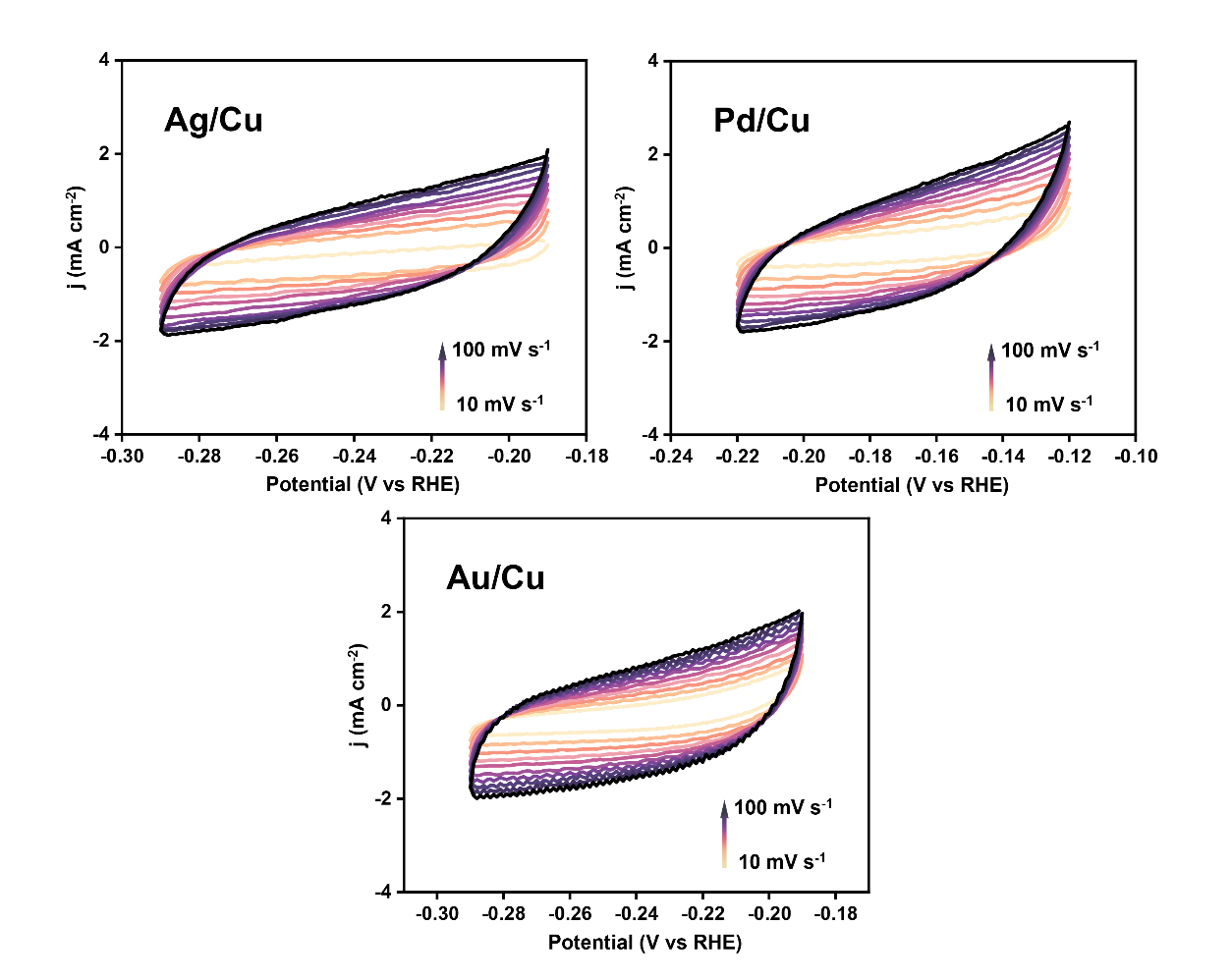


**Figure S33.** CV curves of Cu, Ag/Cu, Pd/Cu, and Au/Cu catalysts at different scan rates.


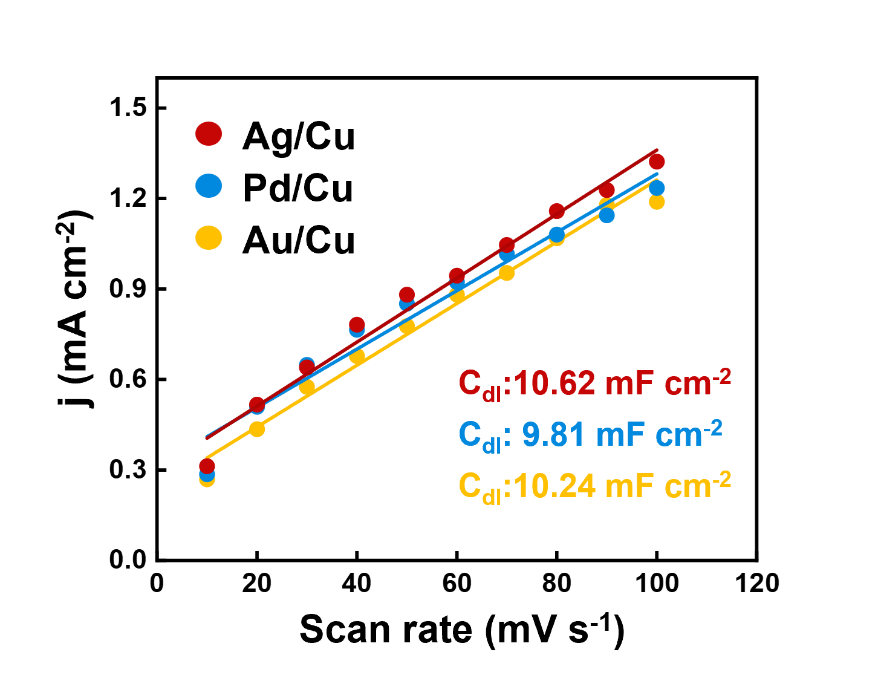


**Figure S34.** The charging current density differences plotted against the scan rates and C_dl_ values of Ag/Cu, Pd/Cu, and Au/Cu catalysts.


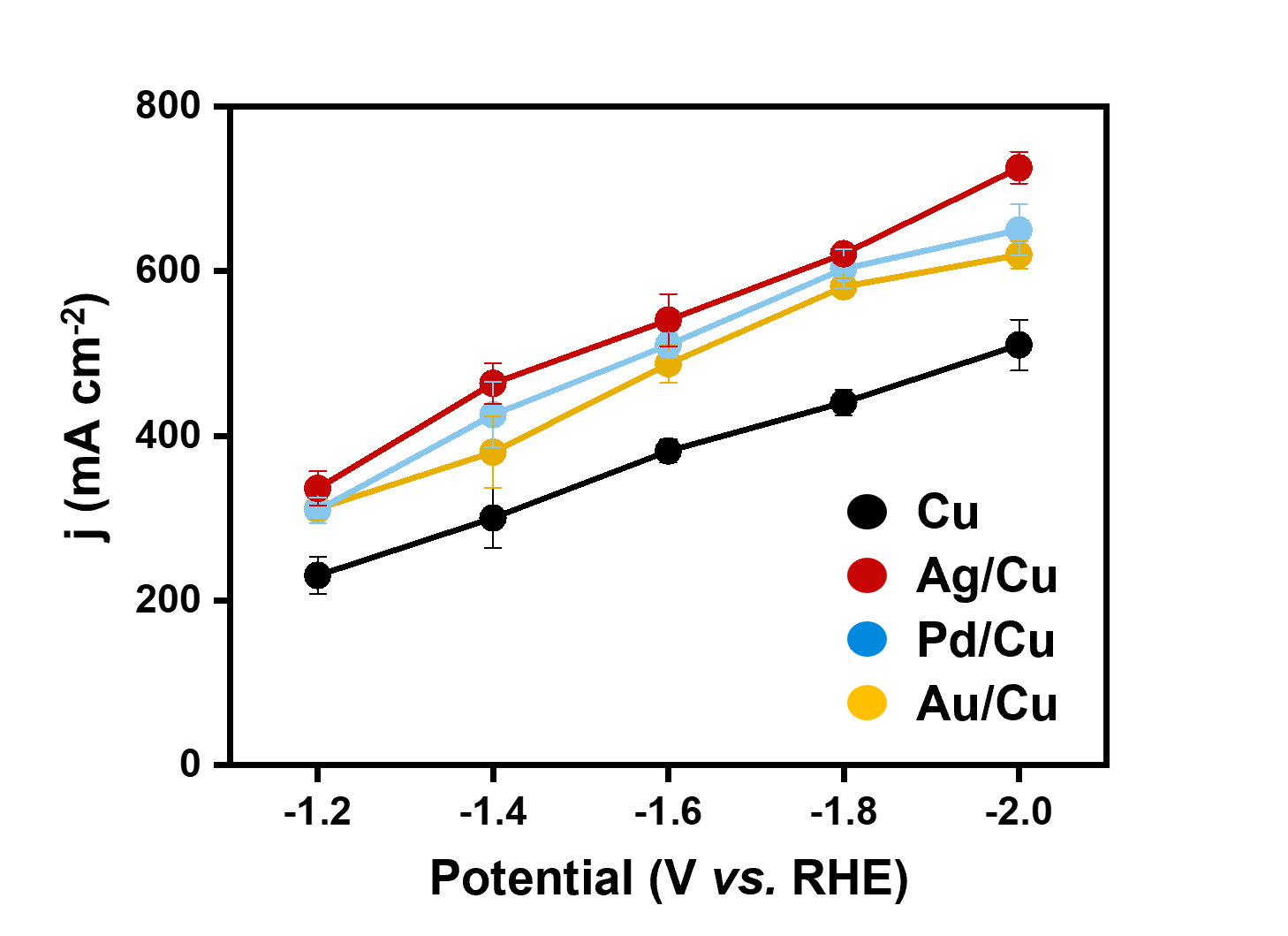


**Figure S35.** Current densities of Cu, Ag/Cu, Pd/Cu, and Au/Cu catalysts.


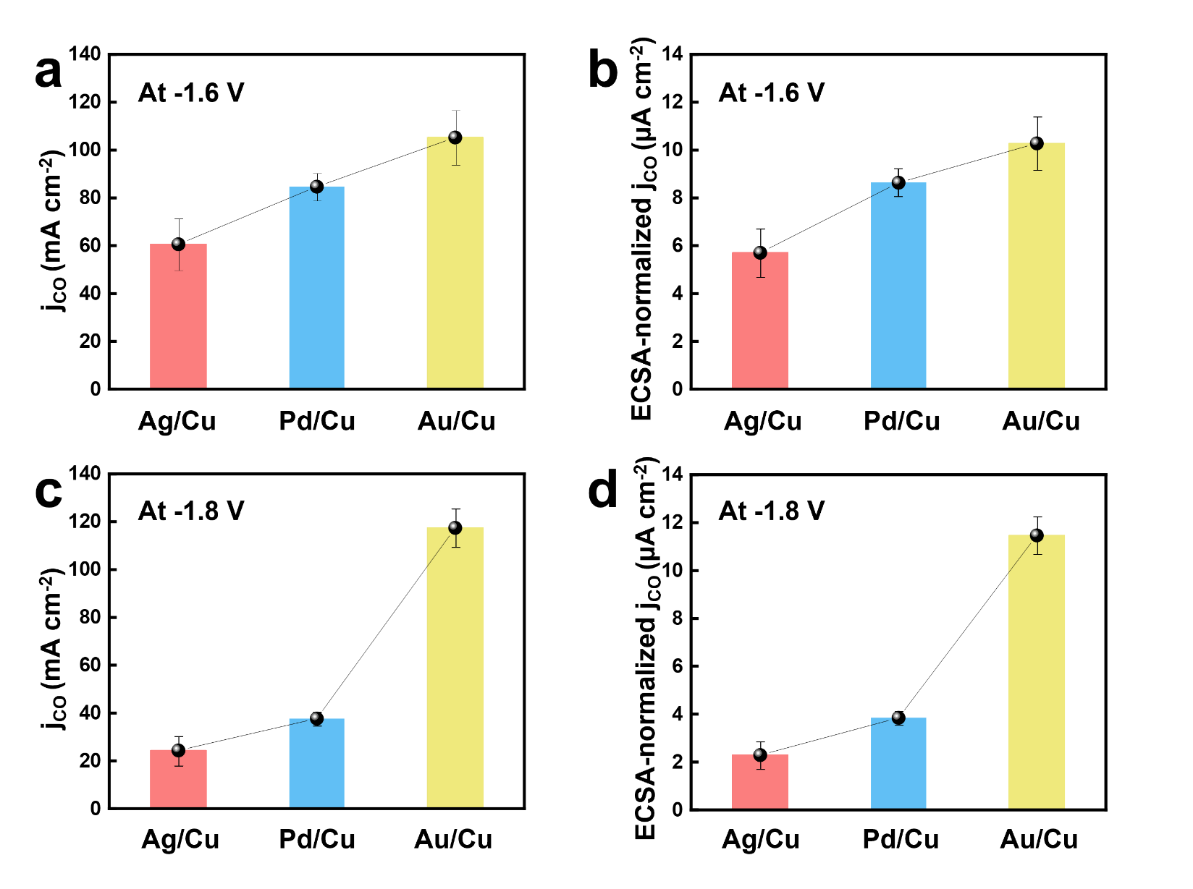


**Figure S36.** Partial current density of CO product and ECSA-normalized average current density of values of Ag/Cu, Pd/Cu, and Au/Cu catalysts at (a, b) -1.6 V and (c, d) -1.8 V.


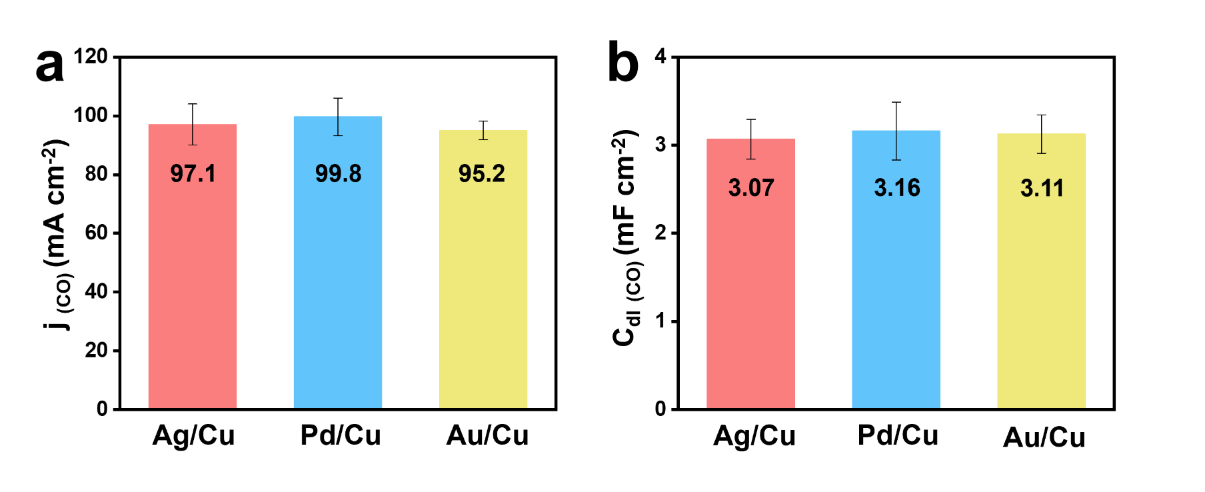


**Figure S37.** **(a)** Partial current density of CO (j_CO_) and **(b)** double layer capacitance contributed to produce CO (C_dl (CO)_) of Ag/Cu, Pd/Cu, and Au/Cu catalysts at -1.2 V.


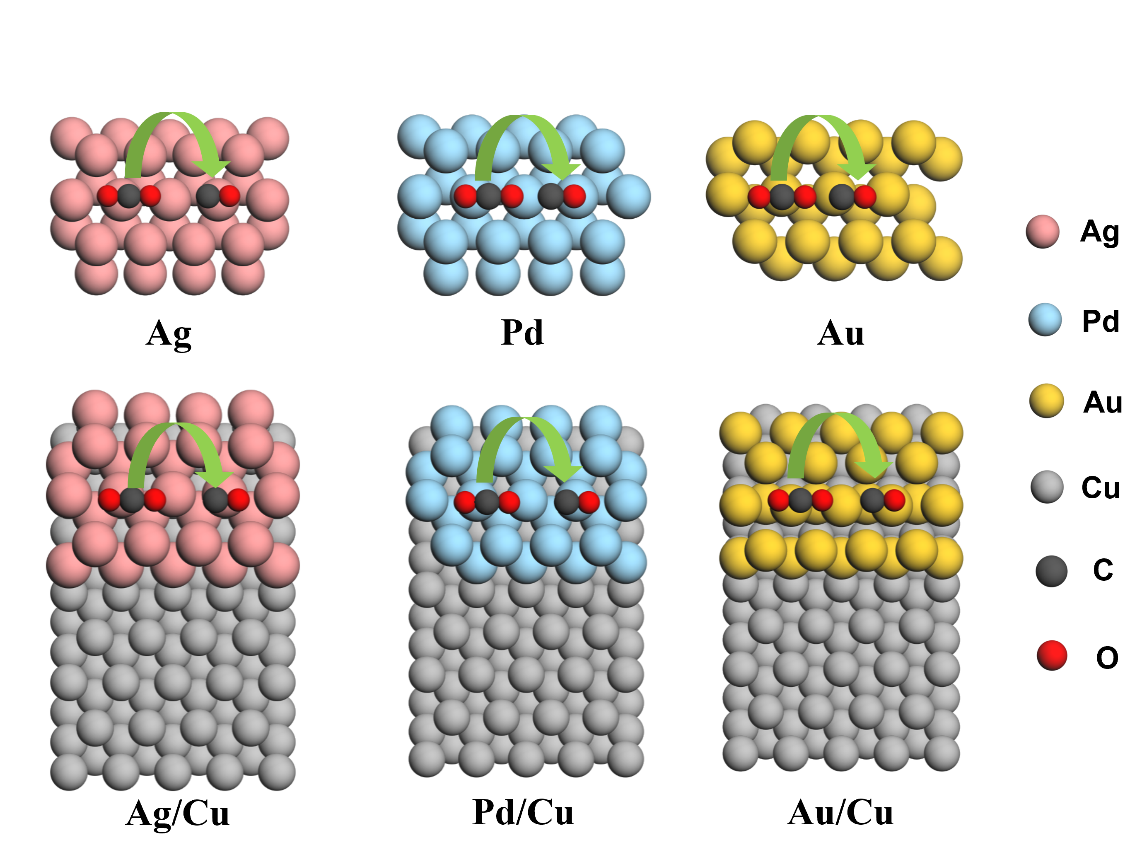


**Figure S38.** Schematic configurations of *CO_2_ to *CO over Ag, Pd, Au, Ag/Cu, Pd/Cu, and Au/Cu calculation models (*CO_2_ and *CO represent adsorbed species).


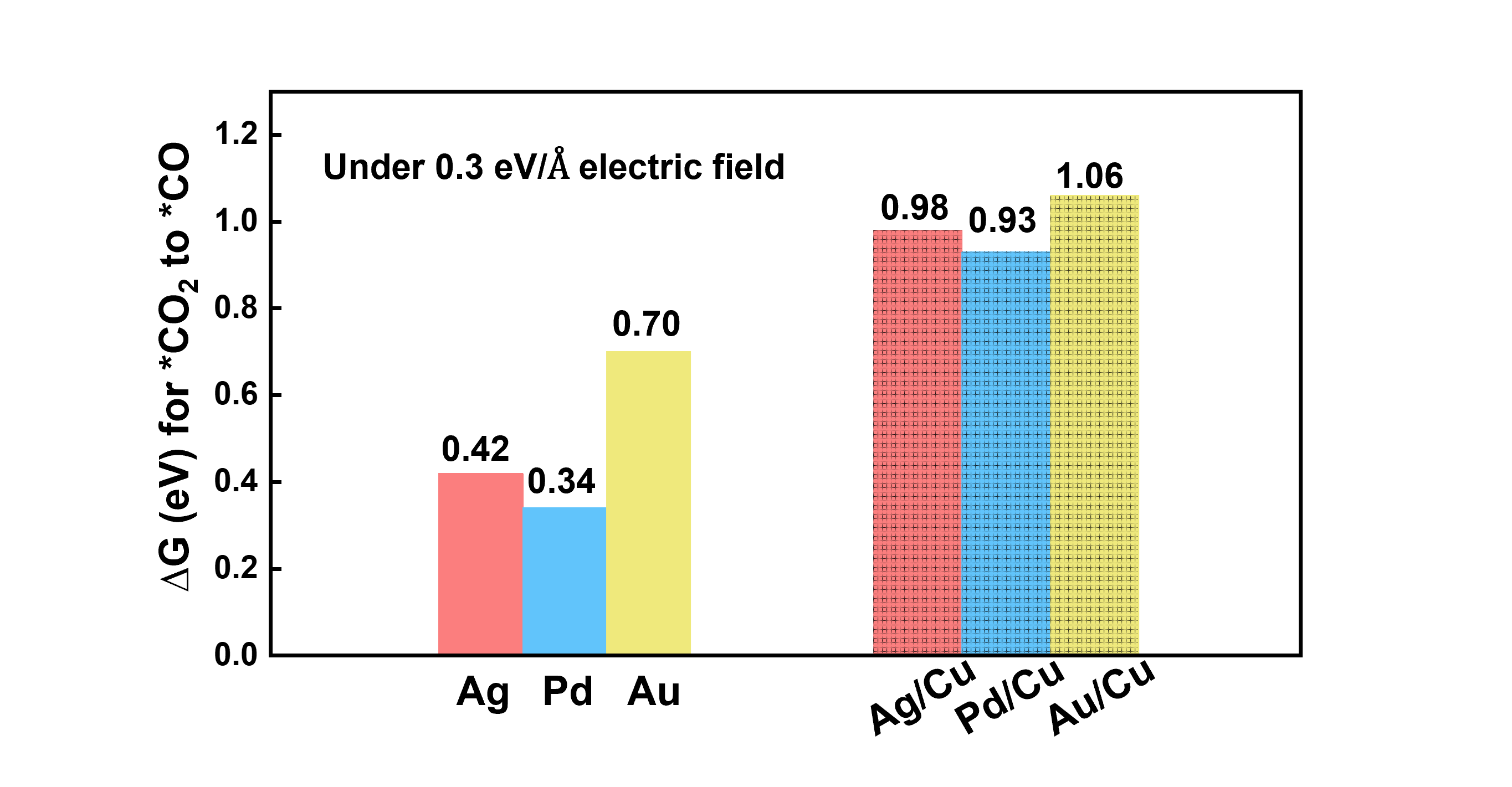


**Figure S39.** The Gibbs free energy diagram for *CO_2_ to *CO over Ag, Pd, Au, Ag/Cu, Pd/Cu, and Au/Cu calculation models (*CO_2_ and *CO represent adsorbed species).


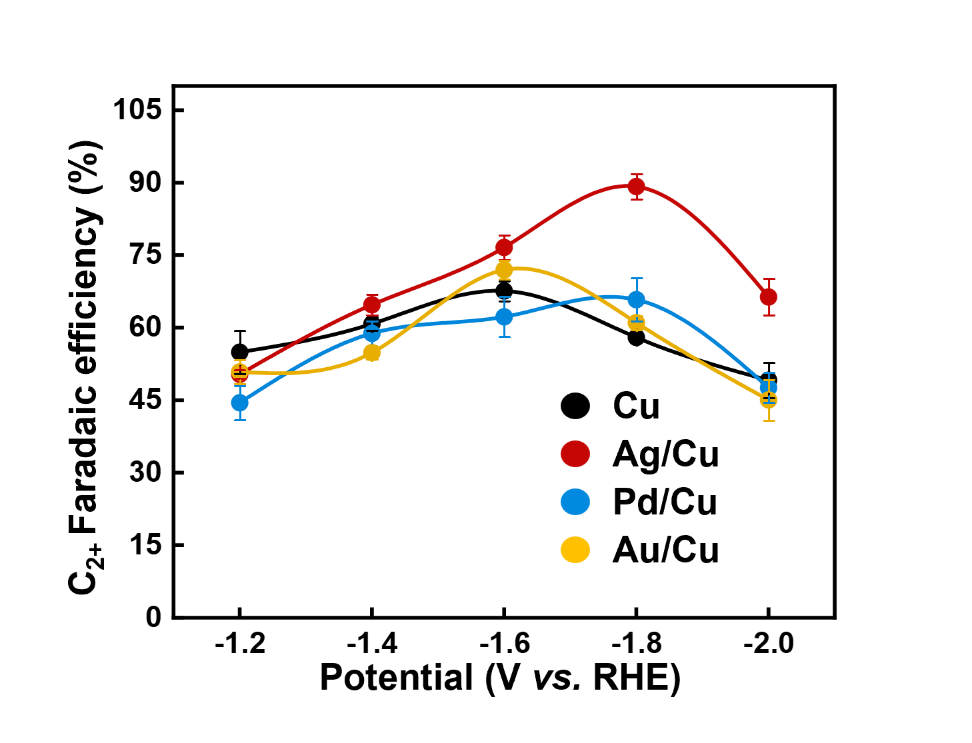


**Figure S40.** C_2+_ Faradaic efficiency of Cu, Ag/Cu, Pd/Cu, and Au/Cu catalysts.


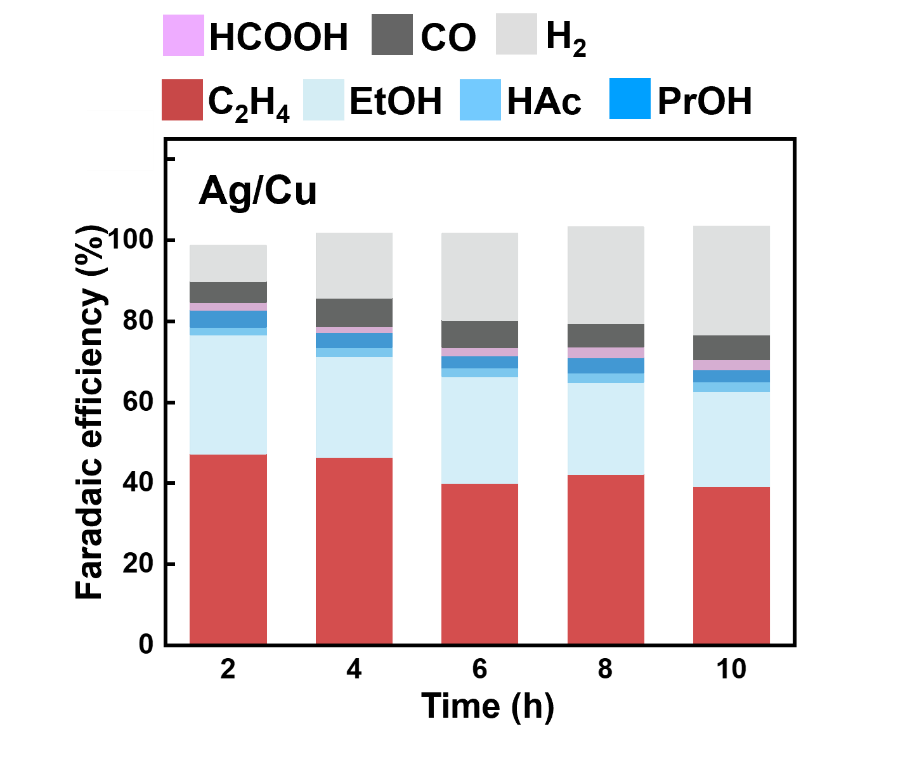


**Figure S41.** Product distributions of Ag/Cu catalyst at -1.8 V during the long-term stability test.


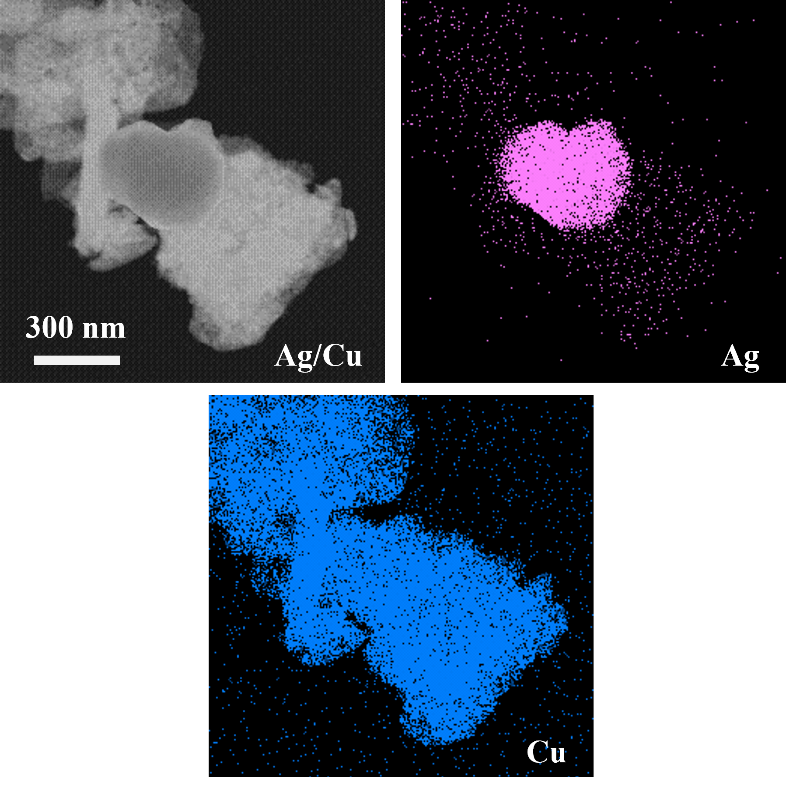


**Figure S42.** TEM image and elemental mappings of Ag/Cu after CV activation before long-term stability tests. The molar ratio of Ag : Cu is 1 : 5.53 as detected by EDX-TEM.


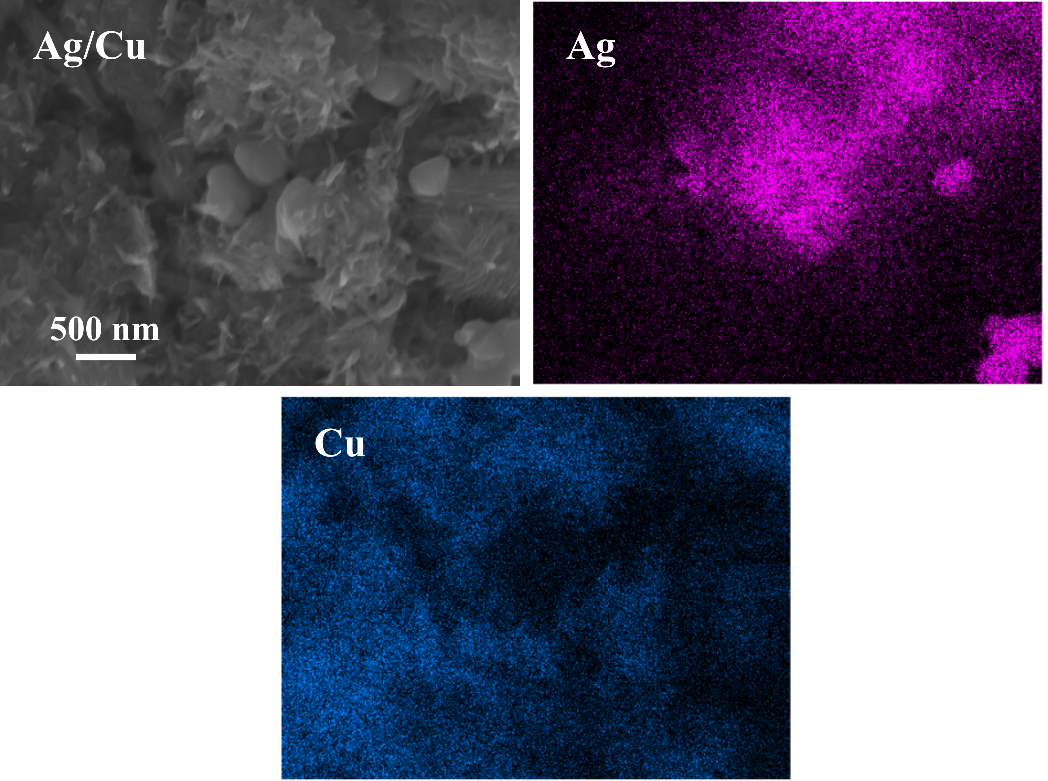


**Figure S43.** SEM image and elemental mappings of Ag/Cu after long-term stability test.


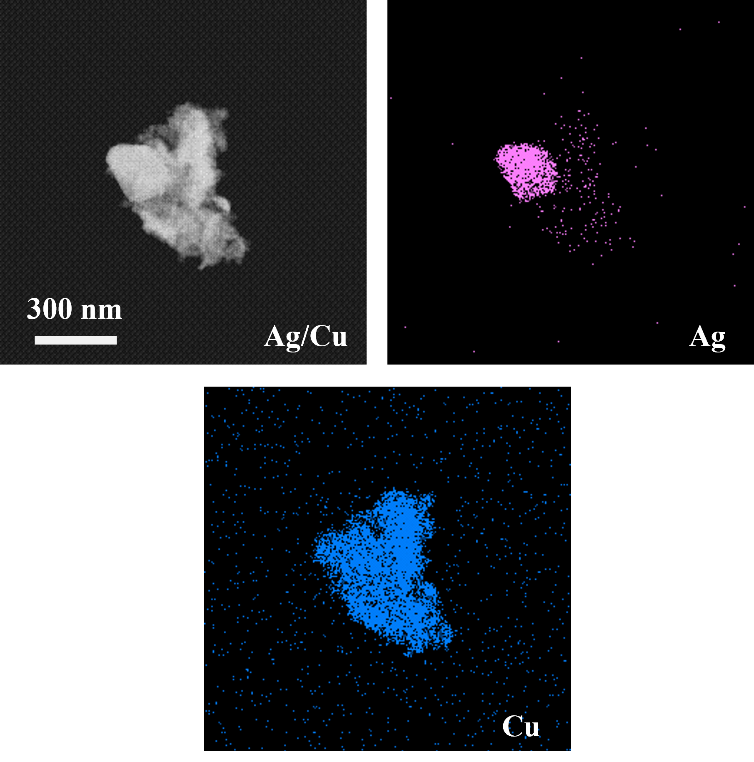


**Figure S44.** TEM image and elemental mappings of Ag/Cu after long-term stability test. The molar ratio of Ag : Cu is 1 : 6.17 as detected by EDX-TEM.


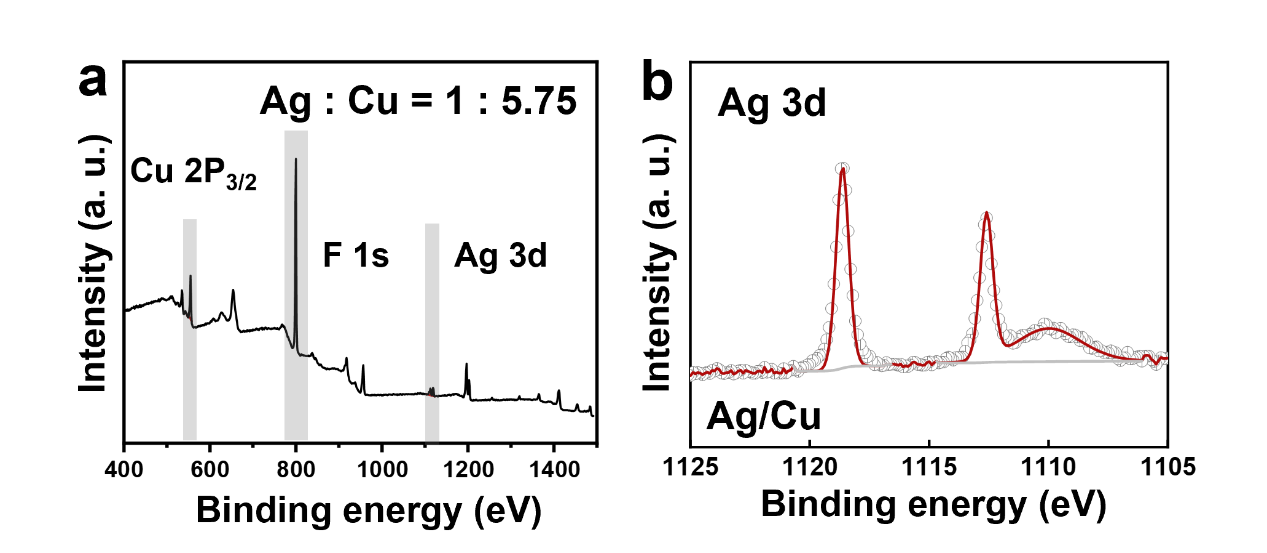


**Figure S45.** **(a)** survey XPS and **(b)** Ad 3d spectra of Ag/Cu after long-term stability test. The molar ratio of Ag : Cu is 1 : 5.75 as detected by XPS spectra.


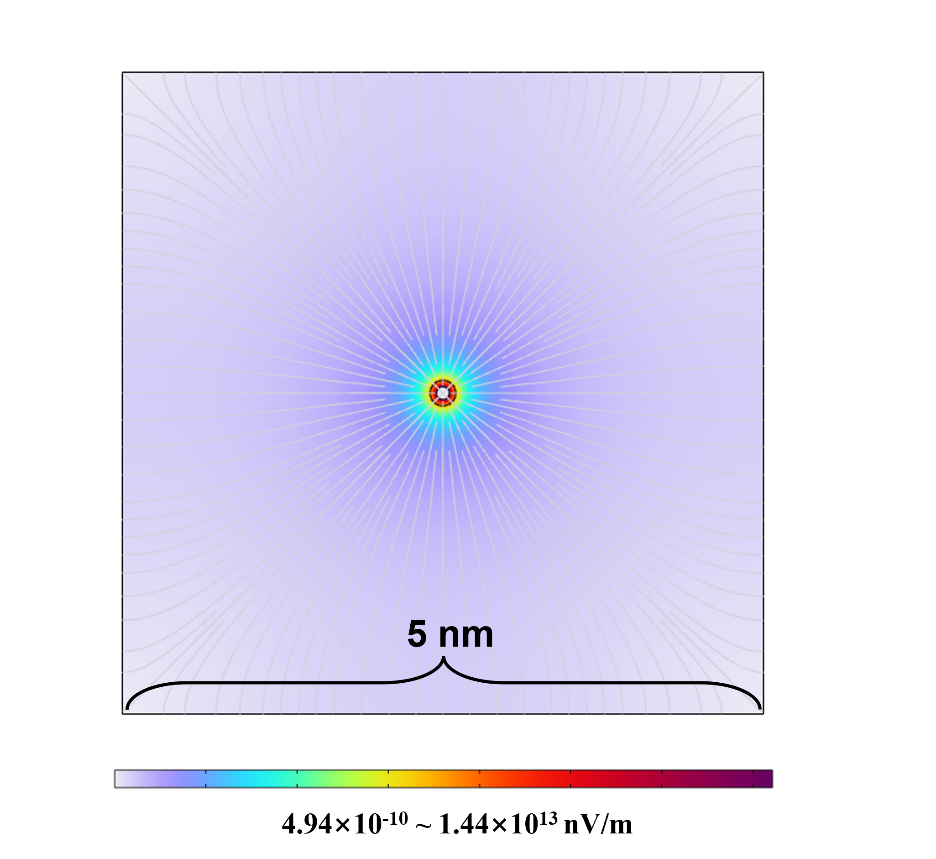


**Figure S46.** Electric field distribution for “5 nm” model.


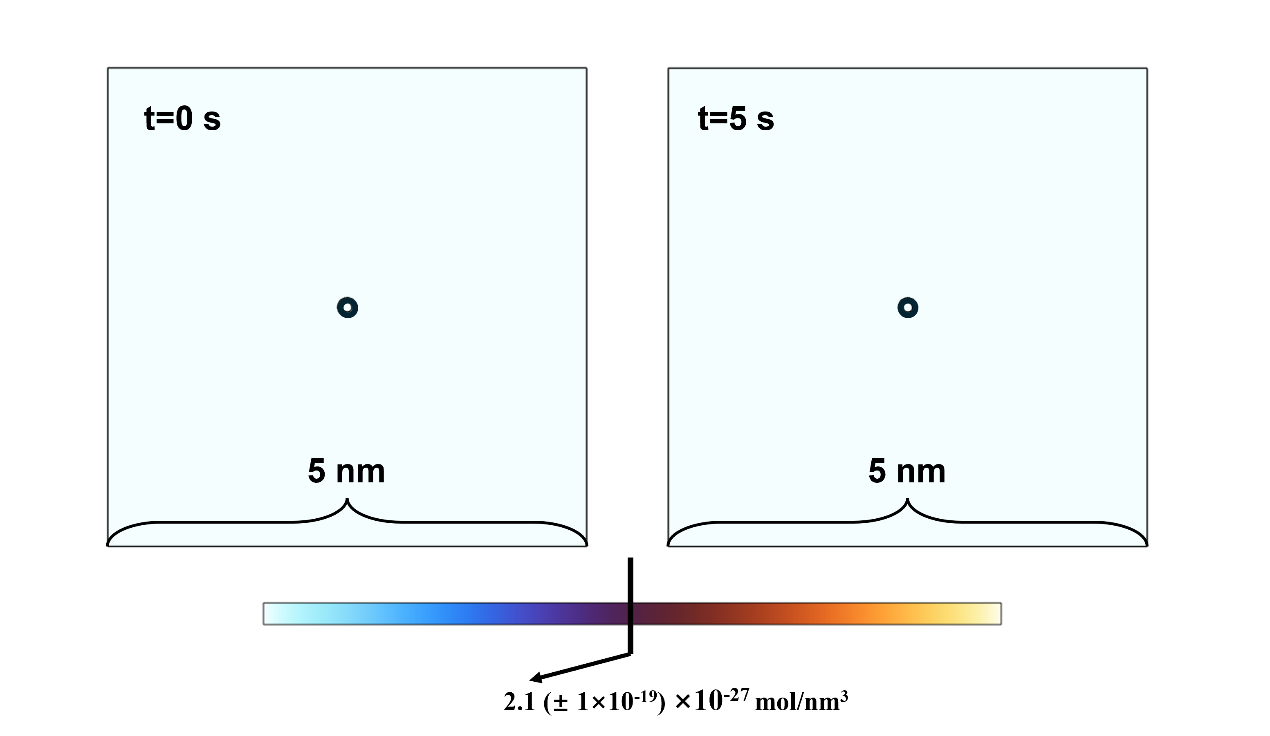


**Figure S47.** “5 nm” model of CO concentration distribution at the 0-second and 5-second moment.


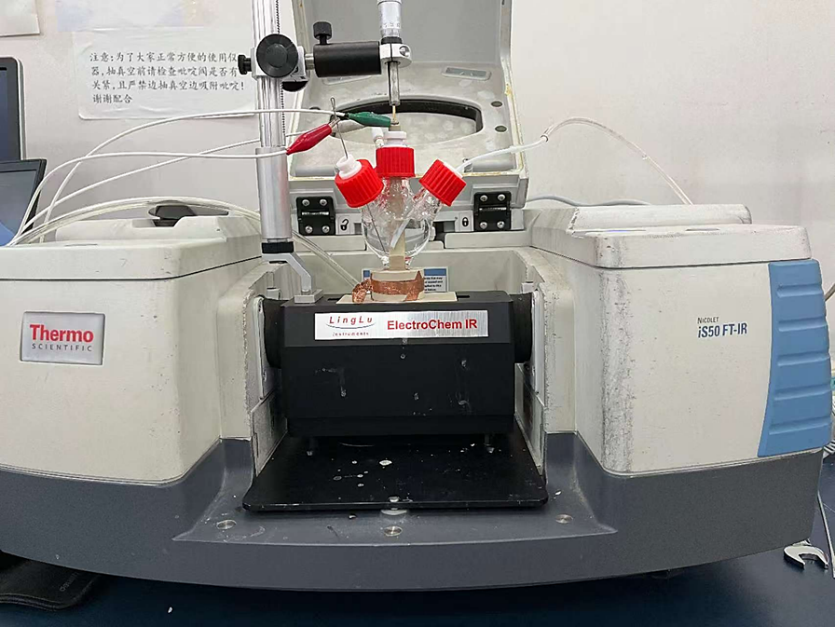


**Figure S48.** In situ electrochemical spectral cell for in situ ATR-IR test.


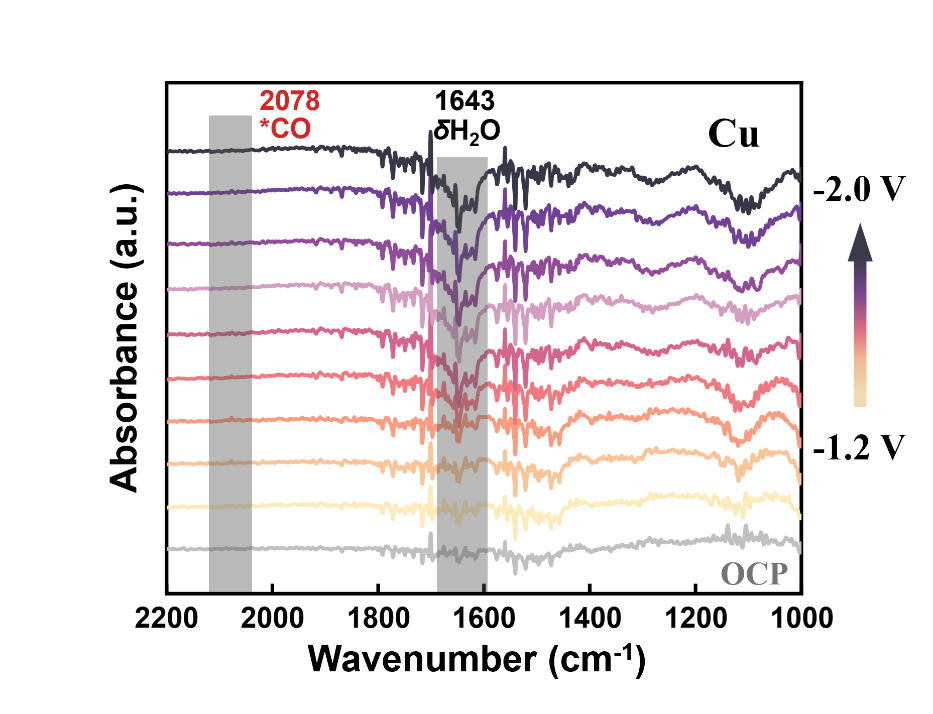


**Figure S49.** In situ ATR-IR spectra of Cu catalyst at different potentials.


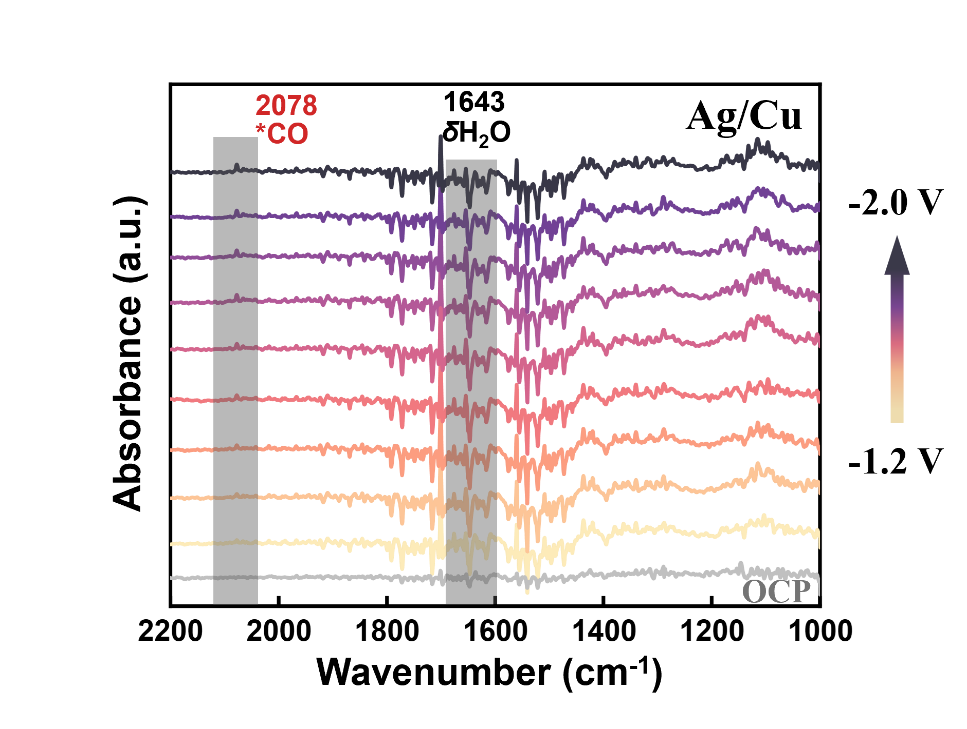


**Figure S50.** In situ ATR-IR spectra of Ag/Cu catalyst at different potentials.


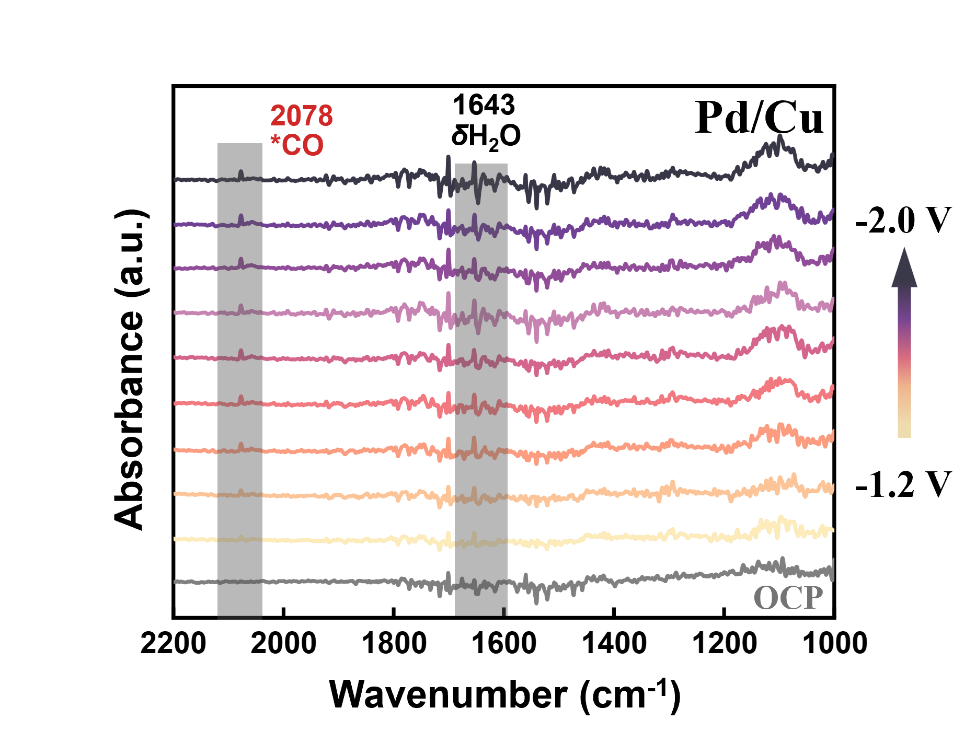


**Figure S51.** In situ ATR-IR spectra of Pd/Cu catalyst at different potentials.


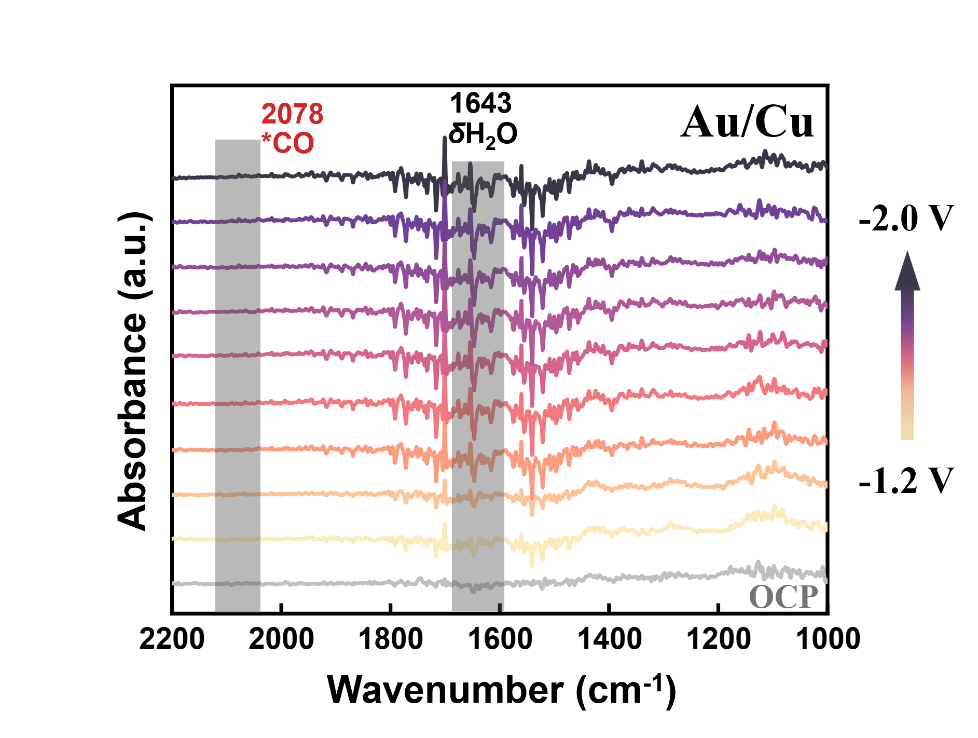


**Figure S52.** In situ ATR-IR spectra of Au/Cu catalyst at different potentials.


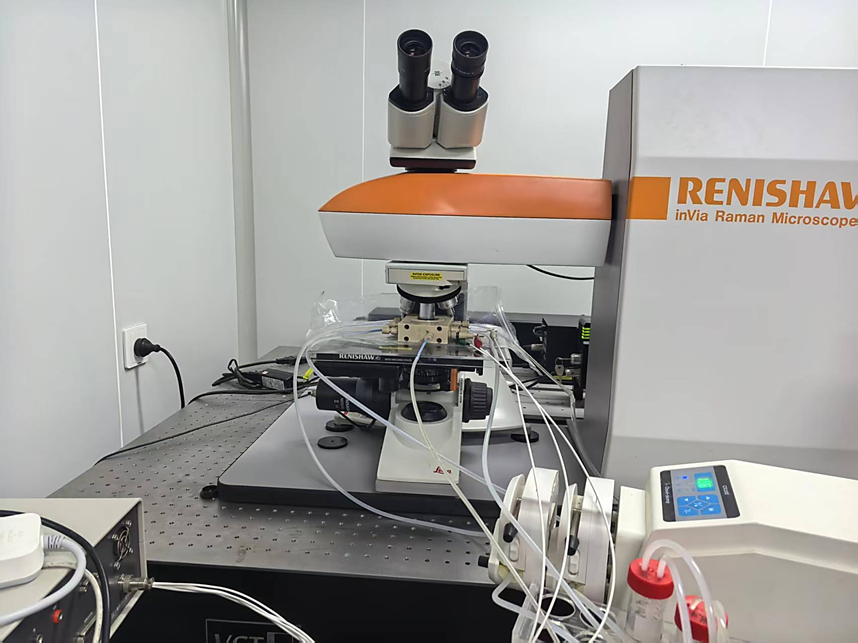


**Figure S53.** Photo in situ Raman reactors for ECR.


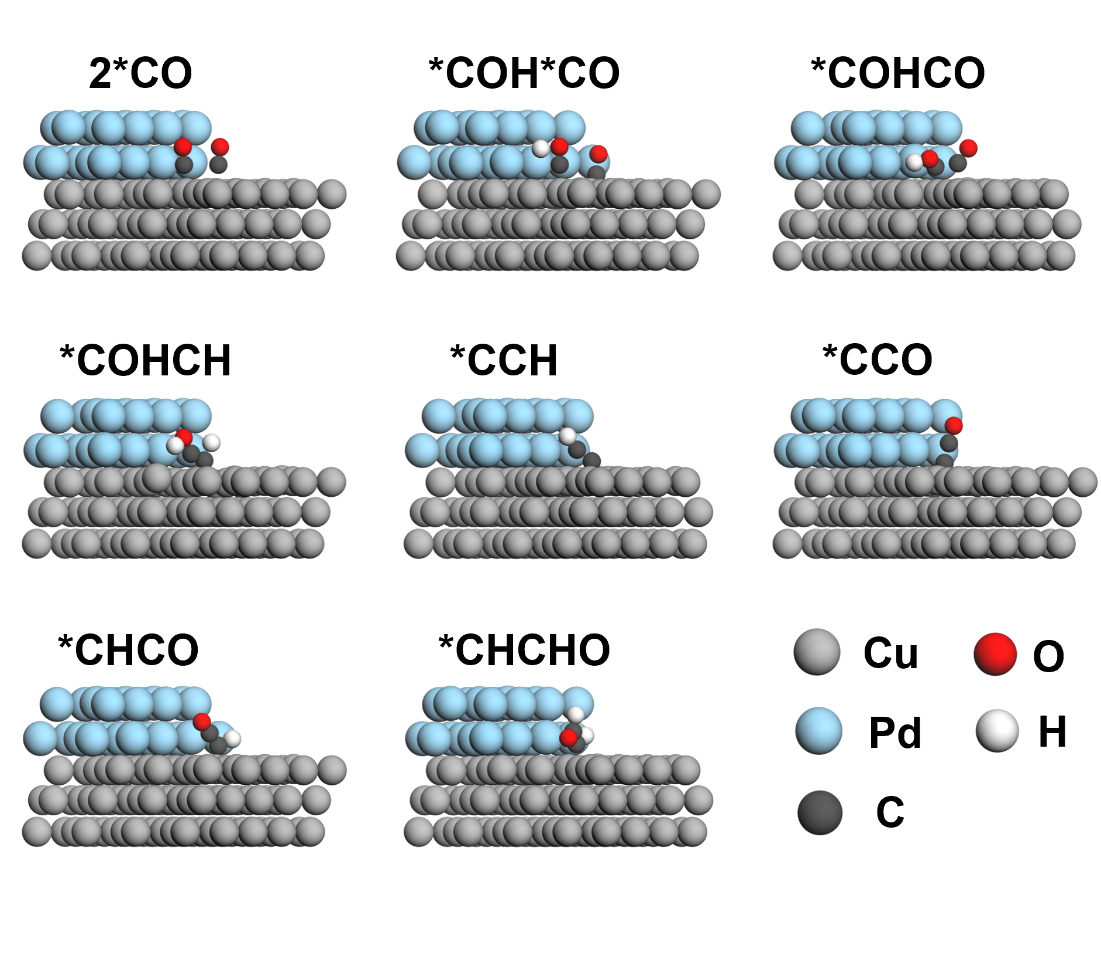


**Figure S54.** The adsorption configurations of various intermediates of CO_2_ for Pd/Cu during the reaction (2*CO, *COH*CO, *COHCO, *COHCH, *CCH, *CCO, *CHCO, and *CHCHO represent adsorbed species).


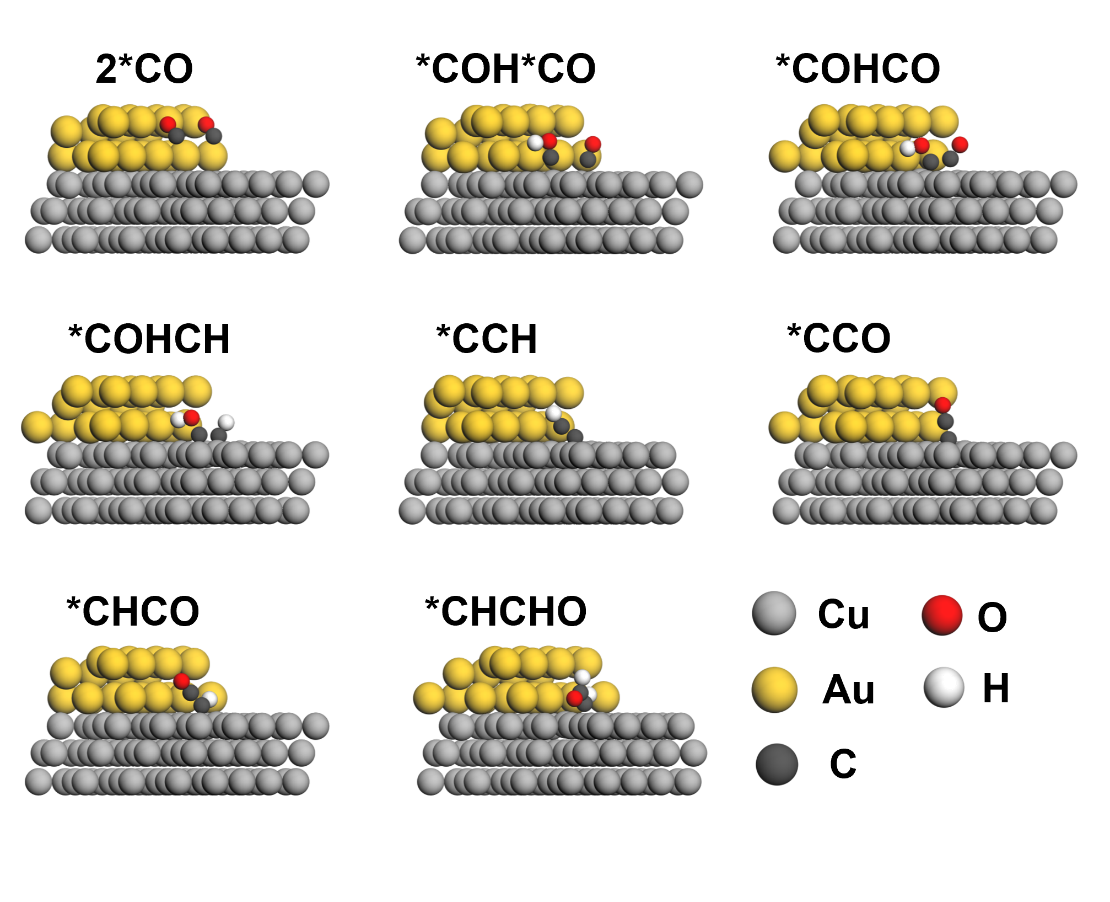


**Figure S****55.** The adsorption configurations of various intermediates of CO_2_ for Au/Cu during the reaction (2*CO, *COH*CO, *COHCO, *COHCH, *CCH, *CCO, *CHCO, and *CHCHO represent adsorbed species).

**Supporting Tables**

**Table S1.** *CO adsorption energy of Cu, Ag/Cu, Pd/Cu, and Au/Cu under different electric field intensities (*CO represents adsorbed species).

| **eV/Å** | 0.00 | 0.05 | 0.10 | 0.15 | 0.20 | 0.25 |
| --- | --- | --- | --- | --- | --- | --- |
| **Cu** (eV) | -1.0178 | -1.0211 | -1.0241 | -1.0272 | -1.0299 | -1.0329 |
| **Ag/Cu** (eV) | -1.1141 | -1.1263 | -1.1287 | -1.1298 | -1.1306 | -1.1309 |
| **Pd/Cu** (eV) | -0.9952 | -0.9977 | -0.9995 | -0.9998 | -1.0001 | -0.9997 |
| **Au/Cu** (eV) | -0.1547 | -0.1586 | -0.1815 | -0.2452 | -0.3077 | -0.2638 |

| **eV/Å** | 0.30 | 0.35 | 0.40 | 0.45 | 0.50 | 0.55 | 0.60 |
| --- | --- | --- | --- | --- | --- | --- | --- |
| **Cu**  (eV) | -1.0357 | -1.039 | -1.0399 | -1.0442 | -1.0468 | -1.0494 | -1.0622 |
| **Ag/Cu** (eV) | -1.1311 | -1.1305 | -1.1297 | -1.1292 | -1.1289 | -1.1252 | -1.1229 |
| **Pd/Cu** (eV) | -0.9981 | -0.9957 | -0.9946 | -0.9944 | -0.9943 | -0.9896 | -0.9848 |
| **Au/Cu** (eV) | -0.2473 | -0.1606 | -0.1639 | -0.1623 | -0.1607 | -0.1648 | -0.1646 |

**Table S2.** *CO Bader charge transfer calculations for Cu, Ag/Cu, Pd/Cu, and Au/Cu under different electric field intensities (*CO represents adsorbed species).

| **eV/Å** | **0.00** | **0.05** | **0.10** | **0.15** | **0.20** | **0.25** |
| --- | --- | --- | --- | --- | --- | --- |
| **Cu**  (e) | 0.26287 | 0.26536 | 0.26579 | 0.26632 | 0.26657 | 0.26706 |
| **Ag/Cu** (e) | 0.26536 | 0.26777 | 0.26844 | 0.26826 | 0.26829 | 0.26868 |
| **Pd/Cu** (e) | 0.22374 | 0.24301 | 0.25236 | 0.25267 | 0.25319 | 0.26005 |
| **Au/Cu** (e) | 0.07132 | 0.07132 | 0.07139 | 0.07140 | 0.07143 | 0.07183 |

| **eV/Å** | **0.30** | **0.35** | **0.40** | **0.45** | **0.50** | **0.55** | **0.60** |
| --- | --- | --- | --- | --- | --- | --- | --- |
| **Cu**  (e) | 0.26751 | 0.26791 | 0.26578 | 0.26886 | 0.26943 | 0.26997 | 0.26960 |
| **Ag/Cu** (e) | 0.26976 | 0.26864 | 0.2688 | 0.26891 | 0.27218 | 0.26833 | 0.26819 |
| **Pd/Cu** (e) | 0.26325 | 0.24509 | 0.24483 | 0.24572 | 0.25431 | 0.25568 | 0.25476 |
| **Au/Cu** (e) | 0.07173 | 0.07181 | 0.07175 | 0.07152 | 0.07172 | 0.07147 | 0.07142 |

**Table S3.** Fitting parameters of Cu K-edge EXAFS spectra of CuO, Ag/CuO, Pd/CuO, and Au/CuO catalysts.

| **Sample** | **Shell** | **Bond length (Å)** | **CN** | **σ^2^ (Å^2^)** | **E_0_ shift (eV)** | **R factor** |
| --- | --- | --- | --- | --- | --- | --- |
|  | Cu-O | 1.95±0.01 | 4.9±0.3 | 0.005±0.001 |  |  |
| CuO | Cu-Cu | 2.96±0.02 | 6.3±0.5 | 0.010±0.007 | -0.3±0.7 | 0.007 |
|  | Cu-Cu | 3.14±0.01 | 2.4±0.1 | 0.005±0.001 |  |  |
|  | Cu-O | 1.95±0.01 | 5.0±0.4 | 0.005±0.001 |  |  |
| Ag/CuO | Cu-Cu | 2.97±0.03 | 6.1±0.5 | 0.012±0.009 | -1.3±0.8 | 0.008 |
|  | Cu-Cu | 3.14±0.01 | 2.8±0.3 | 0.005±0.001 |  |  |
|  | Cu-O | 1.95±0.01 | 5.2±0.3 | 0.005±0.001 |  |  |
| Pd/CuO | Cu-Cu | 2.97±0.02 | 6.4±0.7 | 0.011±0.007 | -0.3±0.7 | 0.006 |
|  | Cu-Cu | 3.14±0.01 | 2.4±0.5 | 0.005±0.001 |  |  |
|  | Cu-O | 1.95±0.01 | 4.8±0.3 | 0.005±0.001 |  |  |
| Au/CuO | Cu-Cu | 2.97±0.02 | 6.2±0.8 | 0.011±0.008 | -1.0±0.7 | 0.007 |
|  | Cu-Cu | 3.14±0.01 | 2.3±0.4 | 0.005±0.001 |  |  |

**Note for Tables S3, S5-S8:** The value of the amplitude reduction factor (S_0_^2^) was fixed to 0.80. Bond length is the interatomic distance. CN is the coordination number. σ^2^ is Debye-Waller factor (a measure of thermal and static disorder in absorber scatter distance). E_0_ shift is edge-energy shift (the difference between the zero kinetic energy value of the sample and that of the theoretical model). R factor is used to value the goodness of the fitting.

**Table S4.** Inductively coupled plasma optical emission spectrometry (ICP) of CuO, Ag/CuO, Pd/CuO, and Au/CuO catalysts.

| Catalyst | Cu (wt%) | Tandem element  (wt%) | Molar ratios by ICP-OES |
| --- | --- | --- | --- |
| CuO | 73.2% | / | / |
| Ag/CuO | 53.6% | Ag (19.3%) | Ag:Cu=1:4.69 |
| Pd/CuO | 54.1% | Pd (19.0%) | Pd:Cu=1:4.72 |
| Au/CuO | 44.3% | Au (29.4%) | Au:Cu=1:4.64 |

**Table S5.** Fitting parameters of Cu K-edge EXAFS spectra with time evolution during ECR over Cu catalyst.

| **Sample** | **Shell** | **Bond length (Å)** | **CN** | **σ^2^ (Å^2^)** | **E_0_ shift (eV)** | **R factor** |
| --- | --- | --- | --- | --- | --- | --- |
| Cu  (10 min) | Cu-Cu | 2.53±0.01 | 10.6±1.0 | 0.009±0.001 | 4.7±0.9 | 0.005 |
| Cu  (20 min) | Cu-Cu | 2.53±0.01 | 10.8±0.9 | 0.009±0.001 | 4.5±0.8 | 0.004 |
| Cu  (30 min) | Cu-Cu | 2.53±0.01 | 10.5±1.0 | 0.009±0.001 | 4.3±0.9 | 0.005 |
| Cu  (40 min) | Cu-Cu | 2.53±0.01 | 10.4±1.0 | 0.009±0.001 | 4.1±0.9 | 0.005 |
| Cu  (50 min) | Cu-Cu | 2.53±0.01 | 10.7±1.0 | 0.009±0.001 | 4.3±0.9 | 0.005 |

**Table S6.** Fitting parameters of Cu K-edge EXAFS spectra with time evolution during ECR over Ag/Cu catalyst.

| **Sample** | **Shell** | **Bond length (Å)** | **CN** | **σ^2^ (Å^2^)** | **E_0_ shift (eV)** | **R factor** |
| --- | --- | --- | --- | --- | --- | --- |
| Ag/Cu  (10 min) | Cu-Cu | 2.53±0.01 | 10.9±0.9 | 0.009±0.001 | 4.3±0.8 | 0.004 |
| Ag/Cu  (20 min) | Cu-Cu | 2.53±0.00 | 11.1±0.8 | 0.009±0.001 | 4.4±0.7 | 0.003 |
| Ag/Cu  (30 min) | Cu-Cu | 2.53±0.01 | 11.2±0.9 | 0.009±0.001 | 4.2±0.7 | 0.003 |
| Ag/Cu  (40 min) | Cu-Cu | 2.53±0.01 | 11.0±0.9 | 0.009±0.001 | 4.5±0.7 | 0.003 |
| Ag/Cu  (50 min) | Cu-Cu | 2.53±0.00 | 11.4±0.8 | 0.009±0.001 | 4.4±0.7 | 0.003 |

**Table S7.** Fitting parameters of Cu K-edge EXAFS spectra with time evolution during ECR over Pd/Cu catalyst.

| **Sample** | **Shell** | **Bond length (Å)** | **CN** | **σ^2^ (Å^2^)** | **E_0_ shift (eV)** | **R factor** |
| --- | --- | --- | --- | --- | --- | --- |
| Pd/Cu  (10 min) | Cu-Cu | 2.53±0.01 | 11.6±1.2 | 0.009±0.001 | 4.3±0.9 | 0.006 |
| Pd/Cu  (20 min) | Cu-Cu | 2.53±0.00 | 11.2±1.4 | 0.009±0.001 | 5.1±1.1 | 0.009 |
| Pd/Cu  (30 min) | Cu-Cu | 2.53±0.01 | 11.0±0.9 | 0.009±0.001 | 5.1±1.0 | 0.004 |
| Pd/Cu  (40 min) | Cu-Cu | 2.53±0.01 | 11.5±1.2 | 0.009±0.001 | 4.5±0.7 | 0.007 |
| Pd/Cu  (50 min) | Cu-Cu | 2.53±0.00 | 11.2±1.0 | 0.009±0.001 | 4.5±0.8 | 0.005 |

**Table S8.** Fitting parameters of Cu K-edge EXAFS spectra with time evolution during ECR over Au/Cu catalyst.

| **Sample** | **Shell** | **Bond length (Å)** | **CN** | **σ^2^ (Å^2^)** | **E_0_ shift (eV)** | **R factor** |
| --- | --- | --- | --- | --- | --- | --- |
| Au/Cu  (10 min) | Cu-Cu | 2.53±0.01 | 10.8±0.9 | 0.009±0.001 | 4.2±0.8 | 0.004 |
| Au/Cu  (20 min) | Cu-Cu | 2.53±0.00 | 10.9±0.9 | 0.009±0.001 | 3.8±0.8 | 0.004 |
| Au/Cu  (30 min) | Cu-Cu | 2.53±0.01 | 11.0±0.8 | 0.009±0.001 | 4.3±0.7 | 0.003 |
| Au/Cu  (40 min) | Cu-Cu | 2.53±0.01 | 11.0±0.9 | 0.009±0.001 | 3.9±0.8 | 0.005 |
| Au/Cu  (50 min) | Cu-Cu | 2.53±0.00 | 10.4±1.0 | 0.009±0.001 | 3.7±1.3 | 0.011 |

**Table S9.** Reaction steps of *CO_2_ to *CO for DFT calculations (*CO_2_ and *CO represent adsorbed species).

| No. | Reaction steps |
| --- | --- |
| R1 | *CO_2_ + 2H^+^ + 2e^-^ |
| R2 | *CO + H_2_O |

**Table S10.** Gibbs free energy and ∆G of *CO_2_ to *CO step for Ag and Ag/Cu under 0.3 eV/Å electric field (*CO_2_ and *CO represent adsorbed species).

| Ag | R1 (eV) | R2 (eV) | Ag/Cu | R1 (eV) | R2 (eV) |
| --- | --- | --- | --- | --- | --- |
| G | -95.96 | -95.54 | G | -466.63 | -465.65 |
| ∆G | / | 0.42 | ∆G | / | 0.98 |

**Table S11.** Gibbs free energy and ∆G of *CO_2_ to *CO step for Pd and Pd/Cu under 0.3 eV/Å electric field (*CO_2_ and *CO represent adsorbed species).

| Pd | R1 (eV) | R2 (eV) | Pd/Cu | R1 (eV) | R2 (eV) |
| --- | --- | --- | --- | --- | --- |
| G | -157.71 | -157.37 | G | -542.50 | -541.57 |
| ∆G | / | 0.34 | ∆G | / | 0.93 |

**Table S12.** Gibbs free energy and ∆G of *CO_2_ to *CO step for Au and Au/Cu under 0.3 eV/Å electric field (*CO_2_ and *CO represent adsorbed species).

| Au | R1 (eV) | R2 (eV) | Au/Cu | R1 (eV) | R2 (eV) |
| --- | --- | --- | --- | --- | --- |
| G | -100.84 | -100.14 | G | -468.24 | -467.18 |
| ∆G | / | 0.7 | ∆G | / | 1.06 |

**Table S13.** C_2+_ products of Faraday efficiency in this work and recently reported materials.

| Electrocatalysts | Cell type | FE_C2+_ (%) | References |
| --- | --- | --- | --- |
| Ag/Cu | Flow cell | 89.2 | This work. |
| aw=0.66-CuNP | Flow cell | 73.0 | *Nat. Catal.* **2023**, 6, 807^[5]^ |
| M-Cu_1_/CuNP | Flow cell | 75.4 | *Nat. Commun.* **2023**, *14*, 4615^[6]^ |
| Cu_1_Ni-BDP MOF | Flow cell | 66.0 | *J. Am. Chem. Soc.* **2023**, *145*, 26444^[7]^ |
| Cu (OH) BTA | Flow cell | 73.0 | *Nat. Commun.* **2023**, *14*, 474^[8]^ |
| Cu_9_Ga_4_ | Flow cell | 71.0 | *Nat. Catal.* **2023**, *6*, 807^[9]^ |
| CuGa | Flow cell | 76.9 | *J. Am. Chem. Soc.* **2023**, *145*, 4675^[10]^ |
| OD-Cu-III | Flow cell | 74.9 | *J. Am. Chem. Soc.* **2022**, *144*, 259^[11]^ |
| Inz-Cu_3_ | Flow cell | 66.8 | *ACS Catal.* **2024**, *14*, 741^[12]^ |
| Hierarchical Cu dendrites | Flow cell | 64.0 | *J. Am. Chem. Soc.* **2021**, *143*, 8011^[13]^ |
| hollow-fiber Cu | Flow cell | 73.4 | *Energy Environ. Sci.* **2024**, *17*, 510^[14]^ |
| CuODA_2_ | Flow cell | 73.5 | *Adv. Funct. Mater.* **2024**, *34*, 2404566^[15]^ |
| Cu=N catalyst | Flow cell | 75.0 | *J. Am. Chem. Soc.* **2024**, *146*, 14260^[16]^ |
| CuO-BPNF | Flow cell | 74.7 | *Adv. Energy Mater.* **2024**, *14*, 2303118^[17]^ |
| Pot-Cu | Flow cell | 82.5 | *J. Am. Chem. Soc.* **2024**, *146*, 15917^[18]^ |
| Cu-Au Janus | Flow cell | 80.0 | *Chem* **2024**, *10*, 2745^[19]^ |
| La-Cu | Flow cell | 86.2 | *J. Am. Chem. Soc.* **2024**, *15*, 4821^[20]^ |
| AgCu-SAA | Flow cell | 83.4 | *J. Am. Chem. Soc.* **2025**^[21]^ |

**Table S14.** Reaction steps of *CO to ethylene and ethanol for DFT calculations (2*CO, *COH*CO, *COHCO, *COHCH, *CCH, *CCO, *CHCO, and *CHCHO represent adsorbed species).

| No. | Reaction steps |
| --- | --- |
| Ethylene |  |
| R3 | *CO*CO + 5H^+^ + 5e^-^ |
| R4 | *COH*CO + 4H^+^ + 4e^-^ |
| R5 | *COHCO + 4H^+^ + 4e^-^ |
| R6 | *COHCH + H_2_O + H^+^ + e^-^ |
| R7 | *CCH + 2H_2_O |
| Ethanol |  |
| R8 | *CO*CO + 4H^+^ + 4e^-^ |
| R9 | *COH*CO + 3H^+^ + 3e^-^ |
| R10 | *COHCO + 3H^+^ + 3e^-^ |
| R11 | *CCO + H_2_O + 2H^+^ + 2e^-^ |
| R12 | *CHCO + H_2_O + H^+^ + e^-^ |
| R13 | *CHCHO + H_2_O |

**Table S15.** Entropy, zero-point vibration energy and Gibbs free energy for H_2_ and H_2_O.

|  | E (eV) | E_ZPE_ (eV) | TS (eV) | G (eV) |
| --- | --- | --- | --- | --- |
| H_2_ | -6.76 | 0.39 | 0.40 | -6.77 |
| H_2_O | -14.22 | 0.57 | 0.67 | -14.32 |

**Table S16.** Gibbs free energy and ∆G of each reaction step for Ag/Cu.

| Ag/Cu | R3 (eV) | R4 (eV) | R5 (eV) | R6 (eV) | R7 (eV) |
| --- | --- | --- | --- | --- | --- |
| G | -483.01 | -484.05 | -483.73 | -484.66 | -485.07 |
| ∆G | / | -1.04 | 0.31 | -0.93 | -0.41 |

| Ag/Cu | R8 (eV) | R9 (eV) | R10 (eV) | R11 (eV) | R12 (eV) | R13 (eV) |
| --- | --- | --- | --- | --- | --- | --- |
| G | -479.62 | -480.66 | -480.35 | -481.55 | -481.68 | -481.68 |
| ∆G | / | -1.04 | 0.31 | -1.20 | -0.13 | -0.17 |

**Table S17.** Gibbs free energy and ∆G of each reaction step for Pd/Cu.

| Pd/Cu | R3 (eV) | R4 (eV) | R5 (eV) | R6 (eV) | R7 (eV) |
| --- | --- | --- | --- | --- | --- |
| G | -559.40 | -558.3 | -558.07 | -555.27 | -559.58 |
| ∆G | / | 1.10 | 0.23 | 2.80 | -4.32 |

| Pd/Cu | R8 (eV) | R9 (eV) | R10 (eV) | R11 (eV) | R12 (eV) | R13 (eV) |
| --- | --- | --- | --- | --- | --- | --- |
| G | -556.01 | -554.92 | -554.69 | -556.16 | -556.27 | -556.55 |
| ∆G | / | 1.10 | 0.23 | -1.17 | -0.11 | -0.28 |

**Table S18.** Gibbs free energy and ∆G of each reaction step for Au/Cu.

| Au/Cu | R1 (eV) | R2 (eV) | R3 (eV) | R4 (eV) | R5 (eV) |
| --- | --- | --- | --- | --- | --- |
| G | -484.96 | --486.34 | -485.16 | -486.55 | -486.63 |
| ∆G | / | -1.38 | 1.18 | -1.39 | -0.08 |

| Au/Cu | R6 (eV) | R7 (eV) | R8 (eV) | R9 (eV) | R10 (eV) | R11 (eV) |
| --- | --- | --- | --- | --- | --- | --- |
| G | -481.57 | -482.96 | -481.78 | -483.2 | -483.34 | -483.23 |
| ∆G | / | -1.38 | 1.18 | -1.42 | -0.14 | 0.11 |

1. a) S. Grimme, J. Antony, S. Ehrlich, H. Krieg, *J. Chem. Phys.* **2010**, *132*, 154104. b) G. Kresse, J. Furthmüller, *Comp. Mater. Sci.* **1996**, *6*, 15.
2. Y. Zhang, W. Yang, *Phys. Rev. Lett.* **1998**, *80*, 890.
3. S. Grimme, J. Antony, S. Ehrlich, H. Krieg, *J. Chem. Phys.* **2010**, *132*, 154104.
4. J. K. Nørskov, J. Rossmeisl, A. Logadottir, L. Lindqvist, J. R. Kitchin, T. Bligaard, H. Jo´nsson, *J. Phys. Chem. B* **2004**, *108*, 17886.
5. H. Zhang, J. Gao, D. Raciti, A. S. Hall, *Nat. Catal*. **2023**, *6*, 807.
6. J. Feng, L. Zhang, S. Liu, L. Xu, X. Ma, X. Tan, L. Wu, Q. Qian, T. Wu, J. Zhang, X. Sun, B. Han, *Nat. Commun*. **2023**, *14*, 4615.
7. L. Huang, Z. Liu, G. Gao, C. Chen, Y. Xue, J. Zhao, Q. Lei, M. Jin, C. Zhu, Y. Han, J. S. Francisco, X. Lu, *J. Am. Chem. Soc*. **2023**, *145*, 26444.
8. Y. Liang, J. Zhao, Y. Yang, S.-F. Hung, J. Li, S. Zhang, Y. Zhao, A. Zhang, C. Wang, D. Appadoo, L. Zhang, Z. Geng, F. Li, J. Zeng, *Nat. Commun*. **2023**, *14*, 474.
9. Zhang, H.; Gao, J.; Raciti, D.; Hall, A. S. Promoting Cu-Catalysed CO_2_ Electroreduction to Multicarbon Products by Tuning the Activity of H_2_O. *Nat. Catal*. **2023**, *6*, 807.
10. P. Li, J. Bi, J. Liu, Y. Wang, X. Kang, X. Sun, J. Zhang, Z. Liu, Q. Zhu, B. Han, *J. Am. Chem. Soc*. **2023**, *145*, 4675.
11. Z.-Z. Wu, X.-L. Zhang, Z.-Z. Niu, F.-Y. Gao, P.-P. Yang, L.-P. Chi, L. Shi, W.-S. Wei, R. Liu, Z. Chen, S. Hu, X. Zheng, M.-R. Gao, *J. Am. Chem. Soc*. **2022**, *144*, 259.
12. R. Wang, L.-Z. Dong, J.-W. Shi, M. Zhang, S.-L. Li, Y.-Q. Lan, J. Liu, *ACS Catal*. **2024**, *14*, 741.
13. Z.-Z. Niu, F.-Y. Gao, X.-L. Zhang, P.-P. Yang, R. Liu, L.-P. Chi, Z.-Z. Wu, S. Qin, X. Yu, M.-R. Gao, *J. Am. Chem. Soc*. **2021**, *143*, 8011.
14. C. Zhu, G. Wu, A. Chen, G. Feng, X. Dong, G. Li, S. Li, Y. Song, W. Wei, W. Chen, *Energy Environ. Sci*. **2024**, *17*, 510.
15. J. Hu, S. Osella, J. Albero, H. García, *Adv. Funct. Mater.* **2024**, *34*, 2404566.
16. Z. Liu, L. Song, X. Lv, M. Liu, Q. Wen, L. Qian, H. Wang, M. Wang, Q. Han, G. Zheng, *J. Am. Chem. Soc.* **2024**, *146*, 14260.
17. F. Pan, X. Duan, L. Fang, H. Li, Z. Xu, Y. Wang, T. Wang, T. Li, Z. Duan, K.-J. Chen, *Adv. Energy Mater.* **2024**, *14*, 2303118.
18. J. Jiao, X. Kang, J. Yang, S. Jia, Y. Peng, S. Liu, C. Chen, X. Xing, M. He, H. Wu, B. Han, *J. Am. Chem. Soc.* **2024**, *146*, 15917.
19. T. Zhang, B. Zhang, Y. Zang, P. Zeng, Y. Li, H. J. Fan, *Chem* **2024**, 10, 2745.
20. J. Feng, L. Wu, X. Song, L. Zhang, S. Jia, X. Ma, X. Tan X. Kang, Q. Zhu, X. Sun, B. Han, *J. Am. Chem. Soc*. **2024**, *15*, 4821.
21. M. Wang, M Fang, Y. Liu, C. Chen, Y. Zhang, H. Wu, M. He, B. Han, *J. Am. Chem. Soc*. **2025**, 147, 16450-16458.
